# Mobile Health (mHealth) Apps in Sport Training: A Scoping Review

**DOI:** 10.3390/s26175394

**Published:** 2026-08-26

**Authors:** Junyan Liu, Yiwen Dong, Ian Brooks, Waifong Catherine Cheung, Vu Linh Nguyen, Yih-Kuen Jan

**Affiliations:** 1 Department of Health and Kinesiology, University of Illinois at Urbana-Champaign, Urbana, IL 61801, USA; 2 Department of Industrial & Enterprise Systems Engineering, University of Illinois at Urbana-Champaign, Urbana, IL 61801, USA; 3 School of Information Science, University of Illinois at Urbana-Champaign, Champaign, IL 61820, USA; 4 Doctor of Physical Therapy Program, Northern Illinois University, DeKalb, IL 60115, USA; wcheung@niu.edu; 5 College of Engineering and Computer Science, VinUniversity, Hanoi 100000, Vietnam

**Keywords:** athlete monitoring, digital health, injury risk assessment, load management, smartphone sensors

## Abstract

Mobile health (mHealth) apps increasingly capture the physiological, biomechanical, and psychological variables involved in sport training, but the evidence remains fragmented across single-domain reviews, leaving practitioners without a consolidated basis for selecting and deploying these tools across the training process. This scoping review aimed to identify and characterize research on mHealth apps in sport training, focusing on their performance testing, training load and recovery monitoring, technical and skill development, injury screening and prevention, and athlete self-management. It also synthesized evidence regarding their applications, intended purposes, technical characteristics, and the evidence supporting their effectiveness. Five databases (PubMed, Scopus, Web of Science, SPORTDiscus, Embase) were searched from inception to July 2026 for journal articles reporting original empirical data on app research on the sport training process in athletes. Studies involving only the promotion of physical activity or lacking human-subject testing, including commercially available apps without supporting research on their effectiveness, were excluded. Findings were synthesized narratively, and methodological quality was appraised with the Mixed Methods Appraisal Tool. Of 9476 records identified, 111 studies met the inclusion criteria and were inductively classified into ten application categories: sport skill training (*n* = 26), performance measurement (*n* = 20), vertical jump measurement (*n* = 18), self-reported monitoring (*n* = 12), physiological measurement (*n* = 12), musculoskeletal screening (*n* = 10), psychological intervention (*n* = 5), nutrition (*n* = 3), anthropometric and maturation screening (*n* = 3), and tactical and match analysis (*n* = 2). Most apps relied on built-in smartphone sensors or no sensing at all and used manual or deterministic computation; processing location went unreported in 74.8% of studies, which reflects a reporting gap rather than an architectural profile of the field, and reported that AI or machine learning labels did not track with actual method disclosure. Validation and reliability designs dominated the evidence base (52%), while randomized or controlled effectiveness trials were rare (10%). Apps generally showed good relative validity but limited absolute accuracy against criterion instruments, and wherever apps were deployed longitudinally, adherence rather than accuracy determined their real-world value. mHealth apps now support nearly every stage of sport training and can substitute for laboratory instruments in select, validated use cases, including video-based sprint and jump timing and chest-strap-paired heart-rate variability monitoring. However, the field remains organized around demonstrating measurement accuracy rather than showing that app-guided decisions improve athlete outcomes. A successful pathway for mHealth app development should progress from technical validity, through measurement reliability and responsiveness, to decision rules, and then to practitioner adoption by coaches and athletes, ultimately yielding better athlete outcomes.

## 1. Introduction

Mobile health (mHealth) apps are software applications that run on a mobile platform such as a smartphone or tablet, and they have become widely used tools for monitoring health and physical activity [1]. Smartphones, the most common platform for these apps, have become one of the most rapidly and widely adopted technologies in the world, with an estimated 5.78 billion people, or roughly 70% of the global population, owning a device by early 2026 [2]. Objective screen-time data from a large US adult population indicate that individuals now spend a mean of approximately 391 min per day on their smartphones, underscoring how central these devices have become to everyday life [3]. The health and fitness sector has seen especially rapid growth, with more than 200,000 health and fitness applications available across the major app stores by 2024 [4]. Within sport and exercise specifically, mHealth apps can now capture many of the physiological and biomechanical variables that were traditionally monitored only in specialized laboratory or clinical settings [5]. In athlete populations, integrating app-based monitoring into daily training has been proposed as a way to track load and recovery and to make individualized, responsive adjustments to exercise [6].

In modern athletic training, coaches and support staff systematically monitor training load to determine whether an athlete is adapting and to minimize the risk of non-functional overreaching, illness, or injury [7]. This has made the quantification of both external load (the work performed) and internal load (the physiological and perceptual response it elicits) a central concern in sport science [8]. Because recovery must be balanced against accumulated training and competition stress, the systematic monitoring of recovery is likewise regarded as essential for maximizing performance and preventing negative outcomes such as under-recovery, overtraining, injury, and illness [9]. Beyond internal load and recovery, the training process also depends on the objective assessment of physical performance qualities such as lower-limb power, for which force platforms represent the gold standard; however, these instruments are largely confined to research laboratories or elite facilities by their cost and complexity, limiting everyday use [10]. This has driven growing interest in affordable, portable alternatives that can capture comparable information in the field, including markerless solutions that rely on readily available devices such as smartphones and low-cost cameras [11].

Building on this accessibility, a substantial body of research has demonstrated the validity of mHealth apps for quantifying discrete performance metrics, including vertical jump height, sprint split times, barbell movement velocity, and change-of-direction speed [12,13,14,15]. A further group of mHealth apps focuses on internal physiological load, most prominently by using smartphone-based measures of heart-rate variability to assess training and recovery status [16,17]. Complementing these objective tools, subjective self-report mHealth apps capture athlete wellness, perceived exertion, and readiness. Evidence from systematic reviews indicates that such subjective measures can reflect training-induced changes in athlete well-being and demonstrate consistency comparable to, and in some cases exceeding, that of commonly used objective markers [18]. Other mHealth apps support skill and technique development, pairing the smartphone camera with pose-estimation or motion-analysis algorithms to analyze movement quality and provide feedback during tasks such as running gait or sport-specific technique [19,20]. Broader still are mHealth apps for musculoskeletal injury risk screening and prevention, dietary and nutritional monitoring, and psychological support, such as relaxation and stress management, among other emerging uses [21,22,23,24].

Prior reviews in this area have typically focused on a single measurement domain, such as the validity and reliability of mHealth apps, for assessing strength, power, velocity, and change-of-direction performance [25]. Others have been limited to a single technological modality, such as video-based motion-capture apps for assessing motor performance skills, and have included only a small number of studies drawn from heterogeneous populations, including healthy adults, older adults, and athletes [26]. Still others have examined camera-based movement screening in healthy adults, or the assessment of cardiorespiratory fitness across combined clinical and sporting contexts, rather than sport-specific training [4,27]. Where broader syntheses of athlete-monitoring technology exist, they have generally concentrated on wearable devices such as heart-rate monitors, inertial measurement units (IMUs), and global positioning systems (GPSs) rather than on mHealth apps [28]. Although a small number of broader overviews of athlete-focused mHealth apps have been published, these have primarily emphasized general benefits, implementation challenges, and data-privacy considerations rather than systematically cataloging the specific apps and their supporting evidence [29]. To the best of our knowledge, no review has yet comprehensively mapped the full range of mHealth apps used across the sport-training process within athlete and sport-training populations.

A consolidated overview is therefore needed to establish which mHealth apps are used in sport training, the purposes for which they are used, and the evidence supporting their effectiveness. Because the objective was to map an emerging and heterogeneous body of literature and to identify knowledge gaps, rather than to answer a single, narrowly defined question about effectiveness, a scoping review approach to the literature was adopted [30]. The aim of this review was to comprehensively identify and characterize mHealth apps research in sport-training contexts and to synthesize the available evidence regarding their applications, purposes, and supporting evidence. Specifically, this review aimed to: (1) identify the mHealth apps research reported in the literature and classify them according to their primary application within the training process; (2) describe the sporting contexts in which they have been investigated, including sport type, competitive level, and training setting; (3) characterize the study designs, participant populations, and outcomes assessed; (4) synthesize the evidence regarding their validity, reliability, and effectiveness; and (5) identify knowledge gaps to inform future research and practice.

## 2. Methods

### 2.1. Study Design

This study was conducted as a scoping review, an approach suited to mapping the range and nature of evidence in an emerging and heterogeneous field and to identifying gaps that can guide future research [30]. Reporting followed the PRISMA extension for scoping reviews (PRISMA-ScR; [31]), and a completed checklist is provided in the Supplementary Materials (Table S5). Although risk-of-bias or quality appraisal is not a mandatory element of scoping review methodology, this review additionally appraised methodological quality using a design-specific instrument (Section 2.8) to characterize the evidence base more fully. The review was registered on the Open Science Framework (https://doi.org/10.17605/OSF.IO/BZH6J).

### 2.2. Search Strategy

The literature search was conducted across five electronic databases: PubMed, Scopus, Web of Science (Core Collection), SPORTDiscus (via EBSCO), and Embase. IEEE Xplore and ACM Digital Library were not searched directly because this review’s eligibility criteria excluded conference proceedings, which dominate indexing in both databases; full peer-reviewed journal articles indexed in these databases were still eligible for inclusion via the other five databases and reference list screening. This choice may nonetheless have under-sampled the computer-science and engineering literature relative to the health and sport-science literature, a disciplinary bias we discuss as a limitation in Section 4.10 and that may partly explain the limited algorithmic disclosure reported in Section 3.2. The search combined three concept blocks with the Boolean operator AND: a sport or athlete population block (e.g., sport*, athlete*), a mobile technology block (e.g., “mobile app*”, smartphone, mHealth), and a function or purpose block (e.g., training, performance, monitoring, injury prevention). Within each block, synonyms and related terms were combined with OR. General physical activity and fitness terms were excluded from the function block, for the reasons given in Section 2.3. Search terms were mapped to database-specific controlled vocabulary (e.g., MeSH in PubMed) where available and applied to the title, abstract, and keyword fields, or the equivalent option in each database. The search was limited to English-language records and covered the period from database inception to 12 July 2026. The full search strings for each database are provided in the Supplementary Materials (Table S1). Reference lists of included articles were screened manually to identify additional eligible studies.

### 2.3. Eligibility Criteria

Studies were eligible for inclusion if they (1) investigated a software application running on a smartphone, tablet, or wearable-paired mobile device; (2) evaluated an app used to deliver, support, or measure a function within the sport-training process, including performance testing, training load or recovery monitoring, technical or skill development, injury screening or prevention, or athlete self-management; (3) involved athlete or sport-training populations, from recreationally active individuals engaged in structured training to elite athletes; and (4) reported original empirical data on the app, such as its validity, reliability, effectiveness, feasibility, or usability. Apps paired with external sensors or wearables were eligible, provided the app itself was the primary technology under study, and development or prototype studies were eligible when they reported an empirical evaluation of the app.

Studies were excluded if (1) sport was not the focus of the app or study; (2) the technology evaluated was not an app (e.g., text messaging, video games, telemedicine platforms, standalone wearable sensors, or desktop programs); (3) the app addressed general physical activity or fitness rather than a sport- or athlete-specific context, as such apps have been synthesized in existing reviews; (4) the article was a review, meta-analysis, editorial, opinion piece, conference abstract, case report, thesis, or dissertation; (5) the article described app design or development without any empirical evaluation in human participants; (6) it was a study protocol without reported results; or (7) it was a duplicate record.

### 2.4. Study Selection

**Figure 1:**
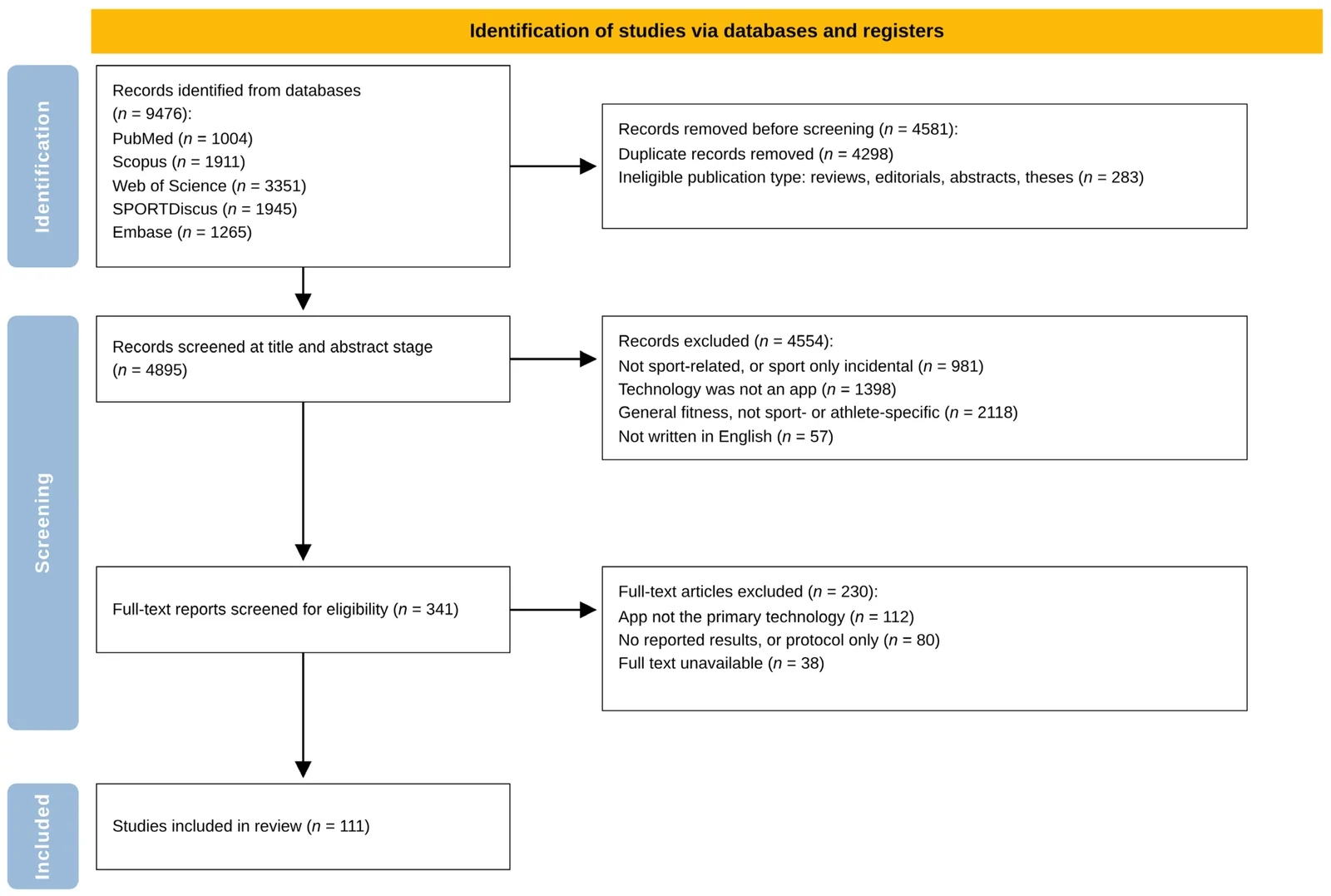
PRISMA flow diagram.

The search identified 9476 records, which were imported into EndNote 21 (Clarivate, Philadelphia, PA, USA). After removal of duplicates and records of ineligible publication type, 4895 unique records were screened by title and abstract by the first author, and the full texts of 341 records were assessed for eligibility. A record whose eligibility was uncertain at either stage was discussed with the corresponding author (Y.-K.J.), who served as arbiter, and final decisions were reached by consensus. A total of 111 studies met all eligibility criteria and were included in the review. The study selection process, including reasons for exclusion at the full-text stage, is summarized in Figure 1.

### 2.5. Data Extraction

A standardized data extraction form was developed in Microsoft Excel (Microsoft Corporation, Redmond, WA, USA) and used to extract data from each included study across six domains: bibliographic information (e.g., first author, year, country); app characteristics (e.g., app name, platform, primary purpose, key features, connected devices); sporting context (e.g., sport type, competitive level, training context and phase); study design and sample characteristics (e.g., design, randomization, sample size, participant description); outcomes and findings (e.g., primary outcomes, key results, statistical significance, author conclusions, and limitations); and the primary application category, as described in Section 2.6. Data were extracted by the first author, and fields for which a study provided no relevant information were recorded as “not reported.” The app-characteristic fields extracted here were subsequently used to code the technical attributes described in Section 2.7.

### 2.6. App Classification

Each included app was assigned to a single primary application category reflecting its principal function within the sport-training process. Because no established classification of sport-training apps was available, the categories were derived inductively from the extracted data: the first author assigned each app a provisional category based on its stated primary purpose and features, and related labels were grouped and refined iteratively into a set of categories for all included studies, that is, one study can only be assigned to one category, with borderline cases resolved in discussion with the corresponding author. Apps supporting more than one function were assigned to the category corresponding to their primary purpose in the source study, so that each app appears in one category only. Under this purpose, the created categories may not be at the same level in terms of performance or function. The categories resulting from this process, their definitions, and their distribution across the included studies are reported in Section 3.1.

To capture use patterns not evident from the primary category alone, two additional non-exclusive codes were applied to each app after data extraction, using the same purpose, features, and testing protocol text already extracted (Section 2.5) rather than conducting a new literature search. The intended operator recorded who physically operates the app or device during data collection (athlete self-operated, coach/practitioner-operated, or both), assigned from category-level default rules refined against explicit language distinguishing user-facing from practitioner-facing design; results are reported in Section 3.2. Secondary function recorded, independent of primary category, whether the app served a measurement, feedback, intervention, or decision-support role, with more than one function coded where the source study described it; results are reported in Section 3.1 and Table S6. Both codes were applied by the first author, with borderline cases resolved in discussion with the corresponding author.

### 2.7. App Technical Characterization

Each study was additionally characterized across three technical domains: sensing requirements, computational approach, and processing location. The coding scheme was developed after data extraction and applied to the app-characteristic data already extracted from each full text (Section 2.5). App store listings and vendor websites were not consulted, as these describe an app’s current release rather than the version evaluated, a distinction that is material to the version-specific validity observed in this review. Coding was performed by the first author, with borderline cases resolved in discussion with the corresponding author.

Sensing was coded as modality, a multi-label variable using a controlled vocabulary (camera video, camera photoplethysmography, microphone, accelerometer, gyroscope, inertial measurement unit, GPS or GNSS, LiDAR, touchscreen, and external sensor categories), and as sensor source in four mutually exclusive categories: no sensing (manual entry only), built-in sensors only, external paired sensor only, or built-in plus external. Instruments serving only as criterion standards, such as force platforms, timing gates, and dual-energy X-ray absorptiometry, were not coded as app sensors, nor was hardware that only delivered output or performed computation.

The computational approach was coded into six mutually exclusive classes reflecting each app’s primary analytic step: (1) manual digitization, in which the user selects the frames or landmarks and the app performs arithmetic only; (2) deterministic or rule-based computation; (3) computer vision or pose estimation, method reported; (4) machine learning or pattern recognition, method reported; (5) automated processing, method not reported; and (6) a large language model or generative component. Separate binary fields recorded whether the source study used artificial intelligence, machine learning, or deep learning terminology, and whether a large language model or agentic component was present. The terminology field was kept independent of the computational class so that stated and disclosed methods could be compared. To our knowledge, no established international standard exists for classifying the computational methods used in consumer-facing sports mHealth apps; like the primary application category scheme (Section 2.6), this six-class scheme was therefore derived inductively from the extracted app-characteristic data rather than adopted from an external standard.

Processing location was coded as on-device, server or cloud, hybrid, or not reported, and was coded as stated only on explicit evidence, such as a named portal, remote data transmission, or an on-device inference framework.

### 2.8. Data Synthesis

Given the descriptive aim of the review and the heterogeneity of the included studies in design, apps, populations, and outcomes, the data were not suitable for meta-analysis and were instead synthesized narratively; differences in reference standards, testing protocols, and outcome definitions precluded meaningful statistical pooling. Pooling was additionally considered within app families evaluated for a common outcome (e.g., the My Jump family for jump height), but was not undertaken because studies varied in criterion instrument, jump type, and population even within the same app family, precluding a statistically defensible pooled estimate. Studies were grouped by the ten application categories, and within each category, the evidence on app validity, reliability, effectiveness, feasibility, and usability was summarized qualitatively, with quantitative findings reported descriptively using the metrics of the primary studies (e.g., correlation coefficients, intraclass correlation coefficients, and mean differences). Patterns, differences, and gaps across categories were then identified to address the review objectives.

The technical characteristics of the included apps were summarized descriptively as frequencies across the ten application categories, and the reported terminology was cross-tabulated against the coded computational class to compare stated and disclosed methods. No inferential testing was applied to these comparisons. More broadly, comparative statements made throughout the Discussion are descriptive and directional rather than statistically normalized: because application categories differ in outcome metric, study design mix, and sample size, no single effect size could be computed across them, and such statements should be read alongside the design-specific quality appraisal reported in Supplementary Table S3 rather than as a statistically weighted comparison. The methodological quality of the included studies was appraised using the Mixed Methods Appraisal Tool (MMAT, version 2018), which applies design-specific criteria across randomized, non-randomized, quantitative descriptive, qualitative, and mixed-method studies [32]. Following the developers’ guidance, no overall score was calculated; per-criterion ratings for each study are reported in the Supplementary Materials (Table S3).

To assess the reproducibility of the classification and appraisal scheme, a stratified random sample of 35 studies (32% of the review, spanning all ten application categories) was independently duplicate-coded by a second reviewer, who was blind to the first author’s codes, working from the same extracted purpose, features, and study design text. Agreement was quantified using percentage agreement and Cohen’s κ for each coded variable. Because MMAT Category 4 (“quantitative descriptive”) criteria assess general methodological rigor (sampling strategy, sample representativeness, measurement appropriateness, nonresponse bias, and statistical analysis) rather than measurement-specific properties such as criterion validity, systematic bias, or limits of agreement, and 82 of the 111 studies (74%) were coded in this category, MMAT ratings should be interpreted as a general methodological screen rather than a complete appraisal of metrological quality for the validation and reliability studies that dominate this review. For studies evaluating measurement apps against a criterion instrument, relative validity, absolute agreement, and reliability were additionally assessed (see Section 3.4, Section 3.5, Section 3.6, Section 3.7 and Section 3.8). Where relevant, methodological features such as the use of a reference standard, sample size, and study design are also noted in the synthesis to contextualize the reported findings.

## 3. Results

### 3.1. Overview of Included Studies

A total of 111 studies met the inclusion criteria. The screening and selection process, including reasons for exclusion at the full-text stage, is summarized in Figure 1.

**Figure 2:**
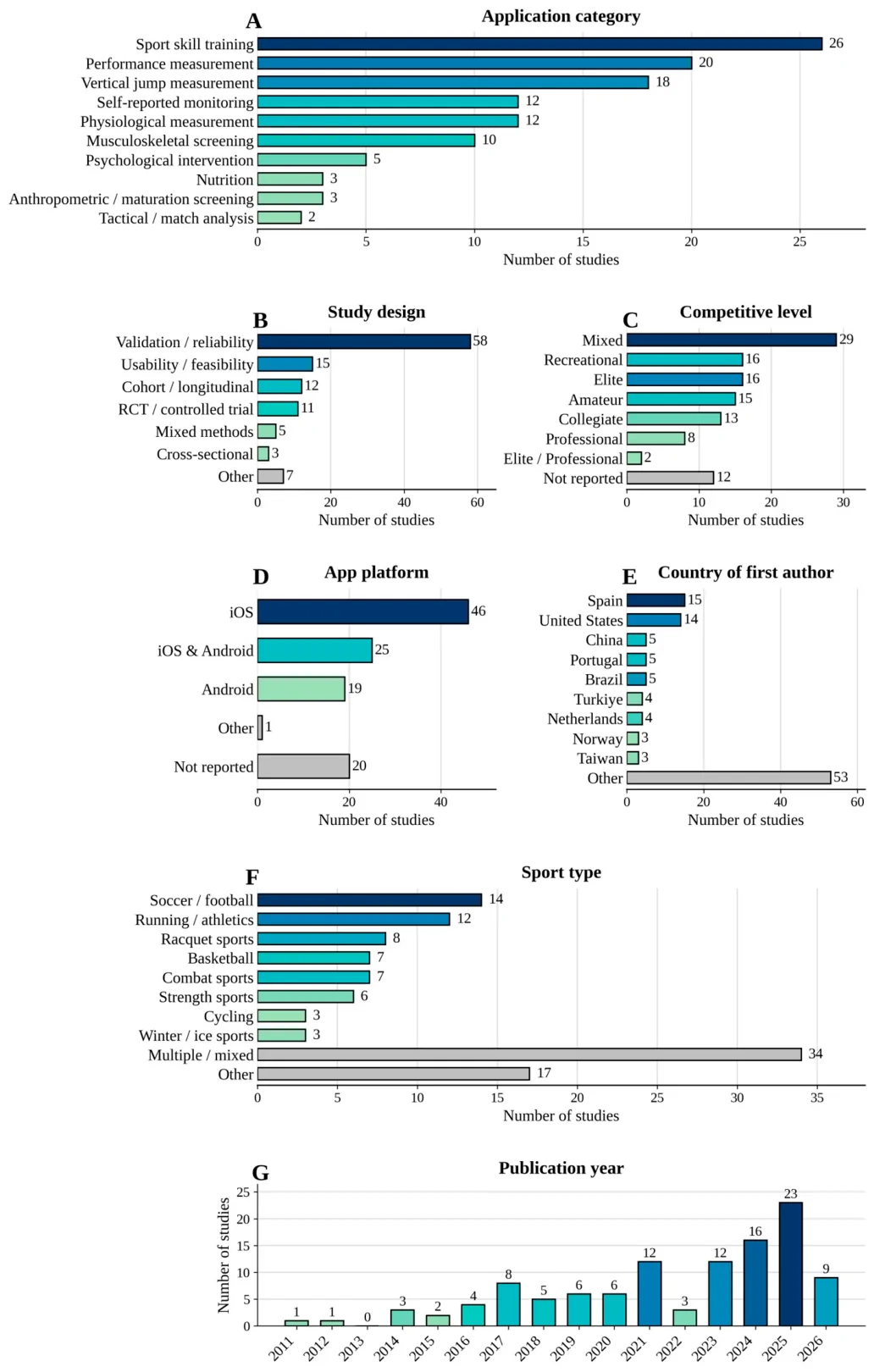
Overview of the 111 included studies. (**A**) Distribution of studies across the ten application categories; (**B**) study design; (**C**) competitive level; (**D**) app platform; (**E**) country of first author; (**F**) sport type; (**G**) publication year.

The inductive classification yielded ten application categories. The largest group of mHealth app studies was performance studies. Because vertical jump performance has more studies than other performance studies, the vertical jump measurement category (*n* = 18) was separated from the general performance measurement category (*n* = 20). This separation reflects more than study volume: vertical jump measurement apps are evaluated against a single, well-standardized criterion methodology (flight-time integration from a force platform or contact mat) and are dominated by a narrow set of purpose-built apps, predominantly the My Jump family (Section 3.5), giving the category a coherence in outcome, instrumentation, and evidence base that the more heterogeneous general performance measurement category, spanning sprint timing, barbell velocity, and sport-specific tools against varied criterion instruments, does not share. Ordered by number of studies, these were sport skill training (*n* = 26), performance measurement (*n* = 20), vertical jump measurement (*n* = 18), self-reported monitoring (*n* = 12), physiological measurement (*n* = 12), musculoskeletal screening (*n* = 10), psychological intervention (*n* = 5), nutrition (*n* = 3), anthropometric and maturation screening (*n* = 3), and tactical and match analysis (*n* = 2). Category definitions and the most-studied apps are presented in Table 1, and the distribution of studies across categories is shown in Figure 2A. The characteristics and evidence base of the included studies, organized by the ten application categories, are summarized in Table 2. A full study-by-study listing of all 111 included studies, including country, app, platform, sport, competitive level, study design, sample size, and primary outcome(s), is provided in the Supplementary Materials (Table S2). An overview of the included studies by application category, study design, competitive level, app platform, first-author country, sport type, and publication year is presented in Figure 2. Unless otherwise noted, all counts in this review refer to studies rather than to unique apps, app families, or app versions, since a single app may be evaluated across multiple studies and versions (Table 1).

**Figure 3:**
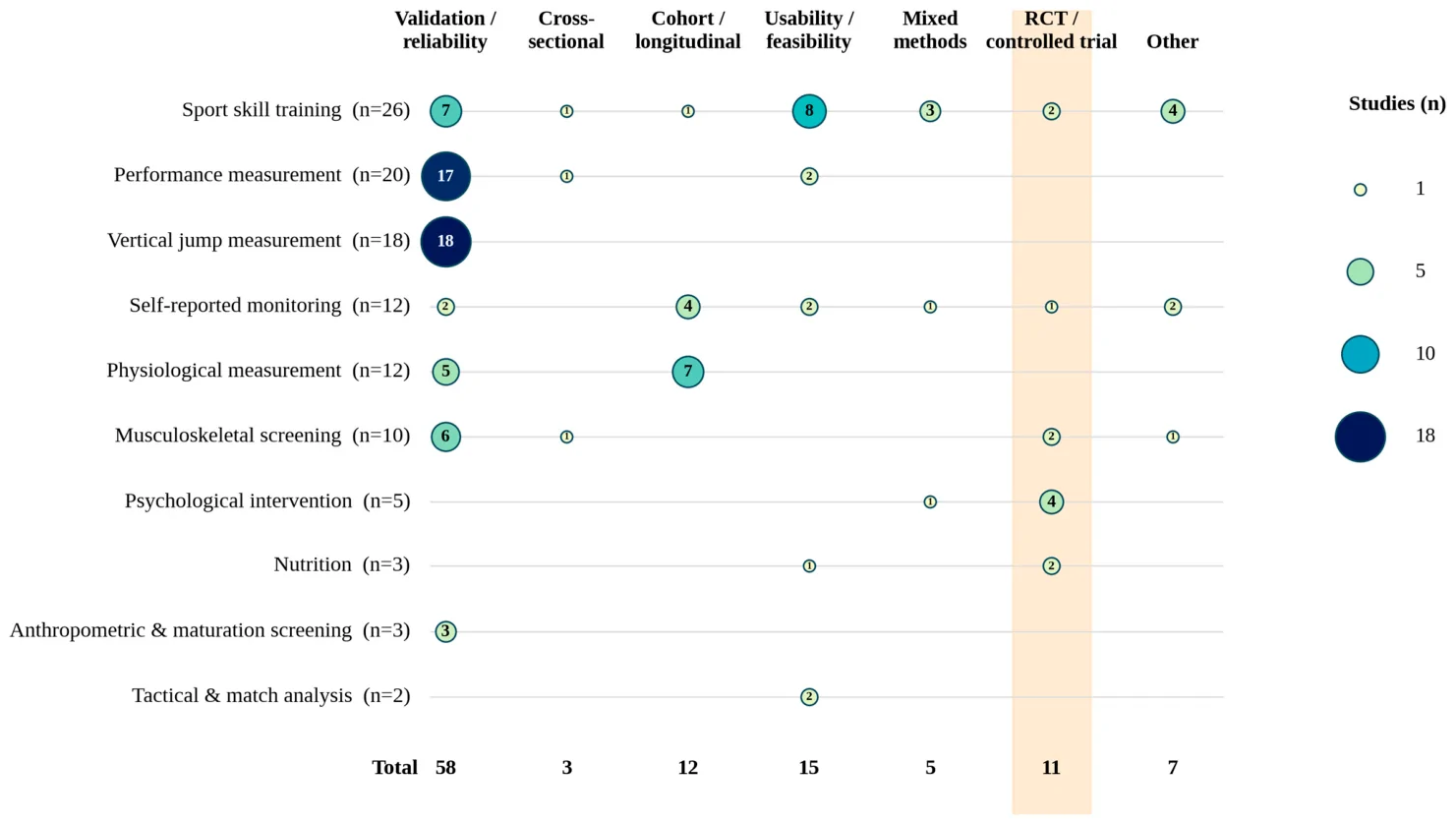
Evidence gap map of mobile health (mHealth) apps in sport training. Rows are the ten application categories (with the number of studies per category in parentheses); columns are study design types. Bubble size and shade are both proportional to the number of studies, and column totals are shown along the bottom. The shaded column highlights randomized controlled and other controlled trials, indicating the scarcity of effectiveness evidence relative to validation studies.

The ten categories differed systematically in scale, maturity, and evidence type (Figure 3). The three largest, sport skill training, performance measurement, and vertical jump measurement, together accounted for 58% of the evidence base (*n* = 64). The two measurement categories among them were the most metrologically developed: 17 of the 20 performance measurement studies and all 18 vertical jump measurement studies were validated against a laboratory criterion standard, reflecting a subfield that has prioritized establishing accuracy against such references. Sport skill training was more methodologically heterogeneous, spanning validation (*n* = 7), usability and feasibility (*n* = 8), controlled trials (*n* = 2), mixed methods (*n* = 3), and other designs (*n* = 6), consistent with apps intended to deliver coaching rather than quantify a single variable. Longitudinal evidence was concentrated in two categories, physiological measurement (5 of 12 studies) and self-reported monitoring (4 of 12), which tracked athletes across a training phase or competitive season rather than at a single time point. The four smallest categories (i.e., psychological intervention, nutrition, anthropometric and maturation screening, and tactical and match analysis) comprised 12% of studies (*n* = 13) and were the least mature. Notably, the two categories addressing behavior change rather than measurement were the only ones evaluated predominantly through controlled designs: 4 of 5 psychological intervention studies and 2 of 3 nutrition studies used randomized or controlled trials, whereas no study in either category used a validation design.

Independent of primary category, each app was also coded for secondary function (Measurement/Assessment, Feedback/Technique guidance, Intervention/Behavior change, and Decision-support), since apps commonly serve more than one role (Section 2.6; Table S6). Measurement was the dominant tag, present in 99 of 111 apps (89%), reflecting that nearly all apps in this review quantify some aspect of performance, physiology, or behavior, regardless of category. Feedback followed (45, 41%), Intervention (15, 14%), and Decision-support (11, 10%). Forty-eight apps (43%) carried more than one function, but this combination was strongly patterned by category maturity: the two most metrologically mature categories, vertical jump measurement and anthropometric and maturation screening, were almost entirely single-function measurement tools (2 of 18 and 0 of 3 multi-function, respectively), though the specific combination varied by category: nutrition apps combined feedback with intervention (3 of 3), while tactical and match analysis (2 of 2), sport skill training (18 of 26, 69%), and musculoskeletal screening (6 of 10, 60%) apps most often combined measurement with feedback, intervention, or decision-support. The full category-by-function matrix is provided in Table S6.

These category-level differences were reflected in the overall study characteristics (Figure 2). Publication output grew markedly across the review period, with more than half of all studies (*n* = 60, 54%) appearing since 2023 and a peak of 23 studies in 2025 (Figure 2G). The evidence base was dominated by validation and reliability designs (*n* = 58, 52%), with comparatively few randomized or controlled trials (*n* = 11, 10%) (Figure 2B). Most studies (*n* = 46) reported that apps were developed for the iOS platform, but 20 studies did not report the platform (Figure 2D). Athlete populations spanned all competitive levels, most frequently mixed-level samples (*n* = 29), and 12 studies did not report the competitive level (Figure 2C). Participant demographics were incompletely reported: age was specified in 77 of 111 studies, with reported means ranging from approximately 12 to 45 years and 19 studies conducted primarily in youth samples (mean age < 18 years); sex distribution was reported in 81 of 111 studies, comprising 29 male-only, 11 female-only, and 41 mixed-sex samples, with the remainder not specifying sex. Research was geographically concentrated, with Spain (*n* = 15) and the United States (*n* = 14) contributing the most first authors (Figure 2E). Studies most often recruited across multiple sports (*n* = 34); among single-sport studies, soccer or football (*n* = 14) and running or athletics (*n* = 12) predominated (Figure 2F).

The remainder of the Section 3 is organized as follows. Section 3.2 characterizes the technical attributes of the apps, namely their sensing requirements, computational approach, and processing location, across the full set of studies. Section 3.3, Section 3.4, Section 3.5, Section 3.6, Section 3.7, Section 3.8, Section 3.9, Section 3.10, Section 3.11 and Section 3.12 then examine each of the ten application categories in turn, following a common structure: what the apps measured or delivered, which apps and populations were studied, and what the evidence established. Section 3.13 synthesizes the findings that cut across all ten categories.

### 3.2. Technical Characteristics of the Included Apps

The technical characteristics of the included apps are summarized in Table 3. Sensing relied overwhelmingly on the smartphone’s own built-in sensors, requiring no dedicated external hardware: 69 studies (62.2%) evaluated apps using built-in smartphone sensors only, and a further 20 studies (18.0%) evaluated apps requiring no sensing at all, collecting data solely through manual entry. Only 22 studies (19.8%) required external hardware. The camera dominated, serving as the sensing modality in 49 studies (44.1%) for video capture and in five more for photoplethysmography, followed by built-in inertial sensing (inertial measurement unit or accelerometer, *n* = 15) and GPS or GNSS (*n* = 6); a full modality listing is provided in Table S4. Requirements were strongly patterned by category (Table 3): every vertical jump measurement study (18 of 18) and 18 of 20 performance measurement studies evaluated apps needing nothing beyond the phone, whereas physiological measurement was the sole inversion, with 9 of 12 studies requiring an external chest strap or optical finger sensor [17,33,34], and self-reported monitoring the opposite pole, with 9 of 12 studies having no sensing component at all.

The computational approach was correspondingly modest. Two thirds of studies (73 of 111, 65.8%) evaluated apps whose primary analytic step was either manual digitization, in which the user selects the take-off and landing frames and the app performs arithmetic only (*n* = 21, 18.9%), or deterministic rule-based computation (*n* = 52, 46.8%). Only 13 studies (11.7%) evaluated an app with a disclosed algorithmic method: named pose-estimation models (*n* = 8) [21,35], named machine learning or pattern recognition methods (*n* = 4) [36,37], and a single app embedding a large language model over rule-based stroke scoring [38]. No study evaluated an app with agentic capability. Ten of these 13 fell in sport skill training, while two categories showed no methodological middle ground at all: vertical jump measurement comprised only manual digitization (*n* = 12) and undisclosed automation (*n* = 6), and all three anthropometric and maturation screening studies evaluated apps whose method was never reported.

Twenty-five studies (22.5%) evaluated apps that performed an automated computation that the source study never described, leaving the underlying method unidentifiable from the report. These comprised both proprietary commercial tools (My Jump Lab AI mode [v4.5.9], SwingVision [v9.8.3], Maturo, MeThreeSixty, TargetScan, Fitnessmeter [v3.3]) and custom research apps that invoked machine learning without specifying an architecture. Reported terminology and disclosed method were largely unrelated (see Section 4.6: of the 23 studies (20.7%) describing their app as artificial intelligence, machine learning, or deep learning, a majority (*n* = 13) disclosed no algorithm, while 12 of the 22 studies performing undisclosed automated processing were never described as artificial intelligence at all. The label therefore neither implies a disclosed method nor tracks the presence of automation. This compounds the version-specific validity observed across automated and AI modes elsewhere in this review [39,40,41], because an undocumented method cannot be independently verified when it changes between releases.

Processing location was the least reported attribute of all: 83 studies (74.8%) provided no information on whether analysis occurred on the device or on a remote server, and this distribution should be read as evidence of under-reporting rather than as an architectural profile of the field.

The intended operator, whether the app is designed to be used by the athlete independently or administered by a coach or practitioner, also varied systematically by category. Video-based measurement apps were predominantly practitioner-administered: nearly all vertical jump measurement and performance measurement studies required a second person to operate the camera during testing, consistent with standard field-testing protocols for these tools. By contrast, self-reported monitoring, physiological measurement, psychological intervention, and nutrition apps were designed for athlete self-operation, reflecting their reliance on self-report or self-administered physiological signals. Overall, 55 of 111 studies (50%) evaluated athlete self-operated apps, 49 (44%) evaluated coach- or practitioner-operated apps, and 7 (6%) evaluated apps designed for both. Full per-study coding is provided in Table S4.

### 3.3. Sport Skill Training Category

Sport skill training was the largest category, comprising 26 studies across 16 sports, most frequently running (*n* = 6) and table tennis (*n* = 3). Most apps were custom research prototypes rather than established commercial products, following three approaches: camera-based video and pose-estimation analysis, motion classification from inertial sensors, and coaching-content or training-plan delivery. Reflecting this early developmental stage, usability and feasibility studies (*n* = 8) and validation studies (*n* = 7) predominated; only two controlled trials were identified, and 15 studies enrolled 14 or fewer participants.

Validated camera-based systems showed good to excellent agreement with reference standards, including SwingVision against radar and video analysis for tennis strokes (ICC = 0.76–0.97) [42], a markerless gait app against Vicon motion capture (ICC = 0.751–0.981) [20], and My Jump 2 for timing karate techniques (ICC = 0.944–0.998) [43]. Inertial sensor systems classified strokes, gears, and errors with approximately 70–100% accuracy [37,44,45], although smartphone sensors alone performed near chance in one study [36]. Evidence for effectiveness was limited but consistently positive: augmented-reality feedback improved badminton accuracy and technique in a six-week RCT (d = 0.85–1.12) [46], a six-month program improved coordination in youth basketball (partial η^2^ = 0.41–0.73) [47], and brief sound-intensity feedback reduced running impact loading by 28–36% [48].

The remainder of the category comprised early-stage work that illustrates both the breadth and the immaturity of the field. Coaching-delivery apps were evaluated mainly for usability and engagement, including real-time running-cadence feedback [49,50], adaptive running-training schemes [51], and a platform on which users completed substantially more trainer-created than self-created workouts [52]. Feedback prototypes targeted skills not otherwise observable by the athlete, such as foot-pressure visualization and trick recognition in skateboarding [53,54], lane-drift alerts enabling independent training for visually impaired swimmers [55], head-movement biofeedback during the golf swing [56], and parallel-turn scoring in alpine skiing [57]. Others demonstrated technical feasibility in single-case or small samples, including markerless analysis of gymnastics giant swings [35] and AI-generated cue-sport coaching rated highly usable [38]. Only two further studies tested learning outcomes: an Android volleyball skill-test instrument [58] and a non-randomized controlled trial of coordination games in youth basketball [47]. Overall, the predominance of small proof-of-concept studies and the scarcity of controlled trials indicate that most skill training apps remain at an early stage of validation.

### 3.4. Performance Measurement Category

**Figure 4:**
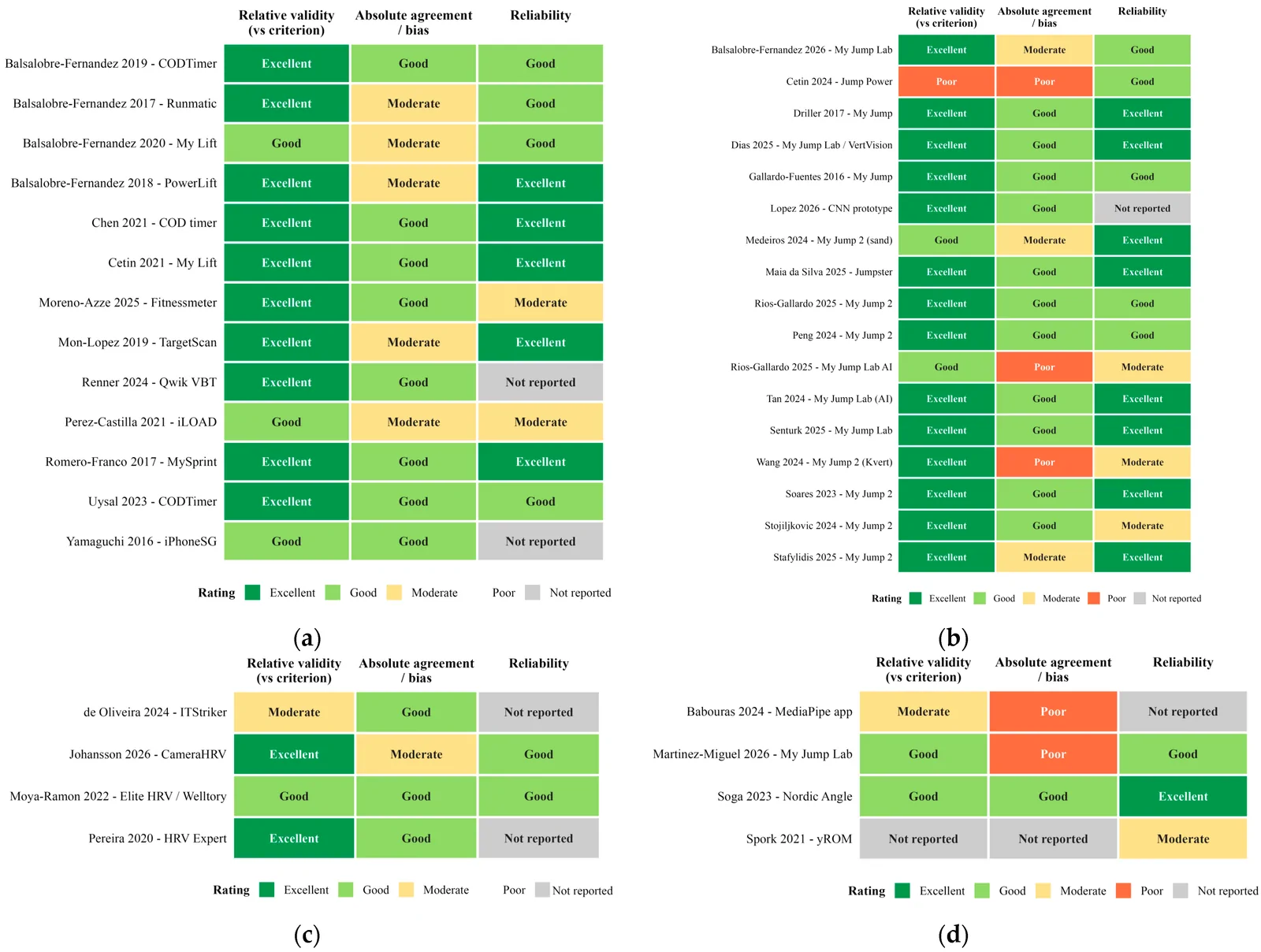
(**a**) Validity and reliability of performance measurement apps. Each study (row) is rated on three dimensions: relative validity against a criterion instrument, absolute agreement (systematic bias), and reliability. Ratings were coded from each study’s reported statistics as Excellent, Good, Moderate, Poor, or Not reported (see legend). Only studies comparing an app against a criterion or reference instrument are included. Studies shown: Balsalobre-Fernández 2019 [12], Balsalobre-Fernández 2017 [59], Balsalobre-Fernández 2020 [60], Balsalobre-Fernández 2018 [13], Chen 2021 [61], Cetin 2021 [62], Moreno-Azze 2025 [63], Mon-López 2019 [64], Renner 2024 [39], Pérez-Castilla 2021 [65], Romero-Franco 2017 [15], Uysal 2023 [66], Yamaguchi 2016 [67]. (**b**) Validity and reliability of vertical jump measurement apps. Dimensions, rating scale, and inclusion criteria as in Figure 4a. Studies shown: Balsalobre-Fernández 2026 [68], Çetin 2024 [69], Driller 2017 [14], Dias 2025 [70], Gallardo-Fuentes 2016 [71], Lopez 2026 [72], Medeiros 2024 [73], Maia da Silva 2025 [74], Ríos-Gallardo 2025 [40], Peng 2024 [75], Ríos-Gallardo 2025 [76], Tan 2024 [41], Şentürk 2025 [77], Wang 2024 [78], Soares 2023 [79], Stojiljković 2024 [80], Stafylidis 2025 [81]. (**c**) Validity and reliability of physiological measurement apps. Dimensions, rating scale, and inclusion criteria as in Figure 4a. Studies shown: de Oliveira 2024 [82], Johansson 2026 [83], Moya-Ramón 2022 [17], Pereira 2020 [34]. (**d**) Validity and reliability of musculoskeletal screening apps. Dimensions, rating scale, and inclusion criteria as in Figure 4a. Studies shown: Babouras 2024 [21], Martínez-Miguel 2026 [84], Soga 2023 [85], Spork 2021 [86].

Performance measurement apps quantified athletes’ physical or perceptual–cognitive capacities and comprised three functional groups: sprint and change-of-direction (COD) timing from high-speed video (MySprint, CODTimer, Fitnessmeter, Seconds Count); barbell velocity and one-repetition-maximum (1RM) estimation for velocity-based resistance training (PowerLift/My Lift, iLOAD, Qwik VBT, Metric VBT, and the smartwatch-based StrengthControl); and sport-specific measurement tools, including shooting-target scoring (TargetScan), baseball pitch speed, boxing punch detection, badminton footwork, running mechanics from video or acoustics (Runmatic, SoundTrack), core stability, and sensorimotor reaction testing. In contrast to skill training apps, most were named, purpose-built measurement apps evaluated in validation designs against criterion instruments (*n* = 17), timing gates, linear transducers, three-dimensional motion capture, radar, or official scoring systems, in trained or competitive populations, with samples of 2 to 62 participants. The relative validity, absolute agreement, and reliability ratings for each of these studies are summarized in Figure 4a.

Video-based timing apps approached criterion-level accuracy: MySprint agreed almost perfectly with photocells for 40 m sprint splits (r = 0.989–0.999; ICC = 1.00) and with radar for sprint mechanical outputs (bias < 2.5%) [15], and COD timing apps showed comparable validity (r = 0.96–0.99; ICC = 0.92–1.00) with clear superiority over handheld stopwatches [12,61,63,66]. Barbell velocity apps were generally valid and reliable, including for 1RM estimation (ICC = 0.99) [62], but showed systematic biases precluding interchangeable use with linear transducers [13,65], and validity was highly app-dependent: in a head-to-head comparison, Qwik VBT matched a linear transducer (RMSE = 0.01–0.04 m·s^−1^), whereas MyLift failed to record 84% of bench-press repetitions [39], and smartwatch-based 1RM prediction succeeded in only 8.9% of attempts [87]. Sport-specific tools were similarly accurate, including shooting-score analysis (ICC ≥ 0.998) [64], acoustic cadence estimation (error 1.6%) [88], and reliable app-based sensorimotor and spatial-perception tests (test–retest r = 0.73–0.87; α = 0.96) [89,90].

A smaller group of studies established feasibility rather than criterion validity: a smartphone accelerometer discriminated fast from slow badminton footwork across most movement directions (Cohen’s d = 0.75–1.70) [91], a boxing app detected punches with approximately 95% accuracy and was rated highly usable [92], and app-measured single-leg deadlift stability correlated with 10 m sprint velocity in elite youth soccer players (r = 0.765) but not with change-of-direction speed [93]. Overall, high-speed-video measurement apps can substitute for laboratory timing systems in field settings, whereas sensor- and watch-based tools remain more variable, and findings are tied to specific apps and software versions.

### 3.5. Vertical Jump Measurement Category

Vertical jump measurement was the most homogeneous category, dominated by the My Jump family (My Jump, My Jump 2, My Jump Lab), which featured in 15 of the 18 studies; the remainder evaluated the face-detection-based Jump Power app, the sensor-based Jumpster, the video-based VertVision, and a novel audio-based prototype. All studies were validation or reliability designs against criterion instruments (force platforms, contact mats, or photoelectric systems) and collectively extended the evidence across diverse populations, from youth and recreational athletes to elite, national, and Olympic-level competitors, and beyond simple jump height to squat and drop jumps, unilateral jumps and interlimb asymmetry, jumps on sand, vertical stiffness, and the dynamic rebound index. The relative validity, absolute agreement, and reliability ratings for each of these studies are summarized in Figure 4b.

Video-based flight-time measurement at 240 fps with manual frame selection consistently approached criterion-level accuracy, with correlations and ICCs typically 0.94–0.999 and mean biases of roughly 0.2–1 cm across jump types, sports, and surfaces [14,71,73,81], although derived metrics were less robust—peak power showed only moderate agreement [73] and vertical stiffness was systematically underestimated with proportional error [78]. Evidence for newer automated modes was mixed: real-time automatic detection in My Jump Lab remained valid (ICC = 0.984, bias ≈ −1 cm) [77], and the AI mode agreed closely with force platforms in one large study of 88 national-level athletes (ICC = 0.973, no systematic bias) [41], but the 30 fps AI bounding-box mode underestimated jump height by ≈3.2 cm with proportional bias in another [40]. Among alternative approaches, the free sensor-based Jumpster correlated strongly with a force platform (r = 0.91) [74] and an audio-based prototype achieved a flight-time error of 7 ms [72], whereas Jump Power was reliable (CV < 5%) but not valid, overestimating jump height by ≈9 cm [69].

The My Jump family’s validity extended consistently across sports and jump types: My Jump 2 agreed almost perfectly with force platforms for arm-swing countermovement jumps in professional fencers, swimmers, and divers (ICC = 0.998–0.999) [75] and with a criterion system in judo players (r = 0.97; ICC = 0.94–0.97) [79], and both My Jump Lab and VertVision showed very high concurrent validity against a contact mat in handball players (ICC = 0.993–0.998), though both overestimated jump height by 1.86% [70]. Interlimb asymmetry, a derived metric of growing interest, was also captured validly, with My Jump 2 matching criterion measures for drop-jump asymmetry in young female basketball players (ICC > 0.9), although test–retest reliability was substantially weaker for contact time than for jump height (ICC = 0.46 vs. 0.88) [80]. Overall, high-frame-rate flight-time apps can substitute for laboratory systems in field-based jump monitoring, whereas automated, AI, and alternative sensing modes require app- and version-specific validation before interchangeable use.

### 3.6. Self-Reported Monitoring Category

Self-reported monitoring apps captured athletes’ subjective wellness, training load, and health status rather than physical performance, and comprised three functional groups: daily wellness and session RPE logging platforms (PMSys, TrainingPeaks, SMARTABASE, Titan Athlete, SaluTrack); menstrual-cycle tracking tools for female athletes (Coral and custom calendar-based diaries); and broader athlete-management or health-monitoring systems integrating questionnaires with physiological or wearable data (Optio, Readiness Advisor, and a custom integrated-EHR system). Unlike the preceding categories, few studies were device-validation designs; most were prospective cohort, feasibility, or mixed-method studies (with a single RCT and two construct-validity studies) conducted predominantly in elite and collegiate populations, most frequently in women’s soccer and other female athlete contexts, with samples of 9 to 400 participants.

The evidence centered on three questions. First, whether self-reported wellness predicts training load or performance: session RPE captured via app showed acceptable construct validity against heart-rate-based load measures (r = 0.51–0.83) [94], and poorer pre-training wellness, particularly the mood component, predicted higher post-session RPE in collegiate soccer [95], whereas subjective wellness did not meaningfully predict individual match performance in elite women’s football [96]. Second, how subjective ratings relate to objective markers: a year-long study of elite endurance athletes found moderate-to-strong associations between subjective ratings and wearable/validated counterparts (r = 0.39–0.81) that were stronger at the individual than group level, while notably heart-rate variability did not correlate with subjective stress [97]. Third, feasibility and implementation: menstrual-cycle and health-monitoring apps achieved high adherence (76–91%) and acceptable usability [98,99,100], but qualitative and controlled work showed that sustained engagement depends on clarity of purpose, individualized feedback, and stakeholder buy-in rather than the tool itself; an educational intervention failed to improve adherence in an RCT, with 52% dropout [101,102]. Exploratory machine learning work further suggested that team-level data improved individual readiness prediction [103].

Two further studies described prototype systems evaluated at the development stage rather than against athlete outcomes: a remote two-component monitoring system combining physical and psycho-emotional questionnaires in 400 student-athletes, in which monitored athletes showed lower stress severity and greater stress tolerance than controls [104], and a machine learning trainer integrating exercise, diet, and stress management for badminton athletes, whose stress classifier reached an AUC of 0.72 but which reported no participant-level athletic or behavioral outcomes [105]. Overall, self-reported monitoring apps are feasible and provide practically useful team- and individual-level information, but their value lies in informing load management and communication rather than in predicting performance, and effective implementation is a greater challenge than measurement itself.

### 3.7. Physiological Measurement Category

Physiological measurement apps quantified athletes’ internal responses to training and were dominated by heart-rate variability (HRV) monitoring, which featured in 10 of the 12 studies, most commonly ithlete (evaluated in five studies) and HRV4Training (three); the remaining two evaluated a boxing-specific aerobic-testing app (ITStriker) and a telemetric system for detecting cardiac disorders during cycling. The apps acquired their signal via an optical finger sensor, the smartphone camera or flash (photoplethysmography), or a paired Bluetooth chest strap, and were studied predominantly in collegiate, elite, and professional endurance and team-sport athletes (samples of 7 to 63). They followed three designs: concurrent validation against a criterion standard (*n* = 6), longitudinal monitoring of autonomic responses to training load (*n* = 5), and one cross-sectional study. The relative validity, absolute agreement, and reliability ratings for the concurrent validation studies are summarized in Figure 4c.

Validation supported the accuracy of app-based HRV when the signal source was adequate. Chest-strap-paired apps agreed near-perfectly with electrocardiography (ECG) for time-domain indices, including for ultra-short 1 min recordings (r = 0.72–0.94) [17,34], whereas camera-based photoplethysmography was valid but less precise, showing substantially greater measurement error than a chest strap (RMSSD MAPE 17.5% vs. 2.2%) [83]. Among the non-HRV apps, ITStriker validly estimated heart-rate indices against an incremental treadmill test (r = 0.73–0.85) [82], and the telemetric system detected arrhythmias and hypoxemia during field efforts that pre-participation screening had missed [106]. The longitudinal studies showed that app-derived HRV is sensitive to training load, with vagal markers declining during overload and recovering during taper across several sports [33,107,108], and season-long monitoring revealing progressive autonomic suppression in American-football linemen that preceded injury [109]. Cross-sectionally, time-domain HRV correlated with aerobic performance in youth futsal players (r = 0.65–0.78) [110]. Two caveats recurred: individual HRV responses were highly heterogeneous, cautioning against autoregulation from group-derived thresholds, and long-term compliance was difficult to sustain, dropping below 45% in one team and depending heavily on coach engagement [111]. Overall, smartphone HRV apps are valid against ECG, most accurately with a chest strap, and sensitive to training-induced autonomic change, but their practical value is constrained by inter-individual variability and adherence challenges.

### 3.8. Musculoskeletal Screening Category

Musculoskeletal screening apps assessed injury risk factors, screened for injury, or delivered injury-prevention programs, and were the most functionally diverse category. They comprised measurement apps quantifying a biomechanical risk factor, namely markerless pose-estimation tools for ACL-related jump kinematics [21,112], video- or sensor-based goniometers for hamstring break-point angle [84,85] and hip range of motion [86]; a multi-test functional screening battery combining functional tests and joint range of motion [113]; a single concussion-diagnosis app using smartphone pupillometry [114]; and video-guided neuromuscular injury-prevention programs, most prominently the free “Strengthen your Ankle” app [24,115]. Populations spanned collegiate, professional, and recreational athletes across soccer, football, swimming, and general sport, with samples ranging from 7 participants to a 25,781-user implementation dataset. The relative validity, absolute agreement, and reliability ratings for the measurement apps that were compared against a criterion instrument are summarized in Figure 4d.

The measurement apps showed a recurring pattern of good relative validity but limited absolute accuracy: pose estimation agreed with reference systems in the frontal plane but underestimated sagittal-plane knee angles [21], and My Jump Lab hamstring torque correlated strongly with a criterion dynamometer (r = 0.77) yet systematically overestimated it, warranting only group-level screening [84]; the concussion app differentiated concussed from baseline athletes with 91% accuracy in a small pilot [114]. In a cross-sectional application rather than a validation study, app-based functional screening of CrossFit practitioners identified poor shoulder medial rotation and ankle dorsiflexion range of motion in the majority of participants despite relatively low reported injury prevalence, illustrating the use of these tools for risk profiling rather than diagnosis [113]. For prevention delivery, a randomized controlled trial found the ankle-sprain app as effective and cost-effective as an equivalent printed booklet at 12 months [115], but a large implementation study of the same app reported low reach (2.6%) and poor adherence [24], and app-based exercise instruction elicited lower protective muscle activation than in-person instruction [116]. Overall, these apps can make risk assessment and prevention programs more accessible and are acceptably valid for relative or group-level screening, but limited absolute accuracy and poor real-world adherence constrain their standalone use for individual clinical decisions.

### 3.9. Psychological Intervention Category

Psychological intervention apps delivered mental skills or mental health support rather than physical measurement, and each of the five studies evaluated a different app targeting a different psychological construct: a rational–emotive behavior therapy (REBT) micro-learning program delivered via the TalentCards platform to challenge irrational performance beliefs [117], a cognitive–behavioral anxiety-management app (WorryTree) [118], a slow-paced breathing pacer (BreathPacer) [22], the commercial game Fruit Ninja repurposed as domain-generic executive-function training [119], and a custom self-regulation app (REMBO) providing “traffic-light” training load advice to prevent running-related injury and fatigue [120]. All five were effectiveness studies, comprising three randomized controlled trials, one randomized comparative trial, and one mixed-method pre–post design, conducted across gymnastics, martial arts, soccer, running, and mixed-sport populations, predominantly in small samples ranging from 8 to 425 participants.

The effectiveness evidence was mixed and closely tied to whether the intervention targeted a psychological outcome directly. The two apps addressing anxiety and performance beliefs showed benefits: the WorryTree app produced large reductions in anxiety and improvements in general mental health over six months (*p* < 0.001) [118], and the REBT program significantly reduced irrational performance beliefs relative to an active control (d = 0.96), although it did not change coping or mental health [117]. Evidence for the indirect and repurposed interventions was weaker: the breathing app improved subjective well-being and distress in a pre–post pilot but, notably, resting heart-rate variability declined rather than improved, and only five of eight participants returned adherence diaries [22]; the Fruit Ninja training produced no executive-function benefit, with the only significant interaction favoring the control group [119]; and the REMBO self-regulation app showed no effect on running-related injuries or chronic fatigue by any analysis, with only 40% of participants using it even once [120]. Overall, app-delivered psychological interventions that directly target a mental health or belief outcome show early promise, whereas indirect or repurposed approaches have not demonstrated benefit, and low adherence again emerged as a recurring constraint on real-world effectiveness.

### 3.10. Nutrition Category

Nutrition apps supported dietary education, monitoring, and behavior change in athletes, and comprised three studies of distinct tools: the widely used food-tracking app MyFitnessPal delivered alongside nutrition-education modules [121], the image-based dietary-logging app MealLogger with in-app dietitian feedback and social features [23], and a custom AI-driven app, NutriFit-AI, providing computer vision food logging and personalized meal plans [122]. All three were effectiveness studies, comprising two randomized controlled trials and one single-arm usability pilot, conducted in student, adolescent, and elite athlete populations with samples of 17 to 152 participants.

All three reported improvements in nutrition knowledge or dietary behavior. The MyFitnessPal-based intervention improved sport-nutrition knowledge and dietary habits significantly more than traditional lecture-based education over three months (*p* < 0.001) [121], and the MealLogger pilot in elite field-hockey players produced a moderate gain in nutrition knowledge (54.7% to 61.1%, *p* = 0.01) with high acceptability; all participants preferred it to paper records, and openness to dietitian advice rose from 0% to 82% [23]. The AI-based NutriFit-AI reported the largest effects, with higher dietary adherence (89% vs. 58–67%) and greater fitness and nutrient-intake gains than both traditional education and MyFitnessPal [122]. Overall, nutrition apps appear feasible and effective for improving athletes’ dietary knowledge and self-reported behavior, but the evidence base is very small, short in duration, and reliant on self-reported outcomes rather than objective dietary or performance measures, and the strongest reported effects come from the study with the most serious reporting concerns.

### 3.11. Anthropometric and Maturation Screening Category

Anthropometric and maturation screening apps estimated body composition or biological maturity from smartphone images, and comprised two distinct tools. The first, MeThreeSixty, derived whole-body and appendicular body composition from two 2D smartphone photographs, validated against dual-energy X-ray absorptiometry (DXA) in collegiate baseball players [123]. The other two studies both evaluated Maturo, an AI-based app estimating biological age, predicted adult height, and peak height velocity (PHV) from camera-based somatic measurements, validated against expert assessment using the Khamis-Roche and Mirwald reference equations in youth volleyball, basketball, and football players [124,125]. All three were cross-sectional validation studies with small samples (*n* = 41–103), and the two maturation studies were conducted by authors affiliated with the app under evaluation.

The maturation app demonstrated strong validity for most outcome measures, but showed a consistent weak point in estimating the timing of the growth spurt. Maturo agreed excellently with expert-derived estimates of biological age, predicted adult height, and maturity classification (ICCs 0.94–0.99; Cohen’s κ = 0.82–0.95) across both studies, but PHV age—the timing of the growth spurt—showed only moderate agreement (ICC = 0.673 in both), with a systematic tendency to underestimate biological age that the developers flagged for algorithm refinement [124,125]. The body composition app performed less well: MeThreeSixty’s existing manufacturer equations reached no equivalence with DXA for any body composition variable (R^2^ = 0.00–0.68) and were especially poor for appendicular lean mass of the arms, and although newly developed population-specific equations reduced measurement error, they still did not achieve statistical equivalence in a seven-athlete test sample [123]. Overall, camera-based maturation screening shows promise as a non-invasive, low-cost alternative to expert assessment for most maturity indices, whereas smartphone-based body composition estimation requires population-specific calibration and cannot yet be considered interchangeable with DXA; the very small evidence base, and the developer affiliation of the maturation studies, warrant cautious interpretation.

### 3.12. Tactical and Match Analysis Category

Tactical and match analysis was the smallest category, comprising two custom research apps (SoniSailing and PoloTrac) for team- and individual-sport tactical support. SoniSailing delivered real-time GNSS-based auditory feedback through bone-conduction headphones to guide upwind tactical decisions in windsurfing [126], while PoloTrac enabled live event-tracking and generated advanced post-game statistics for water polo, including a logistic-regression model predicting per-shot goal probability [127]. Both were usability and feasibility studies in small samples (*n* = 13 and *n* = 5, respectively), with no criterion-standard performance comparison. SoniSailing was rated positively for its technical function, but its perceived usefulness was inversely related to competitive experience, with junior athletes finding the tactical cues significantly more helpful than senior national-team members [126], suggesting a target population of less experienced competitors. PoloTrac was perceived as intuitive and professional, and its embedded predictive model achieved 70% accuracy on a held-out test set of collegiate shots, although users occasionally entered data incorrectly during live tracking [127]. Overall, tactical analysis apps remain at an early proof-of-concept stage, demonstrating feasibility of real-time feedback and automated match statistics but lacking any evaluation of their effect on tactical performance or competitive outcomes.

### 3.13. Cross-Cutting Findings

Four patterns were synthesized from the included studies across the ten categories. The most pronounced was a structural imbalance in study design: the evidence base was dominated by validation and reliability studies (58 of 111) and contained few controlled effectiveness trials (11 of 111). This distribution of study designs across the ten application categories is displayed in the evidence gap map (Figure 3). This imbalance was uneven: the measurement categories were almost entirely validation work (every vertical jump study and most performance measurement studies), whereas effectiveness evidence was concentrated in the psychological, nutrition, skill training, and injury-prevention categories (e.g., [46,115,118,121]). For most apps, therefore, the literature shows that a measurement can be trusted, but not that acting on it changes training, performance, or injury risk.

A second pattern spanning the measurement categories was good relative validity but limited absolute accuracy: apps agreed well in rank order or correlation, yet showed systematic, often proportional bias that precluded interchangeable use, appearing in musculoskeletal screening [21,84], vertical jump [78], and body composition and maturation screening [123,124]. These tools were generally adequate for group-level monitoring but not for individual clinical or selection decisions. Third, and most consistent in real-world use, adherence rather than measurement accuracy determined whether an app delivered value: wherever apps were deployed longitudinally, engagement was the dominant constraint, with HRV compliance falling below 45% [111], an otherwise-validated ankle-sprain app reaching only 2.6% of its target population [24], and a self-regulation app used at least once by only 40% of participants [120].

Finally, the field exhibited several common indicators of limited maturity: predominantly small samples (36 of the 111 studies enrolled ≤15 participants), many custom prototypes, with app availability unreported for more than half of the apps, validity that was frequently app- and version-specific across automated and AI modes [39,40,41], and several of the most recently emerging application areas relying on developer-conducted evidence [122,124]. Taken together, these four patterns describe a field that has established measurement feasibility across a wide range of training functions but has not yet demonstrated that app-guided decisions improve athlete outcomes, that apps are adopted and sustained in practice, or that their accuracy holds under independent, large-scale evaluation.

## 4. Discussion

### 4.1. Summary of Principal Findings

This scoping review identified 111 studies evaluating mHealth apps in sport training and classified them into ten inductively derived application categories, spanning nearly every function of the training process from field-based performance testing to psychological support. To the best of our knowledge, this is the first review to map mHealth apps across the full breadth of sport training rather than within a single measurement domain, modality, or population. Whereas prior syntheses were confined to one performance domain [25], one technological modality [26], or to wearable devices rather than apps [28], the present review encompasses the full range of app-based tools used across the training process. The literature has grown rapidly, with more than half of the studies (*n* = 60, 54%) published since 2023. Despite this rapid growth, the field’s strengths and weaknesses follow a consistent pattern that is largely independent of the specific app or function under study.

Synthesizing across all 111 studies, four patterns recurred regardless of application category. First, the field is organized around measurement rather than outcomes: validation and reliability designs dominated the evidence base, whereas studies testing whether acting on an app changed an athlete’s outcome were rare (see Section 4.2). Second, among measurement apps, good relative validity coexisted with limited absolute accuracy, because systematic bias repeatedly prevented an app from being used interchangeably with its criterion instrument (see Section 4.3). Third, once apps were deployed over time rather than tested in a single session, adherence rather than accuracy determined whether they delivered value (see Section 4.4). Last, several features typical of a still-emerging field recurred across categories: small samples and developer-conducted evidence concentrated in the newest application areas (see Section 4.5). Many apps were academic prototypes rather than commercial products, so unreported availability and app-version-specific validity reflect the practical realities of turning a research tool into deployable software, a process that requires sustained investment and takes considerable time, rather than a flaw in the work itself. Together, these patterns show that mHealth apps have already demonstrated reliable measurement across a wide range of training-relevant variables; the next frontier for the field is to show that acting on these measurements improves athlete outcomes and to support the sustained adoption of validated tools in everyday practice.

### 4.2. A Field Organized Around Measurement, Not Outcomes

Of the 111 included studies, 58 (52%) employed validation or reliability designs, establishing whether an app measured a variable accurately. Only 11 (10%) used a randomized or controlled design to test whether acting on the app changed an athlete’s outcome. These two design types answer different questions, and their balance was strongly conditional on application category (Figure 3). The measurement-oriented categories were almost entirely validation work: all 18 vertical jump studies and 17 of the 20 performance measurement studies compared an app against a criterion instrument, reflecting a subfield that has prioritized establishing accuracy against laboratory reference standards. The categories oriented toward behavior change showed the inverse: four of five psychological intervention studies and two of three nutrition studies used randomized or controlled designs, while neither category contained a single validation study. For the majority of apps, therefore, the literature demonstrates that a variable can be measured accurately, but not that acting on that measurement changes training, performance, or injury risk.

This imbalance is intelligible in light of how the two groups of apps are constructed and evaluated. Measurement apps quantify a single physical variable, such as flight time, sprint split, barbell velocity, or heart-rate variability, for which an accepted criterion instrument already exists, so their evaluation reduces to a tractable and readily publishable question of agreement against that criterion. Behavior-change apps, by contrast, have no external gold standard against which a reading can be verified; their only meaningful test is whether they alter an outcome, which necessarily requires a controlled design. The observed pattern thus reflects the differing evaluative logic of the two app types as much as any deliberate research priority. It nonetheless mirrors a well-documented feature of the broader mHealth literature, which has been characterized as facing an “evaluation crisis” in which most apps reach users with little supporting evidence and rigorous effectiveness testing lags far behind development and validation [128].

The consequence is a broken pipeline from measurement to practice. Specifically, a successful conceptual pathway should start from technical validity, measurement reliability and responsiveness, decision rules, practitioner adoption (coaches and athletes) to better athlete outcomes. In sport science, the value of a monitoring tool lies not in the measurement itself but in the decision it informs, whether to adjust load, modify a technique, or intervene on injury risk. A valid measurement is therefore a necessary but not a sufficient condition for improving those decisions [129,130]. The present review shows that most sport-training apps have satisfied the necessary condition while leaving the sufficient one untested: it remains largely unknown whether athletes who are monitored, screened, or coached through an app achieve better outcomes than those who are not. This gap is most consequential precisely in the largest and most metrologically mature categories, where confidence in measurement accuracy risks creating a misleading impression that the tools’ practical utility has been established.

### 4.3. Relative Validity Without Absolute Accuracy

A second pattern, spanning every category in which apps were compared against a criterion instrument, was the coexistence of good relative validity with limited absolute accuracy. The apps were highly correlated and ranked measurements similarly, but systematic, often proportional, bias limited their interchangeability. Pose estimation demonstrated good agreement with the reference systems for the frontal plane measurements but underestimated sagittal-plane knee angles [21]. Similarly, My Jump Lab hamstring torque correlated strongly with a criterion dynamometer (r = 0.77) while systematically overestimating it [84]; vertical stiffness was underestimated with proportional error [78]; and MeThreeSixty reached no equivalence with DXA for any body composition variable [123].

This pattern is partly a methodological artifact arising from how validation studies are conducted and reported. Correlation coefficients quantify relative reliability—the extent to which individuals maintain their rank order between instruments—and can be high even when two instruments return substantially different values [131,132]. Because heteroscedastic error is the norm for ratio-scale variables in sport science [131], reporting relative indices alone systematically overstates a tool’s readiness for individual use. Validation studies should therefore report bias and limits of agreement, test formally for proportional bias, and interpret both against a predefined analytical goal rather than a correlation threshold [133].

The practical implication is that these tools should be matched to the decisions their accuracy can support. Good relative validity suffices for ranking athletes within a squad, profiling group-level risk, and tracking within-athlete change, provided the same app, device, and protocol are used throughout, since a stable bias cancels in repeated within-subject comparisons. It does not suffice for individual clinical or selection decisions, or for pooling app-derived and criterion-derived values. Validity also proved app-, mode-, and version-specific rather than generalizable to a class of technology: one barbell velocity app matched a linear transducer while another failed to record 84% of bench-press repetitions [39], and the AI mode of My Jump Lab agreed closely with force platforms in one study yet underestimated jump height by approximately 3.2 cm in another using a lower frame rate [40,41]. Accuracy, therefore, cannot be inferred for a differently versioned or configured tool, and studies should report the app version, operating system, and capture settings under which validity was established.

### 4.4. Adherence as the Rate-Limiting Step

The third pattern was the most consistent in real-world use. An app tested in a single session need only be accurate, but an app deployed longitudinally must also be used repeatedly over weeks or months. In every longitudinal deployment reviewed, it was this second requirement that proved decisive: engagement, rather than measurement accuracy, determined whether the tool delivered value. This was evident across otherwise unrelated tools. HRV monitoring compliance fell below 45% in one team and depended heavily on coach engagement [111]; a self-regulation app intended to prevent running-related injury was used at least once by only 40% of participants [120]; a breathing-intervention pilot recovered adherence diaries from five of eight participants [22]; and an educational intervention designed specifically to improve monitoring adherence failed in a randomized trial with 52% dropout [102]. This is not a peculiarity of sport. Attrition and non-use are recognized as characteristic features of eHealth applications rather than aberrations, to the extent that they have been argued to require their own science rather than being treated as study failures [134].

The most instructive case is the ankle-sprain prevention app, which was as effective and as cost-effective as an equivalent printed booklet at 12 months in a randomized controlled trial [115], yet reached only 2.6% of its target population with poor adherence when implemented at scale [24]. Efficacy and reach are therefore separable problems, and demonstrating the former does nothing to secure the latter. Evaluation frameworks developed for health promotion make this explicit by treating reach, adoption, implementation, and maintenance as dimensions of impact distinct from efficacy, and by noting that programs evaluated on efficacy alone can waste resources and stall between research and practice [135]. The present review found the sport-training app literature to be concentrated almost entirely on the efficacy dimension, with reach and maintenance reported incidentally if at all.

Where adherence was high, it was sustained by the coaching and communication structures around the app rather than by the app itself. Menstrual-cycle and health-monitoring apps achieved 76% to 91% adherence with acceptable usability [98,99,100], and qualitative work identified clarity of purpose, individualized feedback, and stakeholder buy-in as the conditions on which sustained engagement depended [101,102]. This suggests that the field’s next bottleneck is not sensor accuracy but implementation. Studies deploying apps longitudinally should report adherence, reach, and dropout as primary outcomes rather than as incidental observations, and should describe the coaching and communication structures within which the app was embedded, since these appear to determine whether an otherwise valid tool is used at all.

### 4.5. Indicators of an Immature Evidence Base

As the last of the four patterns synthesized from this review, several conventional markers of a young field recurred across the ten categories. Samples were predominantly small, with 36 of the 111 studies enrolling 15 or fewer participants, a constraint most acute in sport skill training, where 15 of its 26 studies enrolled 14 or fewer. Many of the apps evaluated were custom research prototypes rather than tools available to practitioners, and app availability went unreported for more than half of the apps identified. Reporting was incomplete on basic descriptors: platform was not reported in 20 studies and competitive level in 12. A subset of papers were system demonstrations rather than studies, lacking an explicit research question and reporting no statistical analysis, and replication was largely absent, with most apps evaluated exactly once. These features are consistent with a literature that has grown very rapidly, with more than half of all studies (*n* = 60, 54%) published since 2023, and that has prioritized demonstrating what is technically possible over establishing what is reliably true.

A related concern is the provenance of the evidence in the most recently emerging application areas. The two studies validating the maturation-screening app Maturo were conducted by authors affiliated with the app under evaluation [124,125], and the study reporting the largest effects in the nutrition category, for a developer’s own AI-based app, was also the study with the most serious reporting concerns [122]. Industry-sponsored drug and device studies are more likely than independently funded studies to report results and conclusions favorable to the sponsor, and to show weaker agreement between their results and the conclusions drawn from them [136]. The pattern in this review, in which developer involvement clusters in precisely the areas with the least independent corroboration, warrants cautious interpretation of those findings until they are replicated by unaffiliated groups.

Methodological quality also varied systematically by criterion rather than uniformly across studies. Among the 82 studies coded under MMAT Category 4, the dominant design bucket for validation and reliability studies, sample representativeness was the most frequently unmet criterion: 65 of 82 studies (79%) were rated “No,” compared with 4 rated “Yes” and 13 “Can’t tell.” Measurement appropriateness and statistical analysis were more often satisfied (74% and 72% rated “Yes,” respectively), while risk of nonresponse bias was rated “Can’t tell” in 68% of studies, reflecting incomplete reporting rather than a specific methodological failure. This pattern reinforces the small, non-representative samples noted above and indicates that the evidence base’s principal weakness lies in generalizability rather than in measurement or analytic execution. Full per-study, per-criterion ratings are reported in Table S3.

Finally, the evidence base is narrower than its breadth of application suggests. Research was geographically concentrated, with Spain (*n* = 15) and the United States (*n* = 14) contributing the most first authors, and sport coverage was uneven, with soccer or football (*n* = 14) and running or athletics (*n* = 12) predominating among single-sport studies. Several application areas rest on almost no evidence, with tactical and match analysis, nutrition, and anthropometric and maturation screening each comprising three or fewer studies. Combined with the app- and version-specificity described in Section 4.3, this means that published validity often cannot be transferred across populations, sports, or software releases, and that the apparent maturity of the field as a whole conceals considerable fragility within its individual categories.

### 4.6. Algorithmic Transparency and Reproducibility

**Figure 5:**
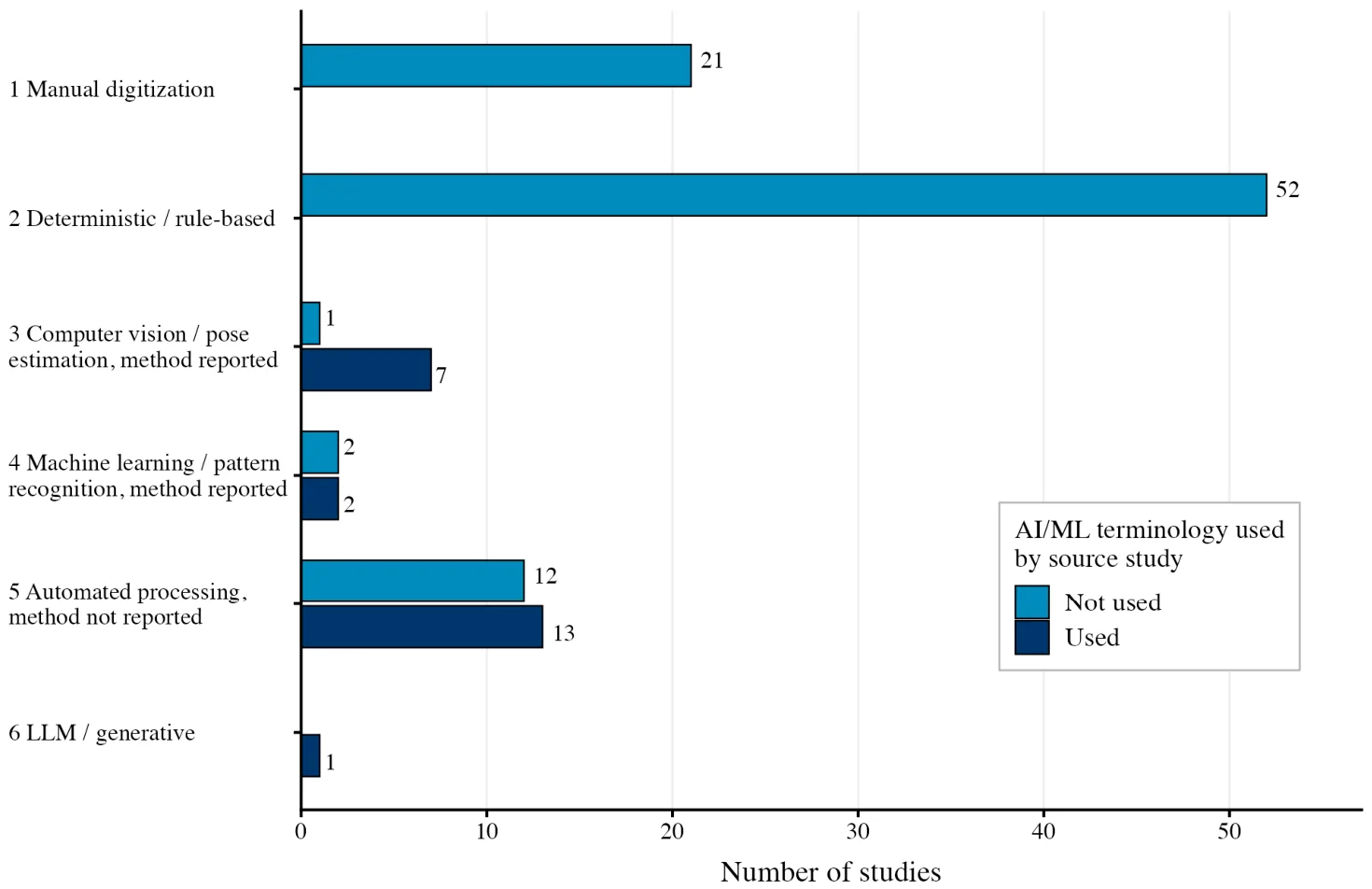
Computational class of the evaluated mHealth apps by reported artificial intelligence terminology. Bars show the number of studies (*n* = 111) in each computational class, split by whether the source study described the app using artificial intelligence, machine learning, or deep learning terminology. Terminology use is largely independent of the disclosed method: 13 of the 23 studies using such terminology reported no algorithm, while 12 of the 25 apps performing undisclosed automated processing were not described in these terms.

A further feature synthesized from this review was the opacity of the analytic methods underlying many apps: most studies did not disclose how their apps’ measurements or classifications were actually computed. Computational approaches spanned all six classes coded in this review: manual digitization (*n* = 21, 18.9%) and deterministic, rule-based computation (*n* = 52, 46.8%) together accounted for nearly two-thirds of studies; computer vision or pose-estimation (*n* = 8) and machine learning methods (*n* = 4) each had a disclosed method, as did the single large-language-model application (*n* = 1), together yielding 13 studies (11.7%) with a genuinely disclosed computational method; and the remaining 25 studies (22.5%) performed an automated computation the source study never described. Processing location was similarly unreported in 83 studies (74.8%). Reported terminology bore little relation to disclosed method: of the 23 studies describing their app as artificial intelligence, machine learning, or deep learning, most disclosed no algorithm, while many apps performing undisclosed automated processing were never labeled as artificial intelligence at all (see Figure 5). The label therefore certified neither the presence of a method nor its disclosure. This opacity was concentrated in proprietary commercial tools and in the most recently emerging, AI-based application areas, precisely where claims were strongest and independent scrutiny weakest.

This matters because an undocumented method cannot be independently reproduced or verified, a concern now well-recognized across medical artificial intelligence, where the absence of shared code, data, and model detail has been shown to undermine the reproducibility of published findings [137]. This is compounded in the mHealth setting by version instability: a validated result applies only to the specific app version, operating system, and capture configuration under which it was obtained, and several apps in this review behaved differently across releases or operating modes [39,40,41]. When the underlying method is proprietary and undocumented, there is no way to know whether a later release preserves the validated behavior, so accuracy established for one version cannot be assumed for the tool a practitioner actually downloads. Post hoc explainability techniques do not resolve this, since they approximate rather than reveal a model’s actual computation and can foster a false sense of assurance [138]; the requirement is disclosure and independent validation, not explanation after the fact.

This review’s focus, like the underlying literature, has centered on the accuracy of an app’s output rather than on the hardware and firmware that produce it. Built-in smartphone sensors and paired peripherals were treated in this review as a fixed input whose own measurement error was assumed rather than characterized: no included study reported calibration procedures, sensor-level noise characteristics, or firmware-version dependence for the accelerometers, GPS receivers, or camera hardware underlying app measurements, and validation against a criterion instrument (Section 4.3) captures end-to-end agreement without isolating whether errors originate in the sensor, the signal-processing pipeline, or the analytic algorithm. This distinction matters increasingly as apps combine multiple sensing modalities rather than a single stream; several apps in this review already fused inertial, GPS, and camera-derived signals for a single output (Table S4), a design pattern examined more explicitly in adjacent wearable-sensing domains, such as multimodal EEG-EMG fusion for real-time device control [139], where synchronization, signal quality, and fusion-stage error propagation are treated as first-order validation targets rather than assumed. Extending that standard of hardware- and fusion-level scrutiny to sports mHealth apps, not just validating the final output, would close a gap this review’s data extraction could not fill on its own, since none of the included studies reported it.

A related gap concerns the validation of the analytic pipeline itself, as distinct from the single output the app displays. Of the 13 studies that disclosed a computational method, none reported an interpretability analysis beyond describing the algorithm’s stated logic, and none described a process for monitoring or revalidating the pipeline’s performance after deployment, that is, whether an update to the app, its underlying model, or the phone hardware it runs on was checked against the original validation before release. This absence of what might be termed predictive maintenance, proactively tracking a deployed model’s performance for drift rather than waiting for a user-reported failure, is consistent with the version instability already documented across several apps in this review [39,40,41] and represents a second, largely unaddressed layer beyond the disclosure problem discussed above: even a fully disclosed method still requires an ongoing process to confirm it performs as validated once deployed.

Sport science already has the reporting infrastructure needed to address this issue, but these standards have not yet been systematically applied to app-based tools. Existing guidance for artificial intelligence interventions and prediction models, including the SPIRIT-AI and CONSORT-AI extensions for trials [140], the TRIPOD + AI statement for prediction models [141], and domain-specific checklists for consumer wearables that call for reporting device and firmware versions alongside the validation protocols [142], collectively define the level of transparency required for adequate reporting. Studies evaluating mHealth apps should, at a minimum, report the app name, version, and operating system; where an app performs an automated computation, its analytic method, and for machine learning tools, the model type, training data, and validation approach; and the processing location. For AI-based and developer-produced tools in particular, independent replication under documented conditions should be treated as a prerequisite for practice rather than an optional refinement.

### 4.7. Implementation and Governance Considerations

Beyond measurement performance, the studies in this review were uniformly silent on how the athlete data these apps collect is stored, secured, and governed. None of the 111 included studies reported a data-protection or consent framework specific to the app’s ongoing use outside the study protocol, despite many apps collecting continuous physiological, positional, or video data capable of identifying an athlete. This is a documented risk rather than a theoretical one: forensic analysis of consumer fitness apps has recovered detailed location, biometric, and activity histories directly from device storage, including data users believed had been deleted [143]. Consent obtained for a validation study does not extend to an app’s subsequent commercial use, and the present review cannot determine whether the data-handling practices evaluated in a research context match those governing the same app once deployed for routine training use.

A second governance question concerns who has access to app-generated data and who is accountable for decisions made from it. Several categories in this review, particularly self-reported monitoring, physiological measurement, and tactical and match analysis, generate data intended for a coach or practitioner rather than the athlete alone (Section 3.2); yet, none of the included studies described consent, access-control, or data-sharing arrangements between athlete and staff, nor whether athletes retained the ability to view, correct, or withdraw their own data. This raises questions of athlete autonomy distinct from measurement validity: an accurate app can still be deployed in ways that constrain an athlete’s control over personal data, particularly in youth, academy, or employment-linked sport settings where consent may not be freely given. Interoperability was similarly unexamined; most apps in this review operated as closed, single-purpose tools, so data generated by one app rarely transfers to another system a practitioner might use, fragmenting an athlete’s record across tools rather than consolidating it. Finally, responsibility for a decision made on the basis of an inaccurate app output—for example, a return-to-sport clearance informed by a musculoskeletal-screening app with the systematic bias documented in Section 4.3—was not addressed by any included study and remains an open question for practice.

Regulatory classification compounds this gap. Most apps in this review would likely fall outside formal medical-device regulation: under United States Food and Drug Administration guidance, software intended only for general wellness or performance tracking, rather than diagnosis or treatment, is generally exempt from device regulation regardless of the sophistication of its underlying computation [144], and the equivalent European framework draws a comparable line between wellness software and regulated medical device software [145]. This means that an app coded here as automated or machine learning-based (Class 4 or 5, Section 3.2) can reach athletes and practitioners with no external validation requirement at all, provided it is marketed for training rather than clinical use, even where its outputs inform decisions—such as musculoskeletal screening or return-to-sport timing—that carry clinical consequences.

A further equity concern is largely absent from this literature: whether app performance is consistent across the populations that use these tools. None of the 111 included studies reported validation performance stratified by skin tone, sex, age group, or body composition, despite documented evidence that optical sensing, the modality underlying photoplethysmography-based heart-rate and camera-based pose-estimation apps that together account for a substantial share of this review’s evidence base (Section 3.2), is systematically less accurate in users with darker skin pigmentation [146,147]. Because device cost and smartphone camera quality also vary with athlete resources, unequal validity may compound unequal access, so that apps validated primarily on samples with limited demographic and technological diversity (Section 4.10) risk performing least reliably for the athletes least able to access alternative measurement. This dimension of validity has not been examined anywhere in the reviewed literature and should be treated as a reporting requirement alongside the accuracy metrics already expected.

### 4.8. Implications for Practice

**Figure 6:**
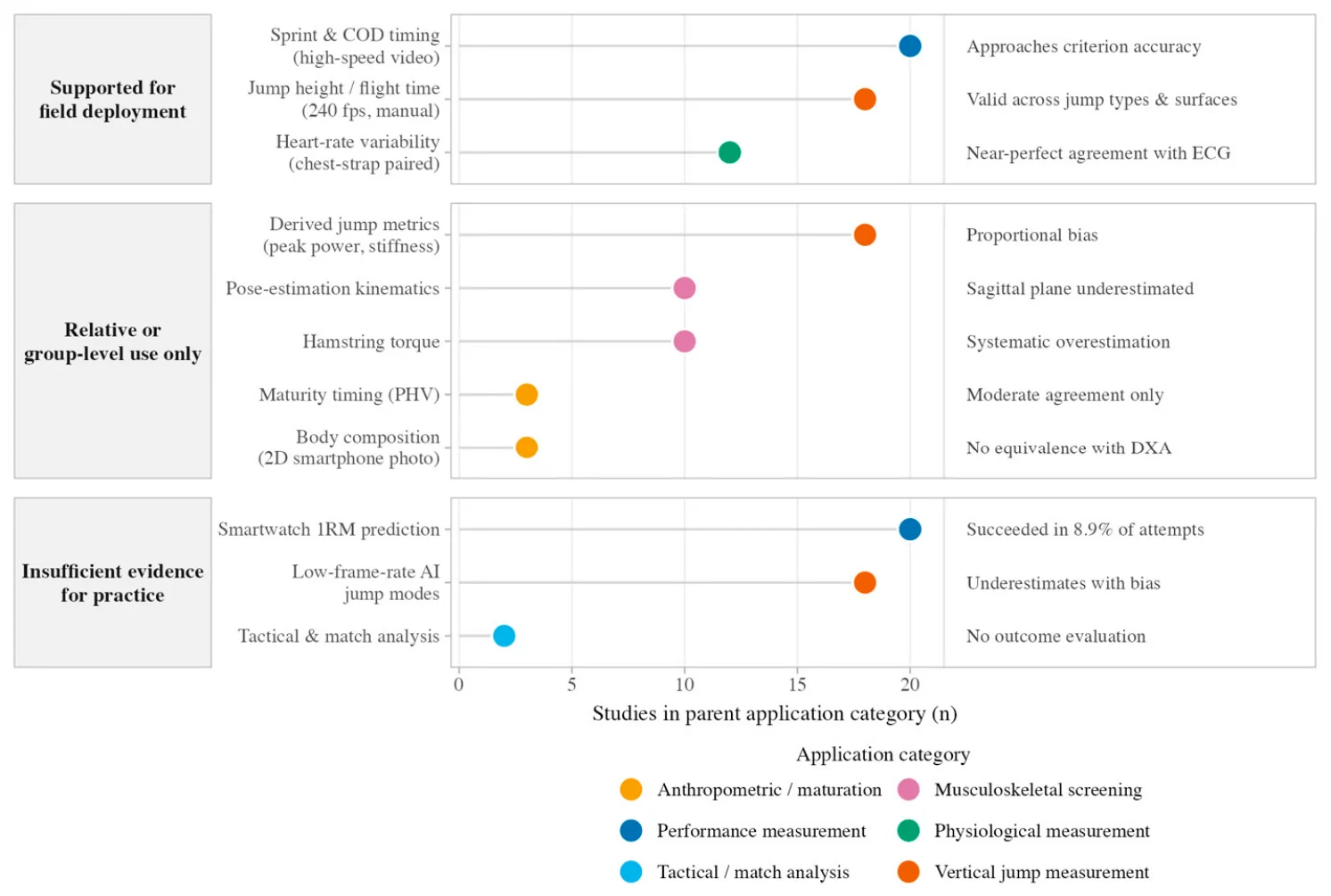
Translational readiness of sport-training app use cases across three tiers of evidence for practice. Representative use cases are grouped into apps supported for field deployment, apps suited to relative or group-level use only, and apps with insufficient evidence for practice. The right-hand label summarizes the key evidence for each placement. Point position on the x-axis shows the number of studies in the use case’s parent application category, indicated by color; it reflects the size of the evidence base, not the strength of support for the tier assignment.

This review’s findings translate into three tiers of practical guidance for coaches and practitioners, summarized in Figure 6: a defined set of apps that may be suitable for field deployment within the specific populations, devices, and configurations in which they were validated, a second set suitable only for relative or group-level use, and a third set not yet supported for practice. High-frame-rate video timing apps can substitute for laboratory instruments in sprint and change-of-direction assessment [12,15], video-based flight-time measurement of jump height at 240 fps approaches criterion accuracy across jump types and surfaces [14,81], and chest-strap-paired HRV apps agree near-perfectly with electrocardiography [17,34]. Practitioners without access to timing gates, force platforms, or laboratory ECG can reasonably adopt these tools for the measurements and configurations in which they were validated.

A second group of apps, those that rank athletes correctly but carry systematic bias, should be restricted to relative or group-level use. Derived metrics were consistently less robust than the primary variables from which they were calculated [78], and app-based hamstring torque, pose-estimation kinematics, smartphone body composition, and the timing of peak height velocity were each adequate for ranking or profiling but not for individual clinical or selection decisions [21,84,123]. A third group is not yet supported for practice, including tactical and match analysis apps, which have never been evaluated against competitive outcomes, and smartwatch-based and low-frame-rate automated modes, which failed or biased substantially where tested [40,87].

Three cautions apply across all of these use tiers. First, validity is a property of a specific app, version, and configuration rather than of a class of technology (Section 4.3), so practitioners should confirm that published evidence corresponds to the exact tool and settings they intend to use. Second, because systematic bias is common but stable, apps are best used to track within-athlete change under a fixed protocol rather than to generate values comparable with criterion instruments. Last, because adherence rather than accuracy determined value in every longitudinal deployment reviewed (Section 4.4), adopting an app is a commitment to the coaching and feedback structures that sustain its use, which organizations should budget for, rather than treating procurement as the substantive decision. This commitment differs by intended operator: athlete self-operated tools (Section 3.2) depend on individual engagement and require no additional staff time, whereas the coach- or practitioner-operated tools that dominate the measurement categories (including most vertical jump and performance measurement apps) require dedicated staff time for testing and interpretation, a resource cost that should factor into adoption decisions alongside validity. Concretely, based on the current evidence, coaches and athletes without laboratory equipment can adopt MySprint or CODTimer for sprint and change-of-direction timing, the My Jump family (My Jump, My Jump 2, My Jump Lab) for vertical jump height at 240 fps, and chest-strap-paired HRV apps such as ithlete or HRV4Training for autonomic monitoring, provided they are used within the populations, jump types, and configurations in which they were validated.

### 4.9. Implications for Research

The next research priority is to move beyond measurement validation and determine whether app-guided decisions translate into improved athlete outcomes. Because measurement feasibility is now established across most of the training process (Section 4.2), the questions of greatest value concern whether app-guided decisions change outcomes: whether load management informed by app-derived HRV reduces injury or improves adaptation, whether app-based screening changes injury incidence, and whether app-delivered technique feedback accelerates skill acquisition relative to conventional coaching. Such questions require controlled designs with athlete outcomes as endpoints rather than agreement with a criterion instrument. This reorientation also implies treating adherence, reach, and dropout as primary endpoints and reporting the coaching structures within which an app was deployed (Section 4.4). Existing reporting standards for eHealth and mHealth evaluations offer a ready framework, including guidance on describing the intervention in sufficient detail for replication [148], and are readily extendable to non-randomized evaluations.

Where validation remains necessary, both its conduct and reporting should be strengthened. Studies should be adequately powered, independently conducted, and prospectively registered. App developers should report systematic bias, limits of agreement, and formal tests of proportional bias alongside relative indices, with all results interpreted against predefined analytical or clinical decision thresholds. Because validity was app-, mode-, and version-specific, studies should routinely report the app version, operating system, and capture configuration. Independent replication under different conditions should also be encouraged and recognized as an essential component of the evidence base. The concentration of developer-conducted evidence in the newest application areas makes independent corroboration a particular priority for AI-based tools, where claims are currently strongest and scrutiny weakest.

Finally, the evidence base should be expanded across several dimensions. Research remains concentrated in Spain and the United States and focuses predominantly on soccer and running, limiting the generalizability of the findings across other sports, competitive levels, and resource settings. Female athletes appear in this literature almost exclusively through menstrual-cycle tracking and self-reported monitoring rather than across the measurement categories, and para-athlete, youth, and masters populations are largely absent (Section 3.1). Although assistive technologies and machine learning applications in para-athlete sport have been synthesized elsewhere [149,150,151], app-based monitoring in these populations remains largely unexamined and is a priority for future work. Several application areas rest on three or fewer studies and warrant primary evidence before synthesis is meaningful. One further issue went unexamined across the entire review and merits attention in its own right: the longevity and deprecation of apps, given that validated versions are routinely superseded or withdrawn. The governance of the athlete data these tools collect, comprising privacy, consent, interoperability, and regulatory classification, is addressed in Section 4.7.

### 4.10. Strengths and Limitations of This Review

This review has several strengths. Its scope is broader than that of prior work in the area, searching five databases from inception to July 2026 and screening 9476 records to map mHealth apps across the entire sport-training process rather than within a single measurement domain, technological modality, or population. In the absence of any established taxonomy of sport-training apps, the ten application categories were derived inductively from the extracted data, providing a structure that subsequent work can adopt or contest. Methodological quality was appraised with a design-specific instrument suited to the heterogeneity of the evidence base [32], and the distribution of study designs across categories was displayed as an evidence gap map, making the structural imbalance described in Section 4.2 directly visible. The review protocol was registered on the Open Science Framework, and a full study-by-study listing of all 111 studies is provided in the Supplementary Materials, allowing readers to interrogate the synthesis at the level of individual studies.

Several limitations should be considered when interpreting these findings. Screening and data extraction were conducted primarily by the first author, with uncertain cases resolved in discussion with the corresponding author, rather than through fully independent dual screening. Prior methodological work indicates that experienced single reviewers miss relatively few eligible studies compared with double screening [152], though this approach means a small number of eligible studies may not have been captured and some extraction errors may not have been caught by a second reviewer. To partly address this, a second reviewer independently duplicate-coded a stratified random sample of 35 studies (32% of the review, spanning all ten application categories), blind to the original codes, and agreement was quantified with Cohen’s κ (Section 2.8). Agreement was almost perfect for primary application category (κ = 0.94) and MMAT design category (κ = 1.00), almost perfect for sensor source (κ = 0.84), and moderate for computational class (κ = 0.56) and intended operator (κ = 0.59). Agreement on the non-exclusive secondary-function tags ranged from fair to almost perfect across the four functions (per-label κ = 0.24–0.86), and was more limited for several individual MMAT quality-appraisal criteria (C1–C5, κ = 0.00–0.55 across Categories 2–4), particularly those requiring judgments about sample representativeness and nonresponse bias that are difficult to assess from extracted text alone. Disagreements were concentrated in computational-class and operator codes for validation-focused apps where operation can plausibly be either athlete- or coach-administered (e.g., video-based jump- and lift-measurement tools); category assignment, the review’s central classification, showed the strongest agreement of any coded variable. Because computational class, intended operator, and several secondary-function tags depend on judgment calls by the coding reviewer rather than on information stated explicitly in the source text, and because agreement on these particular variables was only moderate to fair, these classifications should be interpreted as descriptive characterizations of how each app appeared to be used and coded rather than as definitive or independently verifiable attributes of the apps themselves.

The search was restricted to English-language records and to five bibliographic databases, so non-English studies, gray literature, and apps evaluated outside the peer-reviewed literature were not captured; given that the field’s commercial activity substantially outpaces its publication record, the apps identified here are unlikely to represent those actually in use. Furthermore, this review did not include conference papers and could result in the potential omission of technical reports of mHealth apps. This disciplinary skew toward health and sport-science databases may partly explain the limited algorithmic transparency reported in Section 3.2 and Section 4.6, since journal articles in engineering-focused venues may report computational methods in more technical detail than clinically oriented outlets. Further research may need to include engineering databases for a more comprehensive account of all existing mHealth apps in sport training.

Three further constraints follow from the review’s design. As a scoping review intended to map an emerging and heterogeneous field, it synthesizes narratively and does not pool results or rate certainty of evidence formally, so the conclusions describe the shape of the evidence rather than quantify effects. Assigning each app to a single mutually exclusive category imposes clarity on tools that frequently served more than one function, and readers should treat the category boundaries as an analytical convenience rather than a property of the apps themselves. Finally, this is a fast-moving target: apps are updated, superseded, and withdrawn more quickly than they can be evaluated and published, so the specific tools and versions described here will date faster than the structural patterns that organize this discussion.

## 5. Conclusions

This scoping review identified 111 studies evaluating mHealth apps across ten application categories spanning performance measurement, training load and recovery monitoring, skill development, injury screening, and psychological and nutritional support. The literature has grown rapidly, and validated apps can now measure variables such as jump height, sprint timing, barbell velocity, and heart-rate variability with accuracy approaching laboratory instruments, within the specific populations, versions, and configurations tested.

However, the field remains organized around measurement rather than outcomes: validation studies dominate while controlled effectiveness trials remain scarce, good relative validity does not guarantee absolute accuracy, and adherence rather than accuracy determined real-world value wherever apps were deployed longitudinally. A defined set of validated apps may be suitable for field use now, within the populations, devices, operating systems, and configurations in which they were validated; this is distinct from their use for diagnosis, injury prediction, athlete selection, or return-to-sport clearance. Future research should prioritize controlled trials linking app-guided decisions to athlete outcomes, transparent algorithmic reporting, and implementation science addressing adherence.

## Figures and Tables

**Table 1:** Application categories of mHealth apps used in sport training, with operational definitions, the most-studied apps, and the number of included studies in each category.

Application Category	Definition	Most-Studied Apps	Studies (*n*)
Sport skill training	Apps supporting the development and analysis of sport-specific technique and movement quality, typically providing video- or pose-estimation-based feedback during skill execution.	SwingVision (v9.8.3); RunningCoach (2); BESTGYM PoseApp; ABCapp; predominantly single-study research prototypes	26
Performance measurement	Apps quantifying discrete performance qualities such as sprint and change-of-direction time, movement velocity, and estimated one-repetition maximum, via high-speed video or timing.	CODTimer (3); MySprint (2); PowerLift/My Lift; Qwik VBT; iLOAD	20
Vertical jump measurement	Apps measuring vertical jump height and related variables (flight and contact time, reactive strength) from high-speed video of jump tasks.	My Jump/My Jump 2/My Jump Lab (15); VertVision; Jump Power; Jumpster	18
Self-reported monitoring	Apps collecting athlete-reported wellness, readiness, recovery, and perceived-load data through questionnaires or diaries.	PMSys (2); TrainingPeaks; SMARTABASE; Titan Athlete	12
Physiological measurement	Apps assessing internal physiological load or status, most commonly smartphone-derived heart-rate variability or metabolic thresholds.	ithlete (5); HRV4Training (3); Elite HRV; CameraHRV (v5.0.9)	12
Musculoskeletal screening	Apps for musculoskeletal assessment, injury risk screening, or return-to-sport evaluation, including joint range of motion and movement quality measurement.	Strengthen your Ankle (2); My Jump Lab; PHAST; Clinometer (v2.4)	10
Psychological intervention	Apps delivering psychological support or skills training, such as stress management, relaxation, breathing regulation, or cognitive techniques.	WorryTree; BreathPacer; REMBO; TalentCards	5
Nutrition	Apps supporting dietary and nutritional monitoring or education for athletes.	MyFitnessPal; MealLogger (v4.6); NutriFit-AI	3
Anthropometric and maturation screening	Apps assessing body composition, anthropometry, or biological maturation.	Maturo (2); MeThreeSixty	3
Tactical and match analysis	Apps supporting tactical analysis or match and competition performance monitoring.	SoniSailing; PoloTrac	2

Note: Apps are ordered by category size. Numbers in parentheses indicate the number of included studies evaluating that app or app family where more than one; apps without a parenthetical number were each evaluated in a single study. The “My Jump” family aggregates My Jump, My Jump 2, and My Jump Lab. Software version is given in parentheses where reported by the source study for a single-study app; version was not reported for most apps, and for app families spanning multiple studies (e.g., the My Jump family, CODTimer, MySprint), version differs by study and is listed individually in Table S2. Many apps, particularly in sport skill training, were custom research prototypes; a full study-by-study list of all apps is provided in the Supplementary Materials (Table S2).

**Table 2:** Summary of the evidence base for mHealth apps in sport training, by application category.

Application Category	*n*	Dominant Design(s)	Sample Size (Range)	Populations/Main Sports	Evidence Summary
Sport skill training	26	Usability (8), validation (7)	1–196	Recreational to trained; 16 sports, most often running, table tennis	Validated camera/pose systems are accurate, but small proof-of-concept studies dominate and controlled trials are scarce; most apps remain at an early validation stage.
Performance measurement	20	Validation (18)	2–62	Trained and competitive athletes	High-speed-video timing apps can substitute for laboratory timing in the field; sensor- and watch-based tools remain more variable, and validity is app- and version-specific.
Vertical jump measurement	18	Validation (18)	9–88	Youth and recreational to elite/Olympic	High-frame-rate flight-time apps substitute for laboratory systems; automated, AI, and alternative sensing modes require app- and version-specific validation before interchangeable use.
Self-reported monitoring	12	Cohort (4), other (3)	11–400	Elite and collegiate; prominent women’s soccer	Feasible and useful for load management and communication rather than predicting performance; implementation is a greater challenge than measurement.
Physiological measurement	12	Validation (6), cohort (5), cross-sectional (1)	7–63	Collegiate, elite, professional endurance and team sport	Valid against ECG (most accurately with a chest strap) and sensitive to training-load-induced autonomic change; constrained by inter-individual variability and adherence.
Musculoskeletal screening	10	Validation (6), RCT (2)	7–25,781	Collegiate, professional, recreational	Improve access to risk screening and prevention and are acceptably valid for relative/group-level screening, but limited absolute accuracy and poor real-world adherence constrain standalone clinical use.
Psychological intervention	5	RCT/controlled (4), mixed methods (1)	8–425	Small mixed-sport samples	Interventions directly targeting a mental health or belief outcome show early promise; indirect or repurposed approaches have not shown benefit, and low adherence recurs.
Nutrition	3	RCT (2), usability (1)	17–152	Student, adolescent, elite	Feasible and effective for dietary knowledge and self-reported behavior, but the evidence base is very small, short, and self-report-reliant, with the strongest effects from the weakest reporting.
Anthropometric and maturation screening	3	Validation (3)	41–103	Youth athletes	Camera-based maturation screening is promising for most maturity indices, whereas smartphone body composition is not yet interchangeable with DXA; the small, developer-conducted evidence warrants caution.
Tactical and match analysis	2	Usability (2)	5–13	Windsurfing, water polo	Very limited evidence, confined to small usability pilots; quantitative effectiveness has not yet been evaluated.

Note: Design counts reflect each study’s primary design as coded during data extraction; only the two most frequent designs are listed per category, except where a third design is required for the counts to total *n*. Sample size (range) refers to the number of participants; where a study reported no participant count, it is excluded from the range. Full study-level data are reported in Table S2.

**Table 3:** Technical characteristics of the evaluated mHealth apps by application category.

Application Category	*n*	Sensor Source				Computational Class						AI/ML Terminology Used
		Built-In Only	None (Manual Entry)	External Only	Built-In + External	1 Manual Digitization	2 Deterministic/Rule-Based	3 CV/Pose est., Reported	4 ML/Pattern Recog., Reported	5 Automated, Not Reported	6 LLM/Generative	
Sport skill training	26	16	1	5	4	1	11	6	3	4	1	10
Performance measurement	20	18	1	1	0	7	6	1	0	6	0	2
Vertical jump measurement	18	18	0	0	0	12	0	0	0	6	0	3
Self-reported monitoring	12	0	9	3	0	0	10	0	0	2	0	2
Physiological measurement	12	3	0	6	3	0	12	0	0	0	0	0
Musculoskeletal screening	10	7	3	0	0	1	5	1	0	3	0	3
Psychological intervention	5	1	4	0	0	0	5	0	0	0	0	0
Nutrition	3	2	1	0	0	0	2	0	0	1	0	1
Anthropometric and maturation screening	3	3	0	0	0	0	0	0	0	3	0	2
Tactical and match analysis	2	1	1	0	0	0	1	0	1	0	0	0
Total	111	69	20	15	7	21	52	8	4	25	1	23

Note: Counts are studies, not apps: an app evaluated in more than one study contributes once per study, and the same app may fall into different computational classes across studies where different versions or operating modes were evaluated. Sensor source and computational class are each mutually exclusive, so rows sum to the category *n*; the AI/ML terminology column is independent of computational class and does not sum to the row. Percentages are not reported within categories because several contain fewer than five studies. Abbreviations: CV, computer vision; est., estimation; LLM, large language model; ML, machine learning; recog., recognition.

## Data Availability

The data supporting the reported results are available in the Supplementary Materials (Tables S1–S6) and in the review’s Open Science Framework registration (https://doi.org/10.17605/OSF.IO/BZH6J).

## Supplementary Materials

The following supporting information can be downloaded at: https://www.mdpi.com/article/10.3390/s26175394/s1, Table S1. Full electronic search strategy for each database. Table S2. Characteristics of included studies, grouped by application category. Table S3. Methodological quality appraisal of included studies using the Mixed Methods Appraisal Tool (MMAT, version 2018). Table S3a. Frequency of MMAT criterion ratings across included studies. Table S3b. Per-study MMAT ratings. Table S4. Technical characteristics of the evaluated mHealth apps, by study. Table S5. PRISMA extension for scoping reviews (PRISMA-ScR) checklist. Table S6. Cross-category matrix of secondary app function.
