# Supplementary material for: Mobile Health (mHealth) Apps in Sport Training: A Scoping Review
Source: Sensors (Basel). 2026 Aug 26;26(17):5394. doi: 10.3390/s26175394 (PMC13568394; doi:10.3390/s26175394)
Supplement: Supplementary file 1 [file sensors-26-05394-s001.zip › sensors-4488743-supplementary.pdf]

Table S1. Full electronic search strategy for each database.

Five databases were searched from database inception to July 2026 and limited to English-language records. In each database, three concept blocks (sport or athlete population; mobile technology; and function or purpose) were combined with the Boolean operator AND, and synonyms within each block were combined with OR. Terms were applied to title, abstract, and keyword fields (or the equivalent topic or all-field option) and mapped to database-specific controlled vocabulary where available.

Database	Search string	Records (n)
**PubMed (MEDLINE)**	("Sports"[Mesh] OR sport*[tiab] OR athlete*[tiab]) AND ("Mobile Applications"[Mesh] OR "mobile app*"[tiab] OR "smartphone app*"[tiab] OR smartphone[tiab] OR "mobile health"[tiab] OR mHealth[tiab]) AND (training[tiab] OR performance[tiab] OR monitoring[tiab] OR "injury prevention"[tiab] OR injury[tiab] OR rehabilitation[tiab] OR coaching[tiab] OR "self-management"[tiab] OR intervention[tiab])	1,004
**Scopus**	TITLE-ABS-KEY(sport* OR athlete*) AND TITLE-ABS-KEY("mobile app*" OR "smartphone app*" OR smartphone OR "mobile health" OR mHealth) AND TITLE-ABS-KEY(training OR performance OR monitoring OR "injury prevention" OR injury OR rehabilitation OR coaching OR "self-management" OR intervention)	1,911
**Web of Science (Core Collection)**	TS=(sport* OR athlete*) AND TS=("mobile app*" OR "smartphone app*" OR smartphone OR "mobile health" OR mHealth) AND TS=(training OR performance OR monitoring OR "injury prevention" OR injury OR rehabilitation OR coaching OR "self-management" OR intervention)	3,351
**SPORTDiscus (EBSCO)**	AB (sport* OR athlete*) AND AB ("mobile app*" OR "smartphone app*" OR smartphone OR "mobile health" OR mHealth) AND AB (training OR performance OR monitoring OR "injury prevention" OR injury OR rehabilitation OR coaching OR "self-management" OR intervention)	1,945
**Embase (Elsevier)**	('sport'/exp OR sport*:ti,ab OR athlete*:ti,ab) AND ('mobile application'/exp OR 'mobile app*':ti,ab OR 'smartphone app*':ti,ab OR smartphone:ti,ab OR 'mobile health':ti,ab) AND (training:ti,ab OR performance:ti,ab OR monitoring:ti,ab OR 'injury prevention':ti,ab OR injury:ti,ab OR rehabilitation:ti,ab OR coaching:ti,ab OR 'self-management':ti,ab OR intervention:ti,ab)	1,265
**Total records identified**		**9,476**

Notes: [tiab] = title/abstract field (PubMed); TITLE-ABS-KEY = title, abstract, and keyword fields (Scopus); TS = topic field (Web of Science); AB = abstract field (SPORTDiscus/EBSCO); /exp = Emtree explosion and :ti,ab = title/abstract fields (Embase). The asterisk (*) denotes truncation. Record counts reflect results retrieved on the date of search, prior to duplicate removal.

Table S2. Characteristics of included studies, grouped by application category.

N, total sample size as reported in the primary study; NR, not reported. Age is the mean age (or, where reported by subgroup, the value for the first-listed subgroup) as stated in the primary study; NR, not reported. Sex is the reported male/female split of the study sample; NR, not reported. Platform is the app operating system as reported (device details omitted). Studies are ordered by application category (descending number of studies) and first author. * Age range and sample size for Kholboeva et al. (2025) are reported inconsistently in the original article (age: 12–17 vs. 13–17 years; N: 120 abstract, 200 methods, 152 final analytic cohort with 94% retention); values used in this review are marked with an asterisk. See Table S3b for full detail.

Study	Country	App	Platform	Sport	Competitive level	Study design	N	Age	Sex	Primary outcome(s)
**Sports skill training (n = 26)**										
Aranki (2017)	United States	RunningCoach	Android	Long-distance running	Recreational (long-distance runners per IAAF definition; level not explicitly stated) (unclear)	Usability	6	NR	50% M / 50% F	Usability, acceptability, perceived accuracy of the system (qualitative case studies)
Aranki (2018)	United States	RunningCoach	Android	Long-distance running	Mixed (long-distance runners per IAAF definition; some competitive, some recreational) (unclear)	Usability	6	Not reported (range 23–26 years)	50% M / 50% F	Feasibility and usability — battery consumption, system robustness/fault-tolerance, perceived measurement accuracy, acceptability, privacy
Bilal (2025)	United Arab Emirates	Not reported (unnamed React Native app; framework prototype)	Not reported	Cue sports (billiards/pool)	Amateur	Usability	25	18–35 years (range; mean not reported)	NR	Usability (SUS), perceived feedback accuracy, feedback latency
Bilić (2023)	Croatia	SwingVision (SV) (v9.8.3)	iOS	Tennis	Elite (elite male junior players, national-ranked, U18)	Validation (concurrent validity)	5 players (analysis based on 60 serves + 100 match shots per player)	15.6 ± 0.35 years	100% M / 0% F	Concurrent validity (ICC) of SwingVision vs criterion measures — radar for serve speed; video analysis for placement, stroke type, and spin
Boratto (2017)	Italy	u4fit	Both	Running	Recreational	Cohort (retrospective analysis of platform usage data)	103	41.35 y (M) / 38.80 y (F)	60% M / 40% F	Engagement by coaching type (share of completed workouts), training frequency (active weeks), adherence (completed/planned sessions)
Cabahug (2025)	Taiwan	BESTGYM PoseApp	iOS	Gymnastics (artistic — horizontal bar giant swing)	Not reported (university gymnastics training hall)	Other (system development with feasibility demonstration)	1 (one gymnast, sample data collection)	NR	NR	Giant-swing rotation/turn detection (turn attempts, perfect turns); pose-detection reliability
Ha (2011)	South Korea	Not reported (self-developed iPhone Poomsae education app)	iOS	Taekwondo	Not reported (Taekwondo academy trainees)	Cross-sectional (survey with multiple regression)	196	NR	NR	Effect of app (entertaining and useful factors) on educational effect, reregistration intention, and recommendation intention
Hadza (2026)	Slovakia	MyJump2	Both	Karate (kata)	Elite (top-level international-level youth kata karatekas;	Validation (intra- and inter-rater reliability)	12	15.5 ± 1.1 years	0% M / 100% F (12 F)	Intra-rater and inter-rater reliability (ICC, SEM, SWC, mean difference,

Study	Country	App	Platform	Sport	Competitive level	Study design	N	Age	Sex	Primary outcome(s)
					cadet/junior, ≥2nd kyu)					Cronbach's alpha) of MyJump2 execution-time for four techniques (double-punch, direct kick, kick-return, step-punch) by experienced vs inexperienced rater
Hsu (2025)	Taiwan	ABCapp (AI-powered Badminton Coach app)	Android	Badminton	Mixed (education trial: university beginners in an elementary badminton course; model dataset from competitive NTHU badminton team)	Mixed methods (ML model development/validation + controlled educational trial with experimental vs control groups)	143 enrolled (138 analysed after exclusions); model dataset 102 swing videos	NR	Not reported (dataset 'different genders'; education-trial sex not stated)	Key-frame detection accuracy (Recall, F1-score, Average Positional Error) for the models; badminton-skill learning outcomes (clear-distance test, expert manual scoring, self-perception) for the app trial
Janssen (2020)	Netherlands	Inspirun (e-Coach)	Android	Running	Recreational	Mixed methods (end-user experience: Likert questionnaires + open-ended + app data)	19 (completed 20 sessions; 16 completed final questionnaire)	35 years (range 21–60)	47% M / 53% F	End-user experience of training-session personalization; goal perception; motivation to keep running
Ma (2025)	China	Not reported (mainstream table tennis training app, unnamed; iterated prototype)	Not reported	Table tennis	Amateur	Mixed methods	44 (video experiment; 80 valid questionnaires) + 11 (usability evaluation)	NR	NR	Intrinsic motivation (IMI), cognitive load (NASA-TLX), usability (SUS)
Mat Sanusi (2021)	Germany	Table Tennis Tutor (T3) — LHAPP4	Android	Table tennis	Recreational	Validation	1	NR	100% M (data participant)	LSTM stroke classification accuracy, precision, recall
Muslimin (2024)	Indonesia	Not reported (Android-based digital volleyball skill test instrument)	Android	Volleyball	Amateur (school/university players, ≥2 y training experience)	Validation (R&D instrument development with small/large-scale trials + t-test)	52	14–20 years (range)	Not reported (mixed male and female)	Feasibility/eligibility ratings (originality, innovation, usability, safety, use); manual vs digital test comparison
Oommen (2018)	Canada	Wearable Electronic Swim Coach	Android	Swimming	Not reported	Usability	3	NR	NR	End-of-lane detection success, lane-drift (left/right) detection success, false-positive rate
Park (2017)	South Korea	Motion Log Skateboard	Android	Skateboarding	Mixed (2 beginners, 1 intermediate, 2 professionals)	Usability	5	28.6 ± 6.1 years	60% M / 40% F (3 M / 2 F)	Concept evaluation and usage context of the Motion Log Skateboard (experience session + semi-structured interviews) — utility of shared non-visible pressure information
Reynell (2012)	South Africa	Tilt 'n Roll++	Other	Skateboarding	Not reported (expert skateboarders, consistent kickflip)	Usability	3	NR	NR	Perceived usefulness/value of Tilt 'n Roll++ modifications (semi-structured interviews) — trick visualisation and GPS

Study	Country	App	Platfor m	Sport	Competitive level	Study design	N	Age	Sex	Primary outcome(s)
										information
Silva (2026)	Portugal	Not reported (unnamed Shotokan Karate posture app; Flutter/Dart, Google ML Kit)	Both	Karate (Shotokan)	Not reported (single adult practitioner)	Usability (single-participant feasibility pilot with descriptive quantitative comparison)	1	NR	Not reported (single male author-participant, inferred)	Agreement of app-estimated knee and shin angles with single-expert reference annotations; stance-classification agreement
Steff (2026)	Romania	Tapd: A Coordination Game; Hand eye test; Tap Of War; Color Reaction; Sound Buttons/Instant Buttons; Lights: A Memory Game	Both	Basketball	Amateur (national junior level, U13–U14)	Non-randomized controlled trial	40	EG 13.25 ± 0.72; CG 12.95 ± 0.69 years	100% M / 0% F	Tapd coordination test (right/left hand); Hand–eye coordination test (right/left hand)
Stöggl (2014)	Austria / Sweden	Not reported (custom Smartphone gear-classification application/algorithm)	Android	Cross-country skiing (ski skating)	Mixed (regional-to-international level)	Validation (algorithm development and validation vs video gold standard)	11	NR	63.6% M / 36.4% F (7 M / 4 F)	Accuracy of automatic smartphone gear classification (G2L, G2R, G3, G4L, G4R) vs video analysis
Tate (2017)	United States	SPLnFFT Noise Meter (v5.2)	iOS	Running	Recreational (≥9.7 km/wk, ≥30 min continuous runs)	Other — controlled laboratory study, within-session pre-post design	14	23.7 ± 2.0 years	28.6% M / 71.4% F	Vertical impact peak (VIP), peak vertical instantaneous loading rate (VILR), vertical average loading rate (VALR) during overground running
Umek (2014)	Slovenia	Not reported (golf-swing trainer application; autonomous wearable personal training system)	iOS	Golf	Mixed (one professional golfer plus experienced amateur players)	Other (system development/prototype with field test)	Not reported (player groups of differing skill; 1 professional + amateur golfers; exact N not stated)	NR	NR	Feasibility/effectiveness of real-time audio biofeedback for detecting and correcting head-movement errors during the golf swing
Viyanon (2016)	Thailand	SwingPong	Android	Table tennis	Mixed	Usability	9	24.5 years (evaluation users); 24.25 ± 0.5 years (training data players)	78% M / 22% F (evaluation)	Stroke classification accuracy (forehand, backhand)
Yamagiwa (2014)	Japan	Ski Trainer	Android	Alpine skiing	Recreational (middle-level skier; expert data used to build scoring)	Other — proof-of-concept single-case evaluation	1	NR	NR	Change in app-derived skill scores (tempo, balance ratio, balance angle, dynamicity) after using score/message feedback
Yang (2026)	China	CLARIS-T (LitePoseNet-based prototype)	Not reported	Badminton (forehand high clear)	Amateur (beginners)	RCT	30	18–25 (range)	NR	Skill performance (hit placement accuracy, movement technique rating)

Study	Country	App	Platform	Sport	Competitive level	Study design	N	Age	Sex	Primary outcome(s)
Young (2023)	UK	Not reported (custom research app)	Both	Running	Recreational	Validation	31	34.5 ± 9.7 years	64.5% M / 35.5% F	Agreement (ICC) of smartphone-derived gait outcomes vs Vicon 3D motion capture: contact time, swing time, step time, cadence, knee flexion angle, foot strike location
Zosimadis (2023)	Greece	Not reported (custom Android application)	Android	Ten-pin bowling	Mixed	Validation	9	20–67 (range)	NR	Throw quality assessment accuracy, common error detection precision/recall, skill-level classification accuracy
**Performance measurement (n = 20)**										
Balsalobre-Fernández (2017)	Spain / France	Runmatic	iOS	Running (treadmill)	Not reported (healthy male runners familiar with treadmill running; level not stated) (unclear)	Validation	2 (96 steps analyzed)	NR	100% M / 0% F	Concurrent validity and reliability of contact time, aerial time and derived running mechanics vs Optojump Next
Balsalobre-Fernández (2018)	Spain	PowerLift (v2.8)	iOS	Powerlifting (bench press)	Not reported (resistance-trained/powerlifters)	Validation	10	26.5 ± 6.5 years	100% M / 0% F	Mean barbell velocity vs linear transducer (validity/reliability); estimated vs actual 1RM
Balsalobre-Fernández (2019)	Spain	CODTimer	iOS	Soccer (football)	Amateur (adolescent/youth soccer players)	Validation	20	13.85 ± 1.34 years	NR	Concurrent validity and reliability of total time in the 5+5 COD test vs electronic timing gates
Balsalobre-Fernández (2020)	United States	My Lift (v4.0)	iOS	Weightlifting (snatch)	Collegiate	Validation	10	20.9 ± 1.6 years	NR	Peak vertical/forward/backward displacement, peak vertical velocity vs 3D motion capture (validity)
Cetin (2021)	Türkiye	My Lift (formerly Power Lift)	iOS	Weightlifting	Amateur (competitive club)	Validation	10	17.50 ± 1.27 years	100% M / 0% F	Concurrent validity and reliability of My Lift estimated 1RM vs actual 1RM for conventional deadlift and barbell back squat
Chen (2021)	China	COD timer	iOS	Basketball	Amateur (youth; ≥4 y experience)	Validation	62	15.9 ± 1.4 years	NR	Concurrent validity, inter-observer and test–retest reliability of COD timer app (and stopwatch) vs timing gates for 505 COD time
Chiu (2020)	Taiwan	Not reported (self-developed Android app; unnamed)	Android	Badminton	Mixed (university team, department team, and badminton-class players; ~6.6 yrs experience)	Validation (discriminative feasibility — fast vs slow groups)	30 (22 M, 8 F)	20.7 ± 1.6 years	73.3% M / 26.7% F	Discrimination of fast vs slow footwork groups via mean and maximum acceleration resultant (overall and per direction)

Study	Country	App	Platform	Sport	Competitive level	Study design	N	Age	Sex	Primary outcome(s)
Cirilo (2021)	Peru	ABT (Assistant Boxer Training)	Both	Boxing	Mixed (10 amateur + 1 professional)	Usability (satisfaction/usability validation) with technical benchmarking	11 (10 amateur boxers + 1 professional boxer)	NR	Not reported (professional participant male; group composition not stated)	Usability/usefulness (Likert survey); punch-detection accuracy
Marín (2026)	Spain	OCTOcore (v1.8.4); MySprint used for sprint timing	iOS	Football (soccer)	Elite	Cross-sectional	16	17.2 ± 0.9 years	100% M / 0% F	Association between app-measured single-leg deadlift (SLD) core stability and sprint velocity (5–30 m) and change-of-direction speed (V-cut)
Mon-López (2019)	Spain	TargetScan ISSF Pistol & Rifle	iOS	Olympic shooting (air pistol, air rifle)	Mixed (national/international-competition-experienced amateur & semi-professional shooters)	Validation	24 shooters (1440 shots)	NR	83.3% M / 16.7% F	Concurrent validity of app scores vs official Disag RMIV register (paired t-test, ICC, Bland–Altman)
Moreno-Azze (2025)	Spain	Fitnessmeter (v3.3)	iOS	Soccer (football)	Professional (academy of a Spanish second-division club)	Validation	46	15.44 ± 0.81 years	100% M / 0% F	Concurrent validity and reliability of 5+5 m COD time vs photocells (r, ICC, CV, Bland–Altman)
Nie (2025)	United States	SoundTrack	Both	Running (treadmill)	Mixed (novice to experienced runners)	Validation	61 (SoundTrackDB) + 11 (case study)	Not reported (range 18–55)	~64% M / ~36% F (39 M / 22 F of 61)	Cadence estimation error (MAE/MAPE); Ground contact time (GCT) estimation error (MAE/MAPE)
Oberhofer (2021)	Switzerland	StrengthControl	iOS	Strength training (barbell deadlift, bench press, back squat)	Recreational	Validation	30	28.4 ± 6.0 years	46.7% M / 53.3% F (14/16)	Exercise recognition accuracy; repetition count accuracy; 1RM prediction success rate
Pérez-Castilla (2021)	Spain	iLOAD	iOS	Resistance training (back squat, bench press; not sport-specific)	Recreational (physical education students, 2.7 ± 2.7 y resistance-training experience)	Validation (repeated-measures reliability and validity)	20	23.0 ± 2.6 years	100% M / 0% F	Interrater agreement, between-session reliability (CV, SEM) and concurrent validity vs linear velocity transducer (T-Force) for mean set velocity
Renner (2024)	Austria	Qwik VBT; Metric VBT; MyLift (My Jump Lab)	iOS	Powerlifting	Not reported (competitive powerlifters, Wilks ≥300)	Validation	20	26 ± 4.9 years (range 18–38)	70% M / 30% F (14 M / 6 F)	Concurrent validity (SMB, RMSE, R^2) of mean barbell velocity for three apps and one linear transducer vs Vicon across squat, bench press, deadlift
Romanenko (2024)	Ukraine	Reaction Go/No-go	iOS	Taekwondo (ITF; combat sports)	Mixed (qualification 4 Gup to 1 Dan; youth athletes)	Validation (reliability/test–retest of a developed method; two-stage)	41	16.5 ± 2.78 years (overall)	NR	Test–retest reliability of Go/No-Go reaction time and error rates; comparison of stimulus-interval and start-mode configurations

Study	Country	App	Platform	Sport	Competitive level	Study design	N	Age	Sex	Primary outcome(s)
Romanenko (2025)	Ukraine	Spatial Perception	iOS	Taekwondo (martial arts)	Mixed (8th Gup to 1st Dan)	Validation	57	18.6 ± 3.52 years (overall)	100% M / 0% F	Reliability (test–retest correlation, Cronbach's alpha, Bland–Altman) and validity of the Spatial Perception app; reaction time and error rate across four test stages
Romero-Franco (2016)	Spain	MySprint	iOS	Sprinting (athletics)	Elite	Validation	12	21.4 ± 3.9 years	100% M / 0% F	Sprint split times, F0, V0, Pmax, DRF
Uysal (2023)	Turkey	CODTimer; Seconds Count; StopwatchCamera	iOS	Multiple (soccer, basketball, volleyball, handball)	Collegiate	Validation	30	20.3 ± 2.6 y	76.7% M / 23.3% F	Validity, reliability, and sensitivity of app-measured Illinois CODS test time vs timing gates
Yamaguchi (2016)	Japan	iPhoneSG	iOS	Baseball	Amateur (intended users: amateur players and fans)	Validation	Not reported	NR	NR	Pitch-speed measurement error vs manual frame-by-frame reference; processing time
**Vertical jump measurement (n = 18)**										
Balsalobre-Fernández (2026)	Spain	My Jump Lab	iOS	Not reported (physically active participants; no specific sport)	Recreational (recreationally active sports-science students)	Validation	17	22.5 ± 1.2 years	NR	Concurrent validity and reliability (within-session, test–retest) of jump height, contact time and DRI vs force platform
Bishop (2022)	Spain	My Jump 2 (v5.0) and CODTimer (v2.0)	iOS	Basketball	Elite (national-level youth)	Validation (test–retest reliability + associations)	30	17.67 ± 1.32 years	NR	Magnitude and direction of interlimb asymmetry (SLCMJ, SLDJ, 505); between-session reliability
Dias (2025)	Portugal	My Jump Lab; VertVision	Both	Handball	Amateur (≥5 y senior experience)	Validation	27	32.11 ± 12.9 years	59% M / 41% F	Concurrent validity and reliability of My Jump Lab and VertVision vs contact mat (Chronojump) for CMJ height; direct app-to-app comparison
Driller (2017)	New Zealand	My Jump	iOS	Not specified (mixed)	Mixed (recreational to highly trained)	Validation	61	20 ± 4 years	49% M / 51% F	Concurrent validity of My Jump vs force plate (jump height, flight time) and inter-scorer reliability
Gallardo-Fuentes (2016)	Chile	My Jump	iOS	Track and field (athletics — sprinters, middle/long-distance runners, throwers)	Elite (national/international-level)	Validation	21	22.1 ± 3.6 years (range 19–29)	66.7% M / 33.3% F (14 M / 7 F)	Concurrent validity (ICC, r) and intersession/intrasession reliability (Cronbach's alpha, CV, interday r) of My Jump vs contact platform for SJ, CMJ and 40-cm DJ height
Lopez (2026)	Spain	Not reported	Android	Multiple	Not reported	Validation	Not reported	NR	NR	Flight time accuracy vs

Study	Country	App	Platform	Sport	Competitive level	Study design	N	Age	Sex	Primary outcome(s)
		(unnamed prototype; dashboard screenshot shows "JumpTracker")		(active athletes from various disciplines)			(dataset of >300 jumps from prior studies; 216 validation samples)			jump mat ground truth, false positive rejection, cross-device performance
Maia da Silva (2025)	Brazil	Jumpster	Both	Multiple (basketball, futsal, volleyball, handball)	Recreational (college/recreational athletes)	Validation	53	21.87 ± 3.84 years	37.3% M / 62.3% F (20 M / 33 F)	Concurrent validity (Pearson r) of Jumpster vs My Jump 2 and force platform; test–retest reliability (ICC)
Medeiros (2024)	Brazil	My Jump 2	iOS	Beach volleyball	Elite (Olympic/World/Brazilian champions)	Validation	11	23.6 ± 6.2 years	0% M / 100% F	Validity of CMJ jump height, flight time and peak power on a sand surface vs force platform (ICC, r, Bland–Altman)
Peng (2024)	China	My Jump 2	iOS	Multiple (fencing, swimming, diving)	Professional (training-experienced athletes)	Validation	29	Fencing M 20.5 / F 21.0 y; swimming/diving M 13.7 / F 12.86 y	~55% M / 45% F (16 M / 13 F)	Concurrent validity and reliability (ICC, Pearson r, Bland–Altman) of CMJAM jump height and flight time vs Kistler force platform
Ríos-Gallardo (2025)	Mexico	My Jump 2	iOS	Basketball	Collegiate	Validation	14	18.8 ± 0.8 years	100% M / 0% F	Squat-jump height, countermovement-jump height, Elastic Index (My Jump 2 vs IR-Mat)
Ríos-Gallardo (2025)	Mexico	My Jump Lab AI (v4.5.9)	iOS	Multiple (recreationally active, basketball, soccer, volleyball)	Mixed (recreational, faculty-level, university, national-level)	Validation	80	20.7 ± 1.7 years	100% M / 0% F	Within- and between-session reliability of AI-app CMJ height; concurrent validity/agreement vs force platform
Soares (2023)	Portugal	My Jump 2	iOS	Judo	Mixed	Validation	21	27.41 ± 7.52 y	71.4% M / 28.6% F	Validity/reliability of My Jump 2 CMJ height vs Chronojump contact platform
Stafylidis (2025)	Greece	MyJump 2	iOS	Soccer	Elite	Validation	21	16.6 ± 1.87 y (95% CI 15.7-17.4)	100% M / 0% F	Validity of MyJump 2 vs Chronojump for CMJ and SJ height
Stojiljković (2024)	Serbia	My Jump 2	Both	Basketball	Amateur	Validation	9	15 ± 0.9 y	0% M / 100% F	Concurrent validity of My Jump 2 vs Kistler Quattro force plate for drop-jump height, contact time and interlimb asymmetry
Sxentürk (2025)	Turkey	My Jump Lab	iOS	Multiple (recreationally active / sports science students)	Recreational	Validation	36	22.2 ± 2.9 years	100% M / 0% F	CMJ height concurrent validity vs force plate

Study	Country	App	Platfor m	Sport	Competitive level	Study design	N	Age	Sex	Primary outcome(s)
Tan (2024)	Malaysia	My Jump 2 (My Jump Lab)	iOS	Multiple (elite/national athletes, various sports)	Elite / Professional (Malaysia national/elite athletes)	Validation (cross-sectional)	88	21.6 ± 4.04 years (overall)	62.5% M / 37.5% F (55 M / 33 F)	Reliability (ICC, CV) of AI-app CMJ height and concurrent validity/agreement (ICC, OLP regression, Bland–Altman) vs force platform
Wang (2024)	China	My Jump 2	iOS	Soccer, Basketball	Collegiate	Validation	20	20.2 ± 1.3 years	100% M / 0% F	Concurrent validity of Kvert vs force platform
Çetin (2024)	Türkiye	Jump Power	iOS	Soccer (football)	Professional	Validation	22	20.73 ± 0.89 years	100% M / 0% F	Concurrent validity and reliability of Jump Power app vs Optojump for SJ and CMJ (jump height, flight time)
**Self-reported monitoring (n = 12)**										
Alexandersen (2025)	Norway	PM Reporter Pro (PMSys)	Not reported	Soccer (football)	Elite / Professional	Cohort	65	21.18 years (range 15–32)	0% M / 100% F	Individual match performance (InStat Index) predicted from pre-match wellness (linear mixed models)
Attigala (2019)	Sri Lanka	Optio	Android	Badminton	Not reported	Other (system development with prototype evaluation)	50 trainees + 1 coach (unclear — testing procedure not described)	NR	NR	Model performance: weight-prediction accuracy (RMSE); stress classification (AUC)
Bertschy (2023)	United States	Titan Athlete (Integrated Bionics)	Not reported	Soccer (football)	Collegiate	Cohort (prospective observational)	23	19.7 ± 1.5 years	0% M / 100% F	Prediction of post-session RPE from pre-session wellness score and sleep duration
Duignan (2019)	Ireland	Not reported (Mobile Athlete Self-Report Measure, M-ASRM; custom/commercial)	Not reported	Gaelic Games (football, hurling, ladies football, camogie)	Elite (amateur sport with professional approach)	Mixed methods (qualitative — semi-structured interviews, thematic analysis)	21	Players 27 ± 3.6; CSS 37 ± 11 years	~86% M / 14% F (18 M / 3 F)	Stakeholder perceptions of M-ASRM implementation and understanding (four themes: clarity of purpose, implementation strategies, players' perceptions, perceived facilitators)
Dupuit (2023)	France	Not reported (custom online menstrual-status monitoring app)	Not reported	Soccer (football)	Professional (French National Second Division)	Cohort (prospective observational, methodological)	19	23.7 ± 4.4 years	0% M / 100% F	Feasibility of on-field monitoring — athlete adherence (completion rate) and compliance (completion before noon); menstrual-status phase/profile determination
Jalote Parmar (2024)	Norway	Coral App	Both	Multiple (skiing, cycling, football, handball, etc.)	Mixed (recreational plus one elite athlete)	Usability	26 athletes + 1 coach	Not reported (range 19–28)	Athletes 100% F (coach male)	System Usability Scale (SUS); User Experience Questionnaire (UEQ)
Johansen (2020)	Norway	PMSys (Reporter	Both	Soccer	Mixed (academy youth to	Other (system	19 (AI	NR	Not reported	Feasibility of AI (LSTM)

Study	Country	App	Platform	Sport	Competitive level	Study design	N	Age	Sex	Primary outcome(s)
		App)		(football)	national/international elite)	description with preliminary observational AI experiment)	experiment); ~400 female players in broader research cohort		(broader cohort described as ~400 female players)	prediction of athlete readiness-to-train from self-reported data
Lebedev (2021)	Russia	Not reported (prototype information system with mobile application)	Not reported	Skiing, speed skating, fencing	Professional (university student-athletes) (unclear)	Cohort	400	Sport 20.02 ± 1.32; Control 20.65 ± 0.54	Sport 50%/50%; Control 46%/54%	Physiological indices (SBP change, HR change, vegetative potential, adaptive potential), psycho-emotional indices (stress signs, stress tolerance, integral psycho-emotional index)
McGuigan (2023)	Australia	TrainingPeaks (basic athlete account)	Not reported	Multiple (triathlon, running, cycling, weightlifting)	Recreational	RCT	35 (analyzed)	42.6 ± 12.0 years (control 48.2±9.4; education 35.9±11.5)	51% M / 49% F (18M/17F)	Adherence to app-based training monitoring (education vs control)
Menaspà (2018)	Australia	Athlete Management System (SMARTABASE, Fusion Sport)	Not reported	Water polo	Elite (Australian Olympic squad)	Validation (construct validity)	16	26 ± 3 years	0% M / 100% F	Individual correlations between AMS s-RPE and heart-rate-based training load (Banister TRIMP, Edwards' method)
Sastre-Munar (2024)	Spain	SALUTRACK (SaluTrack)	Both	Basketball	Elite (youth athletes at the High Sport Centre of the Balearic Islands)	Other — observational feasibility study	11	14.50 ± 1.41 y (M); 14.67 ± 1.15 y (F)	72.7% M / 27.3% F	Feasibility of periodic app-based data collection (weekly completion rate); wellness, pain, urinary incontinence and pain-catastrophizing scores
Spetz (2025)	Sweden	Readiness Advisor (RA) app	Both	Multiple (endurance: XC skiing, long-distance running, orienteering, triathlon)	Elite	Validation	20	24.4 ± 3.5 y	55% M / 45% F	Association between subjective RA ratings and objective counterparts (calculated training load, TST, HRV, RHR, Temp. Dev., CGM TiR, POMS tension/TMD/EE-index)
**Physiological measurement (n = 12)**										
Flatt (2015)	United States	ithlete	Not reported	Soccer (football)	Collegiate (NAIA women's soccer)	Validation (methodological; agreement of 5- and 3-day vs 7-day values, sensitivity to training load)	9	22 ± 1.9 years	0% M / 100% F	Weekly mean and CV of supine/standing lnRMSSDx20 in response to varying training load; agreement of 5- and 3-day with 7-day values
Flatt (2017)	United States	ithlete	Not reported	Swimming (sprint-swimming)	Collegiate (NCAA Division I)	Cohort (observational)	10	Male 21 ± 1.6 y; Female 21 ± 1.5 y	70% M / 30% F (7 M / 3 F)	lnRMSSDmean and lnRMSSDcv (daily HRV mean and coefficient of variation) and perceived wellness (sleep, fatigue, soreness, stress, mood) across

Study	Country	App	Platfor m	Sport	Competitive level	Study design	N	Age	Sex	Primary outcome(s)
										baseline/overload/taper
Flatt (2021)	United States	ithlete	iOS	American college football	Collegiate (NCAA Division I)	Cohort (prospective observational)	20	NR	100% M / 0% F	Seasonal trends in RHR and LnRMSSD by position (linemen vs nonlinemen)
Holmes (2020)	United States	ithlete; HRV4Training	Not reported	Ice hockey; Rowing	Collegiate (Mixed — ACHA Division I club hockey; NCAA Division I rowing)	Cohort (prospective observational — compliance)	63 (16 hockey; 47 rowers analysed)	NR	25% M / 75% F (hockey all male; rowing all female)	Compliance rates of self-measured daily HRV recordings across the competitive season
Iliadis (2021)	Greece	Not reported (custom mHealth monitoring system; Vidavo SA)	Android	Cycling	Elite (high-level nationally/internationally ranked cyclists)	Validation (field evaluation/feasibility of the mHealth system)	16	24.1 ± 4.5 years	100% M / 0% F	Detection of health disorders during cycling and correlation between athlete-reported symptoms and mHealth-recorded findings
Javaloyes (2021)	Spain	HRV4training	Not reported	Cycling (road)	Professional	Cohort	7	NR	0% M / 100% F	Changes in vagal HRV markers (LnRMSSD, LnRMSSDcv) before, during, and after an altitude training camp
Johansson (2026)	Ireland	CameraHRV (v5.0.9)	Not reported	Multiple	Mixed (2 elite/international, 6 highly trained/national, 29 trained/developmental; Tier 2+)	Validation (observational reliability & concurrent validity)	37 (36 for reliability; 1 removed for missing repeat data)	21.95 ± 3.69 years	54% M / 46% F	Intra-session reliability (ICC, CV%) and concurrent validity (MAPE, bias, limits of agreement) of the CameraHRV app for RMSSD
Moya-Ramon (2022)	Spain	Elite HRV; Welltory	Both	Cycling (road)	Professional	Validation	26	NR	Not reported (described as professional male cyclists)	Agreement of app-derived LnrMSSD vs ECG gold standard (supine and seated); validity of 1-min vs 5-min recordings
Pereira (2020)	Brazil	HRV Expert (CardioMood)	Android	Running	Recreational	Validation	31	36.1 ± 6.3 y	100% M / 0% F	Agreement of HRV Expert app (+ Polar H10 strap) vs ECG for HRV indices (time, frequency, nonlinear) in supine and sitting positions
Suarez-Tijeras (2023)	Spain	HRV4Training	Both	Futsal	Amateur (youth cadet)	Cross-sectional	11 (15 recruited, 4 excluded)	15.07 ± 0.55 years	100% M / 0% F	Correlation between HRV parameters (LnrMSSD, SDNN, pNN50, LF, HF, LF/HF) and Yo-Yo IR1 aerobic performance (distance)
Williams (2021)	United States	ithlete	Both	Powerlifting	Mixed (competitive and recreational)	Cohort (repeated-measures, single-arm)	12	Not reported (age range 18–40)	83% M / 17% F	Smartphone-derived LnRMSSD (morning vs pre-testing) across microcycles; sensitivity to training load

Study	Country	App	Platform	Sport	Competitive level	Study design	N	Age	Sex	Primary outcome(s)
de Oliveira (2024)	Brazil	ITStriker	Not reported	Boxing	Mixed (professional & amateur)	Validation	10	27.5 ± 5.6 years	100% M / 0% F	Validity of the app-delivered boxing-specific test (TBOX) to estimate HRMAX and HRDP (anaerobic threshold) vs incremental treadmill test
**Musculoskeletal screening (n = 10)**										
Babouras (2024)	Canada	Custom app (Google MediaPipe / BlazePose; iOS ARKit version)	Android	Multiple (baseball, football, hockey, lacrosse, rugby, soccer, swimming, volleyball)	Collegiate (varsity athletes)	Validation	254	21.47 ± 1.95 years	62.6% M / 37.4% F (159 M / 95 F)	Agreement of knee coronal and sagittal angles (at initial contact, peak valgus, peak flexion) between MediaPipe app and Kinect V2 (MAAE, RMSE)
Martínez-Miguel (2026)	United Arab Emirates	My Jump Lab (version 5.0)	iOS	Soccer	Professional	Validation	31	25.2 ± 5.1	100% M / 0% F	Association (Pearson r, regression) and absolute agreement (Bland-Altman) between app-derived torque/breakpoint angle and NordBord peak torque/force
Maxin (2024)	United States	PupilScreen (Apertur Inc.)	iOS	American college football	Collegiate (NCAA Division I)	Validation (diagnostic accuracy)	93	20 years (range 18–24)	100% M / 0% F	Machine-learning classification of baseline vs concussed PLR recordings (accuracy, sensitivity, specificity, AUC, F1)
Menezes-Reis (2024)	Brazil	PHAST (Physiotherapy Assessment Tool); Clinometer (v2.4)	Android	CrossFit	Mixed	Cross-sectional	22	30.6 ± 5.9 years	45% M / 55% F (10M/12F)	Functional capacity classification across 7 functional tests and injury prevalence/risk
Soga (2023)	Japan	Nordic Angle	iOS	Multiple (sports-active males)	Recreational	Validation	7	23.9 ± 2.0 years	100% M / 0% F	Break-point angle validity vs 2D motion analysis
Spork (2021)	Austria	yROM	iOS	Football (soccer)	Elite	Validation	41	14–15 years (range)	100% M / 0% F	Intra- and inter-rater reliability (ICC) of hip IR/ER/TR ROM
Van Reijen (2018)	Netherlands	Strengthen your Ankle (Versterk je enkel)	Both	Multiple (61.8% contact sports)	Mixed (active adult sports participants, 18-70 y)	RCT (economic evaluation alongside RCT)	220	37.9 ± 13.4 years	50% M / 50% F	Injury incidence density of recurrent ankle sprains leading to costs; incremental cost-effectiveness ratio (ICER)
Vriend (2015)	Netherlands	Versterk je Enkel ('Strengthen your ankle')	Both	Multiple	Mixed	Other (RE-AIM process/implementation evaluation)	25781	NR	NR	Implementation effectiveness via RE-AIM (Reach, Effectiveness, Adoption, Implementation)
Wickramagedara (2025)	Sri Lanka	SwimSight	Not reported	Swimming (freestyle,	Not reported	Validation	Not reported (2000+ videos)	NR	NR	Injury-risk classification performance (accuracy,

Study	Country	App	Platform	Sport	Competitive level	Study design	N	Age	Sex	Primary outcome(s)
				breaststroke)						recall, F1) of 1D+2D CNN ensemble
Zebis (2019)	Denmark	Based on 'Get Set—Train Smarter'	Not reported	Football, Handball	Amateur	RCT	47	APP 23.2±2.9; PHY 23.1±4.6 years	0% M / 100% F	Hamstring (semitendinosus, biceps femoris) nEMG activity during 5 exercises
**Psychological intervention (n = 5)**										
Heilmann (2023)	Germany	Fruit Ninja	Not reported	Soccer (football)	Elite (German highest youth league, Jugend A/B Bundesliga, U17–19 academy)	RCT	33	17.03 ± 0.94 years	100% M / 0% F	Executive functions — working memory (3-back), inhibition (Flanker, Cued Go/NoGo), cognitive flexibility (number-letter task)
Kuang (2025)	China	WorryTree: Anxiety CBT Therapy	Not reported	Multiple (gymnastics and martial arts noted)	Mixed (unclear — Candidate for Master of Sports category, coaches, or PE degree holders)	RCT	60	Not reported (range 23-35)	NR	Anxiety (BAI), general mental health (GHQ-28)
Mosley (2024)	United Kingdom	BreathPacer	iOS	Multiple (wheelchair racing, athletics, basketball, golf, football)	Mixed (national and international level)	Mixed methods (within-subject pre/post + focus group)	8	20.75 ± 1.38 years	~63% M / ~38% F (3 female)	Wellbeing (Outcome Rating Scale), psychological distress (PHQ-ADS), resting vmHRV (RMSSD) and Stroop performance pre vs post; athlete perceptions (focus group)
Ruud (2025)	United Kingdom	TalentCards (REBT-inspired micro-learning content)	Both	Multiple (rowing, basketball, volleyball, football, futsal, running, boxing, paddling, sailing, ultimate frisbee, weightlifting)	Mixed (club, county, national, professional, semi-professional, university)	Mixed methods (randomised comparative trial + cross-sectional survey + thematic analysis)	155 (phase-1 survey); 23 (phase-2 intervention completers)	Phase 1: 26.93 ± 11.32 y; Phase 2: 33.70 ± 14.15 y	Phase 1 ~54% M / ~45% F (~1% other/undisclosed) ; Phase 2 ~65% M / ~34% F	(1) Interrelationships/mediation among irrational performance beliefs (iPBI-2), athletic coping ability (ACSI-28) and mental health (APSQ); (2) efficacy of the 4-week REBT app on IPBs, coping and mental health (pre/post/retention)
van Iperen (2022)	Netherlands	REMBO (Running and Exercise Mental Break Optimization)	Both	Running (long-distance)	Recreational	RCT	425	44.7 years (intervention 46.2 ± 11.5; control 43.1 ± 11.8)	57.2% M / 42.8% F	Risk of running-related injuries (RRIs); chronic fatigue
**Nutrition (n = 3)**										
AlKasasbeh (2025)	Jordan	MyFitnessPal	Not reported	Mixed / various (unspecified)	Collegiate	RCT	100	Not reported (range 18–25)	~58% M / 42% F (ctrl 56/44; exp 60/40)	Sports Nutrition Knowledge (59-item); Dietary Habits (13-item)

Study	Country	App	Platform	Sport	Competitive level	Study design	N	Age	Sex	Primary outcome(s)
Kholboeva (2025)	Uzbekistan	NutriFit-AI	Not reported	Multiple (various adolescent athletic disciplines)	Not reported (adolescent athletes; competitive level unspecified)	RCT	152*	12–17*	NR	Dietary adherence, nutrition knowledge retention, and physical fitness (12-min Cooper run, vertical jump, handgrip strength); predictive-model accuracy
Simpson (2017)	New Zealand	MealLogger (MealLogger-Photo Food Journal, v4.6)	Both	Field hockey	Elite (International 94.1% / National 5.9%)	Usability (single-arm pilot feasibility study)	17	19 ± 0.7 years	100% M / 0% F	Change in nutrition knowledge (validated questionnaire, pre vs post)
**Anthropometric and maturation screening (n = 3)**										
Haynes (2025)	United States	MeThreeSixty (Size Stream)	iOS	Baseball	Collegiate (NCAA Division I)	Validation	41	20.5 ± 1.5 years	100% M / 0% F	Agreement of app-derived vs DXA body composition (BF%, FM, FFM, ALM, ALMarms, ALMlegs, LST, BMC) using existing and new equations
Shang (2025)	Portugal	Maturo	iOS	Multiple	Amateur	Validation	103	Males 11.74 ± 1.55 y; Females 13.95 ± 1.40 y (range 9-17)	73.8% M / 26.2% F	Agreement of Maturo vs expert for biological age (BA), BACA, predicted adult height (PAH), PAH%, PHV age, PHV speed
Shang (2025)	Portugal	Maturo	iOS	Basketball	Amateur	Validation	41	Boys 13.44 ± 1.32 y; Girls 13.21 ± 1.04 y (range 10-15)	43.9% M / 56.1% F	Agreement of Maturo vs expert for PHV speed and PHV age
**Tactical and match analysis (n = 2)**										
Tarnas (2023)	Poland	SoniSailing	Android	Windsurfing (RS:X class)	Mixed (regional/national juniors to national senior champions)	Usability (questionnaire-based opinion evaluation)	13	21.4 ± 4.3 years	46% M / 54% F (6 M / 7 F)	Perceived usefulness of the real-time auditory feedback (8-item questionnaire, 5-point Likert) across technical and decision-support aspects
Verlin (2019)	United States	PoloTrac	iOS	Water polo	Mixed (designed for amateur to professional; usability testers from local water polo community)	Usability	5	NR	NR	Usability and perceived usefulness of the app interface

Table S3. Methodological quality appraisal of included studies using the Mixed Methods Appraisal Tool (MMAT, version 2018).

MMAT = Mixed Methods Appraisal Tool (version 2018; Hong et al., 2018). Y = Yes; N = No; CT = Can't tell. Ratings are shaded green (Y), red (N), and amber (CT) for readability.

S1 and S2 are the two screening questions applied to every study: S1, Are there clear research questions? S2, Do the collected data allow the research questions to be addressed?

Criteria C1–C5 are category-specific, as follows.

Randomized controlled trials (Category 2): C1, Is randomization appropriately performed? C2, Are the groups comparable at baseline? C3, Are there complete outcome data? C4, Are outcome assessors blind to the intervention provided? C5, Did the participants adhere to the assigned intervention?

Quantitative non-randomized (Category 3): C1, Are the participants representative of the target population? C2, Are measurements appropriate regarding both the outcome and the exposure? C3, Are there complete outcome data? C4, Are the confounders accounted for in the design and analysis? C5, During the study period, did the exposure occur as intended?

Quantitative descriptive (Category 4): C1, Is the sampling strategy relevant to the research question? C2, Is the sample representative of the target population? C3, Are the measurements appropriate? C4, Is the risk of nonresponse bias low? C5, Is the statistical analysis appropriate to answer the research question?

Following the developers' guidance, no overall quality score was computed; ratings are presented per criterion. The appraisal characterizes the methodological strengths and limitations of the evidence base and was not used to weight or exclude studies.

Table S3a. Frequency of MMAT criterion ratings across included studies.

S1 and S2 are reported across all 111 included studies, since these screening questions apply uniformly regardless of design. C1–C5 are reported only for studies coded as MMAT Category 4 (quantitative descriptive; n = 82), the dominant design bucket in this review, because criterion definitions differ across MMAT design categories and are not directly comparable. Percentages are rounded to the nearest whole number.

Criterion	n	Yes	No	Can't tell	% No
S1 — Clear research questions (all designs)	111	96	0	15	0%
S2 — Data allow research questions to be addressed (all designs)	111	93	0	18	0%
Category-specific criteria — Quantitative descriptive studies only (MMAT Category 4, n = 82)					
C1 — Sampling strategy relevant to the research question	82	63	2	17	2%
C2 — Sample representative of the target population	82	4	65	13	79%
C3 — Measurements appropriate	82	61	1	20	1%
C4 — Low risk of nonresponse bias	82	15	11	56	13%
C5 — Statistical analysis appropriate	82	59	15	8	18%

Table S3b. Per-study MMAT ratings.

Study (first author, year)	Design (MMAT category)	S1	S2	C1	C2	C3	C4	C5	Notes / justification
AlKasasbeh et al., 2025	RCT (Cat. 2)	Y	Y	CT	Y	CT	N	Y	Parallel-group RCT (n = 100; MyFitnessPal vs. lecture). Randomization stated but sequence generation and allocation concealment not described (C1); groups balanced at baseline per Table 1 (C2); non-adherent participants excluded with no attrition flow/CONSORT diagram (C3); outcomes self-reported with no assessor blinding (C4); app use monitored with weekly reminders and attendance recorded (C5).
Attigala et al., 2019	Quantitative descriptive (Cat. 4)	CT	CT	CT	N	CT	CT	N	Prototype system paper (Optio) tested on one coach and 50 badminton trainees. No explicit research question/hypothesis and testing largely anecdotal (S1, S2); convenience single-coach sample, badminton only (C1, C2); weight model (RMSE 6.08) and stress classifier (AUC 0.72) reported, but ground truth and validation design underspecified (C3); nonresponse not addressed (C4); analysis minimal relative to the claims made (C5).
Alexandersen et al., 2025	Quantitative non-randomized (Cat. 3)	Y	Y	Y	CT	CT	CT	CT	Observational longitudinal association study (65 elite female players; linear mixed models). Clear question with adequate data (S1, S2); cohort representative of the target league (C1); exposure measured with a reduced/adapted Hooper index and outcome via the proprietary, undisclosed InStat Index (C2); substantial missing matchday data and exclusion of players without InStat scores (C3); player/team clustering modeled but tactical and fitness confounders unmeasured (C4); timestamp limitation means 'pre-match' exposure is not guaranteed (C5).
Aranki et al., 2017	Quantitative descriptive (Cat. 4)	Y	CT	CT	N	Y	CT	CT	Ongoing feasibility study (6 subjects) reported qualitatively through case studies. Objectives clear (S1) but study incomplete and post-study survey not yet administered (S2); convenience sample appropriate for a feasibility aim but small (C1, C2); cadence/speed/HR derived from verifiable, literature-based algorithms (C3); nonresponse not evaluated (C4); no formal statistical analysis, explicitly deferred to future work (C5).
Aranki et al., 2018	Quantitative	Y	Y	Y	N	Y	CT	Y	Completed feasibility/usability study (6 subjects, 22 runs) extending the 2017 conference paper. Clear objectives with data addressing them (S1,

Study (first author, year)	Design (MMAT category)	S1	S2	C1	C2	C3	C4	C5	Notes / justification
	descriptive (Cat. 4)								S2); convenience sampling relevant to a feasibility aim (C1) though the sample is small and nonrepresentative (C2); measurements appropriate, including a validated (ROF-based) fatigue scale and structured acceptability/privacy questionnaires (C3); small N with no nonresponse analysis (C4); descriptive analysis appropriate for the feasibility aim (C5).
Stafylidis et al., 2025	Quantitative descriptive (Cat. 4)	Y	Y	Y	N	Y	CT	Y	Concurrent-validity and intra-rater reliability study (n = 21 male youth soccer players, U14-U19) comparing MyJump 2 with the Chronojump contact mat. Clear aims and simultaneous dual-device data address them (S1, S2); sampling relevant to the validation aim, drawing from five age groups (C1) but a small single-team convenience sample not representative of the broader youth-athlete population (C2); measurements appropriate, with an a priori power analysis, standardized 240 Hz setup and reanalysis blinded to original values (C3); reliability was intra-rater single-session with no inter-rater assessment and Chronojump (not a force plate) as reference, but nonresponse is not a threat given complete simultaneous recording (C4, treated as CT); Bland-Altman, correlation and regression analyses appropriate to the aim (C5).
Pereira et al., 2020	Quantitative descriptive (Cat. 4)	Y	Y	Y	N	Y	CT	Y	Concurrent-validity study (n = 31 male recreational runners) comparing the HRV Expert/CardioMood app plus Polar H10 strap against ECG for R-R interval and HRV indices in supine and sitting positions. Clear aim with simultaneously recorded data addressing it (S1, S2); sampling appropriate to the validation aim (C1) but a single-sex convenience sample limits representativeness (C2); measurements appropriate, using standardized simultaneous ECG acquisition and Kubios processing (C3); complete simultaneous recording means nonresponse is not a real threat, though not explicitly discussed (C4, CT); t-tests, Pearson correlation, regression/SEE and Bland-Altman are appropriate (C5).
Uysal et al., 2023	Quantitative descriptive (Cat. 4)	Y	Y	Y	N	Y	CT	Y	Concurrent-validity, reliability and sensitivity study (n = 30 college team-sport athletes) comparing three mobile apps and two analog stopwatches against timing gates on the Illinois change-of-direction test. Clear aim with simultaneous six-device data addressing it (S1, S2); simple random sampling relevant to the aim (C1) but a modest, mostly male, mixed-sport convenience sample not broadly representative (C2); measurements appropriate, with an a priori sample-size calculation, gold-standard timing gates and standardized protocol (C3); only two trials per athlete with complete simultaneous data, so nonresponse is not addressed but is a limited threat (C4, CT); Pearson correlation, regression, Bland-Altman, ICC/CV and TE/SWC analyses are appropriate (C5).
Babouras et al., 2024	Quantitative descriptive (Cat. 4)	Y	Y	Y	N	Y	CT	Y	Device-validation study (254 varsity athletes; MediaPipe smartphone app vs. Kinect V2 as ground truth across drop vertical jump, single-leg squat and single-leg hop). Clear aim with adequate paired-angle data (S1, S2); large multi-sport convenience sample suits the validation aim (C1) but comprises only elite varsity athletes with an unequal sex distribution, which the authors note limits generalization to the general population (C2). Kinect V2 is an imperfect criterion rather than a laboratory gold standard, but keyframe-detection algorithms were validated (ICC > 0.9) and angle formulas defined (C3); 46 trials excluded for recording faults with no formal nonresponse analysis (C4); analysis reports mean absolute angle error with 95% CI, RMSE and t-tests, adequate for quantifying agreement though lacking ICC/Bland–Altman (C5).
Balsalobre-Fernández, 2026	Quantitative descriptive (Cat. 4)	Y	Y	Y	N	Y	CT	Y	Repeated-measures validation study (17 physically active participants; 204 drop jumps; My Jump Lab app vs. dual force-platform criterion). Clear aim with adequate paired data (S1, S2); convenience sample of recreationally active sports-science students fits the validation aim (C1) but is not representative of elite or clinical populations, as the author acknowledges (C2). Force platform at 1000 Hz and 240 fps video with standard procedures are appropriate measurements (C3); complete data across both sessions but no nonresponse analysis (C4); comprehensive, appropriate statistics — Pearson r, ICC, CCC, Bland–Altman, RMSE and CV (C5).
Balsalobre-Fernández et al., 2019	Quantitative descriptive (Cat. 4)	Y	Y	Y	N	Y	CT	Y	Concurrent validity/reliability study (20 adolescent soccer players; 120 change-of-direction times; CODTimer app vs. Witty timing-gate criterion). Clear aim with adequate data (S1, S2); homogeneous convenience sample suits the validation aim (C1) but a single narrow group of adolescent soccer players is not representative of the broader target population (C2). Gold-standard timing gates, 240 fps capture and trained observers are appropriate (C3); all trials analysed with no nonresponse analysis (C4); appropriate statistics — linear regression, ICC, CV, Bland–Altman and effect sizes (C5).
Balsalobre-Fernández et al., 2017	Quantitative descriptive (Cat. 4)	Y	Y	Y	N	Y	CT	Y	Technical validation study (only 2 treadmill runners; 96 steps across six speeds; Runmatic app vs. Optojump Next criterion). Clear aim with adequate step-level data, the authors justifying the small n because the time computations are subject-independent (S1, S2, C1); with only two forefoot-strike male runners the sample is clearly not representative (C2). Optojump at 1000 Hz and 240 fps video with validated Morin equations are appropriate measurements (C3); all steps analysed with no nonresponse analysis (C4); appropriate statistics — Pearson r, ICC, Cronbach's alpha, CV, t-test/ANOVA — which detected a velocity-dependent aerial-time bias (C5).
Balsalobre-Fernández et al., 2020	Quantitative descriptive (Cat. 4)	Y	Y	Y	N	Y	CT	Y	Validation study (10 NCAA Division I weightlifters; 100 snatch repetitions; computer-vision My Lift app vs. Vicon motion-capture criterion). Clear aim with adequate paired data (S1, S2); small experienced-athlete convenience sample suits the validation aim (C1) but is not representative of the broader population (C2). 14-camera Vicon system, sagittal video and Butterworth-filtered processing are appropriate measurements (C3); all reps analysed, though short inter-rep rest may have affected performance and no nonresponse analysis was done (C4); appropriate statistics — Pearson r, ICC, Bland–Altman, cross-correlation and effect sizes (C5).
Balsalobre-Fernández et al., 2018	Quantitative descriptive (Cat. 4)	Y	Y	Y	N	Y	CT	Y	Validation study (10 experienced resistance-trained males; 130 bench-press repetitions; PowerLift app vs. Smartcoach linear-transducer criterion, plus load–velocity 1RM estimation). Clear aim with adequate data (S1, S2); small convenience sample of expert lifters fits the validation aim (C1) but is not representative of the broader target population (C2). Linear transducer at 1000 Hz, 240 fps video and two independent observers give appropriate measurements (C3); complete data with no nonresponse analysis (C4); appropriate statistics — bootstrapped Pearson r, ICC, Bland–Altman and Wilcoxon for 1RM (C5).

Study (first author, year)	Design (MMAT category)	S1	S2	C1	C2	C3	C4	C5	Notes / justification
Bertschy et al., 2023	Quantitative non-randomized (Cat. 3)	Y	Y	CT	Y	Y	Y	Y	Prospective single-team cohort (23 Division I female soccer players, 33 sessions, 646 observations; GEE of wellness on RPE). Clear aims and hypotheses with data addressing them (S1, S2); one convenience-sampled team of unclear representativeness of collegiate female soccer (C1); app-based wellness scales grounded in prior work and GPS-derived load appropriate (C2); ~13.5% missing data fully accounted for in a flow diagram (C3); models adjust for training load and within-player correlation (C4); daily surveys completed as intended under staff monitoring (C5).
Bilal & Prasetyo, 2025	Quantitative descriptive (Cat. 4)	Y	Y	Y	N	Y	CT	Y	Prototype evaluation of an IMU + GPT coaching system (25 amateur cue-sports players, two weeks). Evaluation aims stated with data addressing them (S1, S2); amateur convenience sample fits the feasibility/usability aim (C1) but the authors concede the small homogeneous group is not representative of the wider target population (C2); validated SUS plus objective latency/motion metrics and an expert-coach correlation (C3); nonresponse/dropout not reported (C4); descriptive statistics and Spearman correlation appropriate to the aims (C5).
Bilić et al., 2023	Quantitative descriptive (Cat. 4)	Y	Y	Y	N	Y	Y	CT	Concurrent-validity study of the SwingVision app against radar and reference video (5 elite male junior players; 300 serves, 500 rally shots). Shot-level sampling volume is relevant to the validity aim (C1), but five homogeneous right-handed elite juniors do not represent the app's broad target population (C2); reference standards (Stalker radar, reference camera, clay-court ball marks) appropriate (C3); all recruited players completed all trials (C4); agreement expressed as average inter-item correlations labelled ICC, applied even to zone frequencies, with no Bland-Altman or per-shot agreement analysis (C5).
Bishop et al., 2022	Quantitative descriptive (Cat. 4)	Y	Y	Y	CT	Y	Y	Y	Test-retest measurement study of app-derived jump/CODS asymmetry (30 national-level youth basketball players, two sessions). Clear aims with data addressing them (S1, S2); a priori power analysis and inclusion criteria make sampling relevant (C1), though the single-country convenience sample leaves broader representativeness unclear (C2); My Jump 2 and CODTimer have prior validity evidence and protocols were standardized (C3); all 30 athletes contributed to both sessions with no reported dropout (C4); ICC, CV, Kappa, and Bonferroni-corrected Spearman analyses appropriate (C5).
Boratto et al., 2017	Quantitative non-randomized (Cat. 3)	Y	Y	CT	Y	Y	N	Y	Observational analysis of 103 paying subscribers of a commercial e-coaching platform (1,601 sessions) comparing trainer-created, user-created, and free workouts. Three explicit research questions answered by platform data (S1, S2); self-selected purchasers with short-term subscribers excluded, so representativeness of the broader runner/app-user population is unclear (C1); exposure and outcomes objectively logged by the platform (C2, C3) and exposure occurred as recorded (C5); ANOVA/t-tests include no adjustment for confounders such as baseline motivation, which the authors acknowledge via the Hawthorne effect (C4).
Cabahug et al., 2025	Quantitative descriptive (Cat. 4)	CT	CT	CT	N	CT	CT	N	System-demonstration paper of a smartphone pose-estimation app tested on a single gymnast performing one giant-swing routine. No stated research question and testing largely anecdotal (S1, S2); no sampling strategy described (C1) and one gymnast cannot represent the target population (C2); pose-detection accuracy verified only by informal frame-by-frame visual inspection with no quantitative ground truth (C3); nonresponse not applicable/addressed (C4); no statistical analysis performed (C5).
Cetin & Isik, 2021	Quantitative descriptive (Cat. 4)	Y	Y	Y	N	Y	CT	Y	Validity/reliability study of the My Lift app for estimating 1RM in conventional deadlift and back squat versus the actual ACSM 1RM protocol (n = 10 male youth weightlifters; repeated trials 48 h apart). Clear aim with data that address it (S1, S2); recruiting competitive weightlifters is relevant to the 1RM aim despite the small n (C1), but a single-club, young, all-male sample is not representative (C2); direct actual-1RM criterion with standardized app procedures (C3); attrition/nonresponse not reported (C4); paired t-tests, ICC, and Bland–Altman appropriate for the aim, though precision is limited by n = 10 (C5).
Çetin et al., 2024	Quantitative descriptive (Cat. 4)	Y	Y	Y	N	Y	CT	Y	Concurrent validity/reliability study of the Jump Power app versus the Optojump photoelectric system for SJ and CMJ (n = 22 male U21 professional soccer players; three sessions 48 h apart). Clear aims with adequate data (S1, S2); sampling relevant to the validation aim with explicit inclusion criteria (C1), but a single male U21 squad is not representative, as the authors acknowledge (C2); simultaneous measurement against a validated criterion under standardized conditions (C3); missing data/attrition not reported (C4); CV, equivalence t-tests, Bland–Altman, and MAPE analyses appropriate (C5).
Chen et al., 2021	Quantitative descriptive (Cat. 4)	Y	Y	Y	CT	Y	Y	Y	Concurrent validity/reliability study of the COD timer app and stopwatches versus 1000-Hz error-corrected timing gates in the 505 test (n = 62 youth basketball players; 367 valid trials). Clear question supported by an a priori power analysis (S1, S2, C1); representativeness beyond youth basketball players from one setting is unclear (C2); simultaneous timing against a gold-standard criterion with standardized procedures (C3); only 5 of 372 trials excluded, with reasons reported (C4); comprehensive and appropriate validity/reliability statistics (C5).
Chiu et al., 2020	Quantitative descriptive (Cat. 4)	Y	Y	Y	N	Y	CT	Y	Feasibility study of a smartphone accelerometer system for badminton six-point footwork (n = 30 university badminton players; fast vs. slow groups by median split). Clear purpose with data that address it (S1, S2); sampling relevant to the feasibility aim with stated inclusion criteria (C1), but a young, university-based sample the authors acknowledge limits generalizability (C2); standardized phone placement and literature-supported accelerometer measures, although overall time relied on a stopwatch (C3); nonresponse/data completeness not reported (C4); independent t-tests with effect sizes and normality testing appropriate (C5).
Cirilo et al., 2021	Quantitative descriptive (Cat. 4)	CT	CT	CT	N	CT	CT	N	Prototype/system-demonstration paper for the ABT boxing app, tested with 1 professional and 10 amateur boxers in Lima. No explicit research question or hypothesis and validation largely anecdotal (S1, S2); convenience sample with no recruitment description (C1) and not representative of the target boxing population (C2); the claimed 95% punch-detection accuracy has no criterion measure or protocol detail, and usability was an ad hoc Likert survey (C3); nonresponse not addressed (C4); analysis limited to descriptive averages with no statistical testing (C5).

Study (first author, year)	Design (MMAT category)	S1	S2	C1	C2	C3	C4	C5	Notes / justification
Driller et al., 2017	Quantitative descriptive (Cat. 4)	Y	Y	Y	CT	Y	CT	Y	Independent validity study of the My Jump app versus force plates for CMJ height and flight time (n = 61 recreational athletes; 183 jumps; inter-scorer reliability on 50 jumps). Clear aim with data that address it (S1, S2); deliberate recruitment across sexes and ability levels is relevant to validating across jump heights (C1), though recruitment source and representativeness are not described (C2); simultaneous comparison against the gold-standard force plate using a matched flight-time equation and 240-Hz camera (C3); missing data not reported (C4); correlations, limits of agreement, TEE, and heteroskedasticity testing appropriate (C5).
Dias et al., 2025	Quantitative descriptive (Cat. 4)	Y	Y	Y	N	Y	CT	Y	Concurrent-validity and reliability study of two smartphone apps (My Jump Lab, VertVision) versus a Chronojump contact mat for CMJ height (n = 27 amateur handball players; 5 jumps each; four independent observers). Clear aim with data that address it (S1, S2); athletes recruited to meet a sample-size calculation, relevant to the validation aim (C1), but the sample is a small single-region convenience group of amateur handball players and not representative of a broader target population (C2); simultaneous comparison against a validated 1000-Hz contact mat with a 240-fps camera and a common takeoff/landing criterion (C3); no attrition or nonresponse account (C4); ICC, CV, SEM, Pearson correlations and Bland-Altman analyses appropriate (C5).
de Oliveira et al., 2024	Quantitative descriptive (Cat. 4)	Y	Y	Y	N	Y	CT	Y	Criterion-validity study of a boxing-specific incremental test (TBOX, ITStriker app) against an incremental treadmill test in the same athletes (n = 10 male boxers; 3 professional, 7 amateur). Clear aim with data that address it (S1, S2); purposive recruitment against defined competition/training criteria suits the validation aim (C1), but the sample is small, male-only and homogeneous and not representative of the broader boxer population, as the authors note (C2); HR via chest strap with HRDP determined by the DMAX method and a treadmill reference are appropriate measures (C3); all ten completed but no nonresponse analysis (C4); paired t-tests, Pearson correlation, CV and Bland-Altman limits of agreement appropriate (C5).
Muslimin et al., 2024	Quantitative descriptive (Cat. 4)	Y	CT	Y	N	CT	CT	N	Research-and-development study of an Android serve/smash skill-test instrument with expert validation, small- (n = 12) and large-scale (n = 24) eligibility trials and a manual-versus-digital effectiveness test (n = 52). Aim to develop and evaluate the tool is stated (S1), but the effectiveness data are reported inconsistently, so it is unclear they address the question (S2): the paired-samples t is given as both -28.284 and -11.406, df = 50 with n = 52, and the normality threshold is misstated as p > 0.005. Purposive recruitment of trained volleyball players fits the eligibility aim (C1), though the two-school sample (age 14-20) is narrow and not representative (C2); the scoring instrument's own validity/reliability rests on evaluator Likert ratings and is underspecified (C3); nonresponse not addressed (C4); the contradictory test statistics and misreported thresholds make the statistical analysis unreliable (C5).
Duignan et al., 2019	Qualitative (Cat. 1)	Y	Y	Y	Y	Y	Y	Y	Qualitative interview study of stakeholder perceptions of implementing a mobile athlete self-report measure in elite Gaelic Games (n = 21; 10 players, 11 coaches/support staff across 15 teams; semi-structured interviews; NVivo thematic analysis). Clear aim with data that address it (S1, S2); semi-structured interviewing is appropriate for eliciting implementation perceptions (C1) and the topic guide and sampling adequately address the question (C2); four higher-order themes are grounded in the data with supporting quotations (C3); interpretations are substantiated by participant quotes and linked to prior literature (C4); dual-coder analysis, an insider/outsider reflexivity account and audit of the topic guide give coherence across data, collection, analysis and interpretation (C5).
Dupuit et al., 2023	Quantitative descriptive (Cat. 4)	Y	Y	Y	N	Y	CT	Y	Feasibility/methodological study of an online calendar app to monitor menstrual status and establish individual menstrual profiles in elite female soccer players over seven months (n = 19; 14 natural-cycle, 5 oral-contraceptive users). Clear aim with adherence, compliance and phase-determination data that address it (S1, S2); the whole team volunteering (19 of 21) suits the on-field feasibility aim (C1), but a single French second-division club is not representative of elite female athletes more broadly (C2); a daily app diary, calendar-based phase determination and month-by-month adherence/compliance metrics are appropriate for the aim, with the calendar method's limits acknowledged (C3); adherence is well documented and injury dropouts are described, but risk of nonresponse bias on the descriptive profiles is not formally established, and missing entries cluster in December and late season (C4); t-tests, ANOVA and Pearson correlations are appropriate (C5).
Flatt et al., 2021	Quantitative non-randomized (Cat. 3)	Y	Y	CT	Y	CT	CT	Y	Prospective observational cohort tracking heart-rate variability across a season and testing a position (linemen vs. nonlinemen) x time association via linear mixed models (n = 20 analyzed of 32; 8 linemen, 12 nonlinemen). Clear hypothesis with longitudinal data that address it (S1, S2); the cohort is a single national-champion program and 12 of 32 players were excluded (9 for insufficient measures, 3 for injury), limiting representativeness (C1); HRV via a smartphone PPG tool validated against ECG, with clearly defined position exposure and Player Load, are appropriate measures (C2); those exclusions plus omitted post-match and strength-session days leave outcome data incomplete despite per-timepoint averaging (C3); player-level random effects and body-mass analysis are used, but key confounders (aerobic fitness, sleep, diet, academic/competitive stress) are unmeasured and only acknowledged (C4); the season-long position exposure clearly occurred as intended (C5).
Flatt & Esco, 2015	Quantitative non-randomized (Cat. 3)	Y	Y	CT	Y	CT	CT	Y	Single-group observational repeated-measures study (n = 9 female college soccer players; supine and standing ultra-short lnRMSSDx20 via the athlete app across moderate-, high-, and low-load weeks), analysed as a training-load-to-HRV association with no randomization, so Cat. 3, while also carrying a 7- vs. 5-/3-day agreement aim. Clear question with data addressing it (S1, S2); small single-team convenience cohort limits representativeness of female team-sport athletes (C1); the HRV tool was cross-validated against ECG and load quantified by sRPE, appropriate exposure and outcome measures (C2). Two of 11 recruited were excluded for incomplete self-recordings with no full attrition/compliance account (C3); the within-subject design controls individual factors but confounders are unmeasured and week order is not counterbalanced (C4); training load varied as intended, confirmed by significantly different weekly sRPE (C5).
Ha et al., 2011	Quantitative non-	Y	Y	CT	CT	CT	N	CT	Cross-sectional survey with multiple-regression on predictors (n = 196 Taekwondo trainees, Gwangju), relating perceived 'entertaining' and

Study (first author, year)	Design (MMAT category)	S1	S2	C1	C2	C3	C4	C5	Notes / justification
	randomized (Cat. 3)								'useful' app factors to education, reregistration and recommendation outcomes; regression on predictors places it in Cat. 3 rather than a purely descriptive survey. Analytic questions and data are stated (S1, S2); 'random sampling' claimed but limited to one city with the frame underspecified (C1); self-report questionnaire with no reported reliability/validity and common-method concerns (C2); no account of item missingness or response rate (C3); regressions include only the two app factors with demographics collected but not adjusted as confounders (C4); actual app-use fidelity/exposure not verified (C5).
Flatt et al., 2017	Quantitative non-randomized (Cat. 3)	Y	Y	CT	Y	Y	CT	Y	Single-group observational repeated-measures study (n = 10 Division-1 sprint-swimmers; daily lnRMSSD and wellness via a smartphone app across 1 baseline, 2 overload and 2 taper weeks), analysed as a training-phase-to-HRV/wellness association with no randomization, so Cat. 3. Clear aims and hypotheses with data addressing them (S1, S2); convenience single mixed-sex squad limits representativeness (C1); a PPG-based app validated against ECG, the McLean-adapted wellness scale and external load are appropriate measures (C2); all 10 were analysed with week 4 omitted by justified design and no dropout, giving complete outcome data (C3). The within-subject design controls individual factors but confounders are unmeasured and phase order is fixed by periodization (C4); the overload and taper occurred as planned, verified by external load data (C5).
Gallardo-Fuentes et al., 2016	Quantitative descriptive (Cat. 4)	Y	Y	Y	N	Y	CT	Y	Instrument reliability/validity study (n = 21 trained male and female track-and-field athletes; 630 SJ/CMJ/DJ jumps over 2 days) comparing the My Jump app against a contact platform; single-group validation confirms Cat. 4. Clear aim with adequate paired data (S1, S2); convenience sample of competitive athletes suits the validation aim (C1) but is small and single-discipline, not representative of the broader athlete population (C2); criterion contact platform, standardized jump protocols and a validated flight-time equation are appropriate (C3); all participants completed both sessions but recruitment/response is not described and nonresponse is not formally evaluated (C4); ICC, Bland-Altman, Pearson r, Cronbach's alpha and CV are appropriate (C5).
Hadza et al., 2026	Quantitative descriptive (Cat. 4)	Y	Y	Y	N	Y	CT	Y	Intra- and inter-rater reliability study (n = 12 international-level female kata karatekas; four techniques timed with MyJump2 by an experienced and an inexperienced rater on two occasions, 60 attempts per test); single-group reliability confirms Cat. 4. Explicit research questions with data that address them (S1, S2); purposive elite sample with stated inclusion criteria and an a priori power estimate suits the aim (C1) but is small, all-female and technique-specific, not representative broadly (C2); 240-Hz frame-by-frame timing with defined initiation/contact frames is appropriate (C3); complete data with repeated failed trials but no nonresponse analysis (C4); ICC (Koo & Li model), Cronbach's alpha, SEM, SWC and Bland-Altman are appropriate (C5).
Kholboeva et al., 2025	RCT (Cat. 2)	Y	CT	CT	Y	CT	N	Y	Stated as a randomized controlled trial comparing the NutriFit-AI app with conventional nutrition education (and MyFitnessPal) in adolescent athletes, so appraised as Cat. 2. Aim/hypothesis is clear (S1), but sample sizes are irreconcilable across the paper (120 vs. 200 enrolled; 152 'analytic cohort' at a claimed 94% retention), so it cannot be confirmed that the reported data coherently address the questions (S2). Randomization asserted but with no sequence-generation or allocation-concealment method described (C1); baseline comparability across groups is explicitly reported (C2); attrition is claimed but the conflicting Ns give no coherent flow/CONSORT account (C3); no assessor blinding with largely self-reported/in-app adherence and knowledge outcomes, asymmetric across arms (C4); adherence and engagement to the intervention are documented (C5).
Ruud & Jooste, 2025	Mixed methods (Cat. 5)	Y	Y	CT	CT	CT	N	CT	Multiphase study combining a cross-sectional mediation survey (n = 155), a randomized REBT-vs-PST mobile-app intervention (n ≈ 23; retention attrition to n = 15 at three-week follow-up), and a formal reflexive thematic analysis of open-ended user feedback (Braun & Clarke); appraised as mixed methods because a stated qualitative method is integrated with the quantitative arms. Questions/hypotheses are clear and the data address them (S1, S2); no explicit rationale for a mixed methods design is articulated (C1) and the quantitative and qualitative strands are reported largely in parallel with limited formal integration and interpretation of integrated outputs (C2, C3); the largely null quantitative effects on coping ability and mental health versus uniformly positive qualitative feedback are a divergence that is not explicitly reconciled (C4); qualitative rigor (peer debriefing, verbatim quotes) is reasonable but the small, attrition-affected RCT arm weakens cross-tradition quality (C5). (If the qualitative arm is treated as supplementary, this appraises as an RCT, Cat. 2: Y Y CT Y N N CT.)
Haynes et al., 2025	Quantitative descriptive (Cat. 4)	Y	Y	Y	N	Y	CT	Y	Single-group method-agreement and prediction-model-development study (n = 41 Division I baseball athletes; testing subset n = 7) comparing smartphone anthropometry against DXA and deriving new LASSO equations, with no comparison group. Aim/hypotheses are clear and the data address them (S1, S2); sampling fits the athlete-specific aim (C1), but the sample is almost entirely non-Hispanic White males from one program and is not representative of DI baseball broadly (C2); the DXA criterion and validated app measurements and the statistical approach (LASSO, Bland–Altman, Deming, equivalence tests, CCC, cross-validation) are appropriate (C3, C5); the recruitment/participation fraction is not reported, so nonresponse risk cannot be judged (C4).
Heilmann et al., 2023	RCT (Cat. 2)	Y	Y	Y	N	CT	CT	CT	Randomized controlled trial (N = 33; intervention n = 15 vs. passive control n = 18) of an eight-week smartphone-game (Fruit Ninja) cognitive-training intervention with computerized executive-function outcomes. Aim/hypotheses clear and data address them (S1, S2); randomization used Research Randomizer and is described (C1), but the groups differed significantly at baseline on key EF outcomes (vertical-cued Go/NoGo and incongruent Flanker response times), so baseline comparability fails (C2); reduced and variable degrees of freedom across EF tasks indicate unexplained missing outcome data (C3); assessor blinding is not reported for the self-administered computer tasks in an unblinded passive-control design (C4); intervention adherence (game play) was explicitly not monitored, so compliance cannot be verified (C5).
Iliadis et al., 2021	Quantitative	Y	Y	Y	N	Y	Y	CT	Single-group feasibility/evaluation study (n = 16 elite male cyclists) assessing whether a novel mHealth telemetry system detects exercise-

Study (first author, year)	Design (MMAT category)	S1	S2	C1	C2	C3	C4	C5	Notes / justification
	descriptive (Cat. 4)								related disorders, correlating reported symptoms with monitoring findings across two training periods. Objective is clearly stated and the data address it (S1, S2); convenience sampling suits the elite-cyclist aim (C1), but 16 high-level males from one region are not representative of the broader target population (C2); physiological measurements and instruments are appropriate (C3) and all 16 completed both testing periods, keeping nonresponse low (C4); the central symptom–finding correlation is asserted but not clearly quantified (small n; mixed use of paired t-tests and chi-square), so statistical appropriateness for that key inference is uncertain (C5).
Holmes et al., 2020	Quantitative descriptive (Cat. 4)	Y	Y	Y	N	Y	N	Y	Observational descriptive study of daily HRV self-measurement compliance over 17 weeks in two convenience teams (16 male hockey, 47 female rowers). Question and hypothesis (≥80% compliance) are clear and the data address them (S1, S2); sampling fits the compliance aim (C1); the two mismatched teams (different sport, sex, app, and competition level) are not representative of collegiate athletes generally (C2); the compliance operationalization and instruments are appropriate (C3) and the descriptive analysis suits the aim (C5), albeit with minor numeric inconsistencies between abstract and results; because the outcome is compliance itself and there was heavy attrition/exclusion (23→16 hockey; 65→47 rowers; weekly compliance as low as ~9%), nonresponse bias is high (C4).
Hsu et al., 2025	RCT (Cat. 2)	Y	Y	CT	Y	CT	N	Y	App-development paper whose educational-effectiveness component is a parallel-group RCT (n = 143; 71 control / 67 experimental analyzed) comparing ABCapp-assisted badminton teaching against traditional instruction. Clear aim with pre/post clear-distance tests, expert scoring, and a self-perception questionnaire (S1, S2); students randomized by class with no sequence-generation or allocation-concealment detail (C1); groups equivalent at baseline (clear-distance p = 0.5200) (C2); minor attrition (74→71; 69→67) for injury/absence noted but no CONSORT flow (C3); clear-distance and expert scoring performed by unblinded raters and self-perception self-reported (C4); intervention adherence structured over weeks 2–9 with supervised app use (C5).
Johansen et al., 2020	Quantitative descriptive (Cat. 4)	CT	CT	CT	N	Y	CT	N	System-description paper for the PMSys athlete-monitoring app with an exploratory LSTM prediction experiment (single Norwegian elite team, 19 players). Aim framed loosely as evaluating whether ML can predict future states, with no formal research question or hypothesis and authors noting data were not expected to suffice (S1, S2); convenience single-team sample judged against that exploratory aim (C1) is not representative of athletes generally (C2); PMSys uses validated self-report instruments (sRPE, wellness), so measurements are appropriate (C3); reporting compliance/missing data not addressed (C4); prediction assessed only by visual comparison and a correlation matrix with no formal metrics, i.e. anecdotal analysis (C5).
Johansson et al., 2026	Quantitative descriptive (Cat. 4)	Y	Y	Y	Y	Y	Y	Y	Intra-session reliability and concurrent-validity study of a smartphone PPG app (CameraHRV) and a Polar H10 chest strap against ECG in 37 trained athletes, each measured twice. Explicit objective addressed by concurrent recordings and appropriate agreement statistics (S1, S2); an a priori sample-size calculation (target ICC 0.83; minimum 34) supports a sampling strategy relevant to the validation aim (C1); the trained/athletic sample reflects the stated target population (C2); gold-standard ECG reference, a validated chest strap, and standardized short-recording protocol with suitable HRV metrics (RMSSD, pNN50) make measurements appropriate (C3); one participant with missing repeat data was excluded (36 reliability / 37 validity), a negligible and accounted-for loss (C4); ICC, CV%, MAPE, and Bland–Altman analyses are appropriate to the question (C5).
Janssen et al., 2020	Quantitative descriptive (Cat. 4)	Y	Y	CT	N	CT	N	Y	End-user experience study of the Inspirun e-Coach running app, following 19 runners through a 20-session automatically adapted training scheme with repeated Likert questionnaires (T1–T6). Clear questions on experienced personalization and motivation addressed by the longitudinal survey and app data (S1, S2); self-selected social-media convenience sample judged against the experience aim (C1) is small, highly educated, and contains no beginners, so it is not representative of the broad novice-runner target (C2); constructs measured with single newly-devised Likert items alongside validated RPE (C3); heavy attrition (43 volunteered → 19 completed; T6 n = 16) is unanalyzed, so nonresponse bias is a concern (C4); descriptive statistics, Friedman tests, and Spearman correlations suit the aim and sample (C5).
Lopez & Banchero, 2026	Quantitative descriptive (Cat. 4)	CT	CT	CT	N	Y	CT	N	Implementation paper describing a real-time smartphone application for audio-based vertical-jump-height measurement, whose accuracy evidence is largely carried over from the authors' prior studies rather than newly collected here. No formal research question or hypothesis is posed for a new study, and the reported detection/accuracy results derive from earlier work (S1, S2); the validation dataset (>300 jumps) and its participants come from references and are not characterized here, and cross-device testing is described without sampling detail (C1, C2); where reported, commercial jump-mat criterion measures and sound CNN methods make the measurements appropriate (C3); nonresponse/attrition is not addressed (C4); the paper's own cross-device validation is reported only qualitatively ("highly positive," "consistent and reliable") without statistics (C5).
Lebedev et al., 2021	Quantitative non-randomized (Cat. 3)	Y	Y	CT	Y	CT	N	Y	Prospective two-group cohort study (n = 400: 200 professionally training athletes vs. 200 non-sport controls) justifying a two-component remote-monitoring method by comparing physical and psycho-emotional indices across non-stressful and stressful periods. Clear question with data that address it via between-group index comparisons (S1, S2); both groups are specific student convenience samples, so representativeness of young athletes generally is partial (C1); validated adaptive-potential (Baevsky, Kerdo) and standard psychometric stress instruments, with a documented ≥1-year exposure definition, are appropriate (C2); n = 200 per group with subgroup breakdowns reported but no attrition/missing-data account (C3); groups compared with Mann-Whitney tests only, with age/sex balance noted but no multivariable confounder adjustment (C4); the sport exposure occurred as intended through regular training and competition periods (C5).
Martínez-Miguel et al., 2026	Quantitative descriptive (Cat.	Y	Y	Y	N	Y	Y	Y	Cross-sectional validation/agreement study; single-group method comparison of the My Jump Lab app against the NordBord during the Nordic hamstring exercise in 31 professional male soccer players (Cat. 4). An a priori sample size (n = 29 needed) was met, and validated devices with

Study (first author, year)	Design (MMAT category)	S1	S2	C1	C2	C3	C4	C5	Notes / justification
	4)								standardized 2D digitization and intra-rater reliability support appropriate measurements (C3) and analysis (Pearson correlations, regression, Bland-Altman with proportional-bias testing; C5). One second-division male team is not representative of the broader target population of athletes (C2); outcome data were complete for all 31 with no attrition (C3, C4).
Kuang et al., 2025	RCT (Cat. 2)	Y	Y	CT	Y	CT	N	Y	Two-group randomized controlled trial (n = 60) comparing the WorryTree CBT app against a no-intervention control on anxiety (BAI), mental health (GHQ-28) and academic performance over six months (Cat. 2). Random allocation is stated but no sequence generation or allocation concealment is described (C1); baseline anxiety and mental-health scores are closely matched between groups (C2); completeness/attrition across the three timepoints is not accounted for (C3). Outcomes are self-reported with no assessor blinding (C4); app engagement was tracked via weekly self-reports indicating adequate adherence (C5).
Ma et al., 2025	Quantitative descriptive (Cat. 4)	CT	CT	CT	N	CT	CT	Y	System-demonstration paper for a psychology-based table-tennis feedback app; the quantitative arm is a within-subjects video-rating comparison of pre- vs post-optimization designs (44 participants, 80 valid questionnaires) using IMI/NASA-TLX/SUS (Cat. 4). No clearly stated research question, and demo-video ratings bear only indirectly on whether the app improves real training (S1, S2). Online convenience recruitment with an unclear denominator gives an uncertain sampling frame (C1) and non-representative sample (C2); validated scales are applied indirectly to videos (C3) and nonresponse is uncertain (C4); paired t-tests are appropriate to the comparison actually performed (C5).
Marín et al., 2026	Quantitative non-randomized (Cat. 3)	Y	Y	CT	Y	Y	N	CT	Cross-sectional association study in 16 high-level male youth soccer players relating smartphone-assessed single-leg-deadlift core stability (OCTOcore app) to sprint velocity and V-cut change-of-direction speed (Cat. 3, non-randomized). Validated app and video-timing measures support appropriate measurement of exposure and outcomes (C2), and data were complete for all 16 (C3). The small single-club convenience sample limits representativeness (C1); no confounders (e.g., maturation, anthropometrics) were adjusted for beyond a median split (C4); the cross-sectional design has no true exposure period, so exposure-as-intended cannot be judged (C5).
Javaloyes et al., 2021	Quantitative descriptive (Cat. 4)	Y	Y	Y	N	Y	CT	Y	Observational single-group preliminary study describing HRV dynamics (HRV4training app, PPG) before, during and after an altitude camp in 7 professional female cyclists (Cat. 4). A validated app and power-based training-load measures support appropriate measurement (C3), and repeated-measures ANOVA with standardized mean differences and correlations suit the descriptive aim (C5). All cyclists came from one team, so the sample is not representative of the target population (C2); with n = 7 and a >=90% compliance inclusion rule, handling of nonresponse/missing daily measurements is unclear (C4).
McGuigan et al., 2023	Mixed methods (Cat. 5)	Y	Y	Y	Y	Y	Y	CT	Randomized mixed-methods study (control n = 19 vs education n = 16 recreational athletes) testing whether education improves adherence to a smartphone training-monitoring program, combining an RCT (adherence, ABQ, ARSS, perceived knowledge) with semi-structured interviews analyzed by inductive content analysis (Cat. 5). The rationale for combining components, their integration and interpretation are reasonable (C1-C3), and the null questionnaire findings are explicitly contrasted with positive interview perceptions (C4). Component quality is mixed: the quantitative arm had ~52% dropout with unblinded self-report, while the qualitative analysis used a single coder with peer debriefing (C5).
Menezes-Reis et al., 2024	Quantitative descriptive (Cat. 4)	Y	Y	Y	N	CT	N	Y	Cross-sectional descriptive study of 22 CrossFit practitioners describing injury prevalence (Nordic Musculoskeletal Questionnaire) and functional-test classifications from the PHAST and Clinometer smartphone apps; single group with no comparison. Convenience recruitment from CrossFit boxes fits the descriptive aim (C1), but the small self-selected sample from one microregion is not representative of the broader CrossFit population (C2). Measurement appropriateness is uncertain because the core instrument, PHAST, has no published validation, although its component tests and the Clinometer are individually validated (C3). The authors note that many athletes declined and that participants may have under-reported injuries for fear of harming the sport's image, giving a high nonresponse/response-bias risk (C4); descriptive statistics suit the prevalence aim (C5).
Maxin et al., 2024	Quantitative non-randomized (Cat. 3)	Y	Y	CT	Y	Y	CT	Y	Non-randomized analytic study using pupillary light reflex (PLR) parameters from a smartphone pupillometer (PupilScreen) plus machine learning to classify concussed vs. baseline recordings in Division I football players (n = 93 baseline, 11 SRC); the exposure–outcome framing with a comparison group places it in Category 3. Participants were drawn near-completely from one team, but the male-only, single-sport, single-institution frame and only 11 concussions limit representativeness of athletes with SRC (C1). SRC was physician-diagnosed per the Amsterdam consensus and the PLR captured with a previously validated app, so measurements are appropriate (C2), with complete outcome data on all concussed athletes (C3). PLR confounders are only partly handled — each athlete serves as their own baseline, but mood disorder differed significantly between groups (p = 0.01) and was not adjusted in the models (C4); the concussion exposure occurred and was measured within 24 h as intended (C5).
Moreno-Azze et al., 2025	Quantitative descriptive (Cat. 4)	Y	Y	Y	N	Y	Y	Y	Concurrent validity/reliability study comparing the Fitnessmeter iOS app against gold-standard photocells for the 5 + 5 m change-of-direction test in 46 trained adolescent male soccer players (180 trials); single-group validation, Cat. 4. Convenience sampling fits the validation aim (C1), but a single-sex, single-sport, single-academy sample does not represent the health, education and rehabilitation contexts the app targets (C2). Simultaneous app-vs-photocell capture with complete data across all four trials supports appropriate measurement (C3) and low nonresponse risk (C4); the statistical battery — Pearson r, linear regression, Bland–Altman, ICC (Koo & Li), CV and Durbin–Watson — is appropriate (C5).
Medeiros et al., 2024	Quantitative descriptive (Cat. 4)	Y	Y	Y	N	Y	Y	Y	Validity/reliability study of the My Jump 2 app versus a force platform (gold standard) for countermovement jumps on sand in 11 elite women beach volleyball players (66 jumps); single-group validation, Cat. 4. Sample size was justified a priori (GPower) and the elite population fits the sand-jump question (C1), but 11 elite champions do not represent the broader population that would use the app (C2). A 1000-Hz force

Study (first author, year)	Design (MMAT category)	S1	S2	C1	C2	C3	C4	C5	Notes / justification
									platform plus 240-fps app capture with complete data give appropriate measurements (C3) and low nonresponse risk (C4); the analysis — ICC, CCC, Bland–Altman, SEM, CV with bootstrap CIs, SWC and MDC — is thorough and appropriate (C5).
Maia da Silva et al., 2025	Quantitative descriptive (Cat. 4)	Y	Y	Y	CT	Y	N	Y	Concurrent validity/reliability study of the Jumpster app against My Jump 2 and a force platform for vertical jump in 53 recreational athletes (27 injured, 26 non-injured); single-group validation, Cat. 4. Convenience sampling with defined inclusion criteria fits the aim (C1), and the mixed-sex, multi-sport, injured/uninjured sample is only moderately representative given single-site recruitment skewed toward basketball (C2). Force platform and My Jump 2 references with two blinded evaluators support appropriate measurement (C3), but test–retest reliability — a primary outcome — was completed on only 19 of 53 athletes (36%) because of the COVID-19 pandemic, with no attrition analysis, a substantial nonresponse-bias risk (C4). The validity/reliability statistics are appropriate (C5); note that the Table 1 force-platform means are internally inconsistent (total 39.07 cm exceeds both subgroup means), likely a reporting error.
Mon-López & Tejero-González, 2019	Quantitative descriptive (Cat. 4)	Y	Y	Y	N	Y	CT	Y	Quantitative descriptive validation of the TargetScan ISSF Pistol & Rifle scoring app against a reference target-reading machine (Disag RMIV); 1440 shots from 24 shooters, single group, Cat. 4. Clear validity/reliability aim with an appropriate reference standard and reliability statistics (ICC, paired t, Bland-Altman) (C1, C3, C5). The sample is experienced national/international shooters and only 16.7% women, so it does not represent the amateur/semi-professional users the app is aimed at (C2). Recruitment of shooters and any nonresponse or selection are not described (C4).
Menaspà et al., 2018	Quantitative descriptive (Cat. 4)	Y	Y	Y	CT	Y	CT	Y	Quantitative descriptive criterion-validity study of an online athlete-management-system s-RPE against heart-rate-based load (Banister TRIMP, Edwards' method); 16 elite women water polo players, 303 sessions, single group, Cat. 4. Clear aim, appropriate criterion measures, and correlational analysis with confidence intervals (C1, C3, C5). The intact national squad is well sampled for that group, but representativeness beyond a single-sport, single-sex, elite population is uncertain (C2), and per-player session counts varied (13-27) with logging compliance deliberately unenforced and no analysis of missing entries, leaving nonresponse bias unaddressed (C4).
Mosley et al., 2024	Mixed methods (Cat. 5)	Y	Y	Y	N	CT	N	N	Mixed-methods evaluation of a smartphone slow-paced-breathing intervention in dual-career athletes: within-subject pre-post quantitative testing (n = 8; vmHRV, PHQ-ADS distress, wellbeing) plus a focus group analyzed by reflexive thematic analysis, Cat. 5. Clear aim with an adequate rationale for mixing (C1), and the qualitative strand is reasonably rigorous, but the two strands are reported largely in parallel rather than genuinely integrated (C2), so integration outputs are not distinctly interpreted (C3). The divergence between positive athlete perceptions and null/negative quantitative results (resting vmHRV actually decreased) is not reconciled (C4), and the quantitative component is weak - very small n, substantial missing data (3 of 8 diaries not returned; one incomplete vmHRV/Stroop dataset), and descriptive-only, visually inspected analysis - so not both traditions meet their quality criteria (C5).
Oommen et al., 2018	Quantitative descriptive (Cat. 4)	CT	CT	CT	N	Y	CT	N	Quantitative descriptive proof-of-concept of a smartphone swim-guidance prototype for blind swimmers; testing used 2 subjects (pre-recorded laps) and 3 sighted volunteers plus a flutter board, with no blind participants, Cat. 4. It is an engineering demonstration with no stated research question and largely anecdotal testing, so the screening items are uncertain (S1, S2). No real sampling strategy is described and its relevance to the blind-athlete aim is unclear (C1); the convenience sample of sighted volunteers is not representative of that target population (C2). The detection metrics (usable-frame %, end-of-lane success, false positives, depth error) are appropriate engineering measures (C3), but the analysis is descriptive success tallies with overgeneralized conclusions from very few trials (C5), and selection or nonresponse is not addressed (C4).
Steff et al., 2026	Quantitative non-randomized (Cat. 3)	Y	Y	Y	CT	CT	CT	CT	Quantitative non-randomized (quasi-experimental) pre-post controlled trial of a mobile-app coordination program versus regular basketball training in 12-14-year-old male players (experimental n = 20, control n = 20; intact school teams, not randomized), Cat. 3. Clear aim and hypothesis with appropriate mixed-design repeated-measures ANOVA and time-by-group interactions; participants match the defined target population (S1, S2, C1). The primary outcomes are app-based coordination tests the authors explicitly acknowledge are not validated instruments (C2); attrition or exclusion numbers from any initial pool are not reported (C3); baseline age and anthropometrics were comparable and the same coach trained both groups, but the acknowledged maturation confounder was unmeasured and unadjusted (C4); and intervention adherence or fidelity is not quantified (C5).
Nie et al., 2025	Quantitative descriptive (Cat. 4)	Y	Y	Y	N	Y	CT	Y	System validation / accuracy study (SoundTrackDB: 61 subjects, 363 treadmill sessions, 40 h) evaluating a contactless smartphone acoustic model for cadence and GCT against RunScribe and sports-watch baselines; Cat. 4. Clear aims and objectives with data that address them (S1, S2); dataset-driven sampling relevant to the estimation aim, spanning six paces, 24 treadmills and 13 gyms (C1), though a convenience sample (61 of 70 recruited, ages 18-55) not representative of the broader running population (C2); measurements appropriate, with CV-derived ground truth validated against three human annotators and MAE/MAPE reporting (C3); nonresponse/attrition only partly addressed as 9 excluded subjects (4 quit, 5 data loss) with no fuller account (C4); statistical analysis (MAE, error distributions, 9-fold cross-validation) appropriate to the aim (C5).
Park et al., 2017	Quantitative descriptive (Cat. 4)	CT	CT	CT	N	CT	N	N	Prototype system-demonstration paper (Motion Log Skateboard: pressure-sensor deck synced to video) with an experience session and interviews on 5 skateboarders; Cat. 4. No explicit research question or hypothesis and evaluation is largely anecdotal (S1, S2); very small convenience sample (n = 5, mixed skill) loosely appropriate to a proof-of-concept aim and not representative (C1, C2); no quantitative outcome measures, ground truth or validation of the logged pressure data reported (C3); nonresponse bias not addressed (C4); no statistical analysis, findings conveyed through illustrative quotes only (C5). A preliminary online survey (18 respondents) informs design but is not a formal qualitative method.

Study (first author, year)	Design (MMAT category)	S1	S2	C1	C2	C3	C4	C5	Notes / justification
van Iperen et al., 2022	RCT (Cat. 2)	Y	Y	CT	Y	CT	Y	N	Two-arm parallel randomized controlled trial (N = 425; intervention n = 214, control n = 211) of the REMBO self-regulation app versus no app in recreational long-distance runners; Cat. 2. Clear questions and pre-registered hypotheses with data addressing them (S1, S2); allocation via an online random-number generator but with no detail on sequence generation or allocation concealment (C1); groups comparable at baseline apart from a minor chance age difference (C2); substantial and differentially higher attrition in the intervention arm (up to ~46%) handled by intention-to-treat but without full missing-data accounting (C3); RRIs and chronic fatigue are self-reported with no assessor blinding, but self-report is the intended, validated outcome mode here rather than an unblinded proxy, so C4 = Y; adherence was low (40.2% used the app at least once), indicating many participants did not follow the assigned intervention (C5).
Oberhofer et al., 2021	Quantitative descriptive (Cat. 4)	Y	Y	Y	CT	Y	CT	Y	Single-group validation of the StrengthControl Apple Watch app against a GymAware linear position transducer (n = 30 recreationally trained athletes), with a clear validation aim and data on exercise recognition, repetition count and 1RM prediction (S1, S2). Convenience sample recruited by advertisement/email that the authors themselves flag as heterogeneous, limiting representativeness of the broader recreational population (C2). Recognition and repetition-count analyses excluded non-detected and falsely detected sets (~12%) and only 8.9% of app 1RM attempts succeeded, so some missing-data bias cannot be ruled out (C4); the reference instrument, ground-truth rep count and paired t-test/regression/correlation analyses were appropriate (C3, C5).
Moya-Ramon et al., 2022	Quantitative descriptive (Cat. 4)	Y	Y	Y	CT	Y	Y	Y	Method-comparison validity and reliability study of two HRV smartphone apps (Elite HRV with a chest strap; Welltory using PPG) against a 12-lead ECG gold standard in 26 professional cyclists, in supine and seated positions and at 5-min vs 1-min lengths, with a clear aim and concurrent multi-device data (S1, S2). The single-sport elite cohort only partially reflects the broader elite-endurance target population (C2). The ECG/Kubios reference, validated Polar H10 strap, repeated failed recordings, and comprehensive statistics (ANOVA, effect sizes, ICC, SEM, Bland-Altman) are appropriate with minimal missing data (C3, C4, C5).
Jalote-Parmar et al., 2024	Mixed methods (Cat. 5)	Y	Y	Y	CT	CT	N	CT	User-centred-design case combining formal qualitative user studies (focus groups, semi-structured interviews, contextual inquiry with affinity mapping) with a quantitative longitudinal field test (SUS, UEQ) of the Coral App (field test n = 26 female athletes and 1 coach), making it a mixed methods study (Cat. 5); the aim, data and rationale for the UCD/mixed design are adequate (S1, S2, C1). The quantitative and qualitative field-test strands are reported side by side rather than jointly analysed, so integration and its interpretation are only partial (C2, C3). A clear divergence-hormonal-IUD users reporting little benefit despite generally positive usability scores-is not reconciled (C4), and some collected data (self-reported menstrual-cycle, training and performance) are not reported, limiting adherence to each tradition's quality criteria (C5).
Rios-Gallardo et al., 2025	Quantitative descriptive (Cat. 4)	Y	Y	Y	N	Y	Y	Y	Single-session concurrent-validity and agreement study of the My Jump 2 app versus an infrared contact mat for squat- and countermovement-jump height and the Elastic Index (n = 14 male university basketball players), with a clear method-comparison aim and concurrent paired data (S1, S2). A deliberately homogeneous, small, single-sex, single-sport convenience sample that the authors state should not be generalised, giving low representativeness of any broader population (C2). Both devices derive height from flight time by standard ballistics, all athletes completed testing with invalid trials repeated, and the analyses (ICC, Bland-Altman, proportional-bias testing, TOST equivalence) are appropriate (C1, C3, C4, C5).
Renner et al., 2024	Quantitative descriptive (Cat. 4)	Y	Y	Y	Y	Y	CT	Y	Concurrent-validity study of three barbell-velocity apps (Qwik VBT, Metric VBT, MyLift) and one linear transducer (RepOne) against a Vicon 3D motion-capture gold standard in 20 competitive powerlifters (589 repetitions), with a clear aim, defined inclusion criteria and gold-standard measurements analysed with Bayesian mixed models (S1, S2, C1, C2, C3, C5). Metric VBT and MyLift failed to detect many repetitions (MyLift captured only ~16% of bench-press reps), so validity estimates for those apps rest on reduced, potentially non-representative subsets that the authors flag but cannot fully bound (C4).
Reynell & Thinyane, 2012	Quantitative descriptive (Cat. 4)	CT	CT	CT	N	CT	CT	N	Prototype/system-demonstration paper; single-group informal usability evaluation (n = 3 expert skateboarders) of the modified Tilt 'n Roll++ skateboard-visualisation system via semi-structured interviews. Aims are framed as engineering goals with no crisp research question and the interview data only loosely address system usefulness (S1, S2); recruitment was an informal convenience sample described as 'random' with an unclear denominator (C1) and three kickflip-capable experts do not represent the broader skateboarding population (C2); completeness/nonresponse cannot be judged (C4); findings are a narrative summary of three interviews with no statistical analysis (C5).
Romanenko et al., 2025	Quantitative descriptive (Cat. 4)	Y	Y	Y	CT	Y	Y	Y	Validation study developing and validating the 'Spatial Perception' iPad app in ITF taekwondo athletes, with test-retest reliability (n = 20, 3-week interval; Cronbach's alpha 0.96, Spearman, Bland-Altman) and a between-group validity comparison (n = 33 vs n = 24); single-group validation confirms Cat. 4. Clear aim and hypothesis with appropriate reliability/validity statistics (S1, S2, C3, C5); the sample is male, taekwondo-only from a single federation, so representativeness of the broader martial-arts target population is uncertain (C2); outcome data are complete with no attrition reported (C4).
Peng et al., 2024	Quantitative descriptive (Cat. 4)	Y	Y	Y	CT	Y	CT	Y	Concurrent validity/reliability study comparing the My Jump 2 app against a Kistler force-platform criterion for countermovement-jump height, flight time and interlimb symmetry (ICC, Cronbach's alpha, CV%, Pearson, Bland-Altman); single-group validation, Cat. 4. Sample size is reported inconsistently: the abstract states 79 participants but the Methods describe 29 athletes (12 fencing, 10 swimming, 7 diving; 87 jumps), which limits confidence in reported completeness (C4). The small, age-heterogeneous (11-27 yr) convenience sample across three sports limits representativeness (C2); measurement against the force-platform gold standard and the analyses are otherwise appropriate (C1, C3, C5), though the authors note an unusually high jump-height CV% (13.8-19.5%).

Study (first author, year)	Design (MMAT category)	S1	S2	C1	C2	C3	C4	C5	Notes / justification
Ríos-Gallardo et al., 2025	Quantitative descriptive (Cat. 4)	Y	Y	Y	CT	Y	Y	Y	Reliability/validity/agreement study of the My Jump Lab AI app (30 fps, bounding-box displacement) against a criterion force platform, an infrared timing system and manual 240 fps video in 80 male athletes across four cohorts, with a 7-10-day test-retest; single-group method comparison, Cat. 4. Clear objectives and hypothesis, rigorous simultaneous measurement and appropriate statistics: ICC(2,1), CV, SEM, Bland-Altman and OLP regression with proportional-bias testing (S1, S2, C3, C5); complete-case data with incomplete attendees and implausible values excluded and described (C4). The sample is male-only and university-based, which the authors acknowledge limits generalisability, so representativeness of the broader target population is uncertain (C2). NB: distinct from the other Rios-Gallardo et al. 2025 entry (My Jump 2 vs infrared contact mat, n = 14); assign a/b suffixes per the reference list.
Silva et al., 2026	Quantitative descriptive (Cat. 4)	Y	Y	CT	N	CT	CT	CT	Single-participant feasibility pilot of a smartphone pose-estimation app for three Shotokan Karate stances (n = 1; 285 frames for stance labels, 9 expert-annotated frames for angle error). The feasibility aim is explicitly stated and addressed with a predefined descriptive comparison (S1, S2); a single convenience participant with no sampling rationale (C1) is clearly not representative of the practitioner population (C2); reference angles come from one non-blinded instructor with no instrumented ground truth or reliability assessment (C3); nonresponse is not applicable/addressed (C4); the analysis is a descriptive MAE over 9 frames from one subject, which the authors themselves label illustrative only (C5).
Simpson et al., 2017	Quantitative non-randomized (Cat. 3)	Y	Y	CT	CT	CT	N	CT	Single-group pre-post feasibility intervention (n = 17 elite U21 male field hockey players; 6-week MealLogger programme with dietetic feedback and weekly education), analysed for exposure-outcome change, hence Cat. 3. One national squad is a narrow slice of the elite-athlete target population (C1); the knowledge questionnaire was previously validated but reworded with unknown effect on validity, and the exit survey was custom-built (C2); attrition and completeness of the post-test are not reported (C3); there is no control group or adjustment, so the pre-post knowledge change is unprotected against confounding (C4); logging compliance fell from 86% to 59% and engagement with education material was highly variable (C5).
Tan et al., 2024	Quantitative descriptive (Cat. 4)	Y	Y	Y	CT	Y	CT	Y	Cross-sectional concurrent validity/reliability study of the AI-driven My Jump 2 app against a 1,000-Hz force platform (n = 88 national-level athletes; 264 simultaneous countermovement jumps); single-group validation, Cat. 4. Clear aim addressed with paired data (S1, S2); recruitment through routine institute monitoring suits the validation aim (C1) but the single-country elite sample limits representativeness of wider users (C2); the force-platform criterion and defined methods are appropriate, though the impulse-momentum vs flight-time difference between devices is inherent (C3); exclusions/nonresponse are not addressed (C4); ICC, CV, Bland-Altman and ordinary-least-products regression are appropriate (C5).
Romanenko et al., 2024	Quantitative descriptive (Cat. 4)	Y	Y	Y	CT	CT	CT	N	Method-development and test-retest study of an iPad 'Go/No-Go' reaction app in ITF taekwondo athletes (n = 25 pilot; n = 16 retest at 3 weeks); distinct from the Romanenko et al. 2025 Spatial Perception entry. Sampling combat athletes is relevant to the aim (C1), but a single-academy taekwondo sample with a wide age range limits representativeness (C2); the measurement protocol is described but has no criterion comparison, and the app discards the worst attempt when data are non-normal, a potential bias (C3); the n = 16 retest subsample is unexplained and attrition unaddressed (C4); reliability/validity is inferred from non-significant Mann-Whitney comparisons rather than agreement statistics such as ICC or Bland-Altman, which does not answer the stated question (C5).
Tarnas et al., 2023	Quantitative descriptive (Cat. 4)	Y	Y	Y	CT	CT	CT	CT	Quantitative descriptive evaluation of real-time augmented audio feedback (SoniSailing) during on-water training (n = 13 RS:X windsurfers; 3 days, 9 upwind races, custom 8-item Likert questionnaire). Recruiting the available national-squad windsurfers is relevant to the aim (C1), but the sample is small with an author-acknowledged uneven distribution of sports levels (C2); the sole outcome is a custom, unvalidated questionnaire of subjective opinion (C3); full questionnaire response is implied but not explicitly reported (C4); Spearman and Mann-Whitney analyses are reasonable but exploratory in n = 13 with multiple uncorrected comparisons (C5).
Stöggl et al., 2014	Quantitative descriptive (Cat. 4)	Y	Y	Y	N	Y	CT	Y	Algorithm development/validation study (n = 11 competitive cross-country skiers, 7 M/4 F); smartphone accelerometer gear classification validated against synchronized video as the gold standard. Clear aim with data able to address it (S1, S2). Convenience sample of regional-to-international skiers only, single phone model, and no recreational/amateur skiers, so it is not representative of the broader target population the authors invoke (C2). Measurements and reference standard are appropriate (C3); attrition/nonresponse is not characterized (C4); confusion-matrix accuracy, paired t-test and correlation are appropriate to the validation aim (C1, C5).
Umek et al., 2014	Quantitative descriptive (Cat. 4)	CT	CT	CT	N	CT	CT	N	Prototype/system-demonstration paper (autonomous wearable golf-swing trainer). No explicit research question or hypothesis is stated and testing is anecdotal (S1, S2); no reported sample size or sampling frame (C1) and a non-representative, undescribed sample — one professional plus unspecified amateurs (C2). Measurement appropriateness cannot be judged against any stated question (C3); nonresponse not addressed (C4); no statistical analysis is performed (C5).
Zosimadis & Stamelos, 2023	Quantitative descriptive (Cat. 4)	Y	Y	Y	N	Y	Y	Y	System evaluation/validation study (n = 9 right-handed bowlers: 3 novice/3 intermediate/3 expert, 50 throws each = 450 throws), validated against coach labels and high-speed video. Clear objectives addressed by the data (S1, S2); skill-stratified recruitment is relevant to the classification aim (C1). Single club, all right-handed, five-step approach only — an acknowledged limitation making the sample non-representative of bowlers generally (C2). IMU/DTW/gait measurements appropriate (C3); all participants completed the full protocol with fouls accounted for, so nonresponse risk is low (C4); DTW scoring, paired t-tests, precision/recall and a cross-validated SVM are appropriate (C5), though Table 4's skill-classification figures contain apparent transcription errors.

Study (first author, year)	Design (MMAT category)	S1	S2	C1	C2	C3	C4	C5	Notes / justification
Zebis et al., 2019	RCT (Cat. 2)	Y	Y	Y	Y	Y	CT	Y	Randomized cross-sectional (single-bout parallel-group RCT); n = 47 female football/handball players randomized to app-based (n = 23) vs physiotherapist (n = 24) instruction, EMG and 3D-kinematic outcomes. Clear question with adequate data (S1, S2). Randomization stratified by sport using concealed opaque envelopes (C1); baseline characteristics comparable (Table 1, all P > 0.05; C2); all randomized participants analyzed with no post-randomization attrition (C3). Outcomes are objective and the statistical analyst was blinded, but participants and the test leader collecting data were not, so assessor blinding is only partial (C4); adherence to the assigned single-session instruction mode was complete (C5).
Wickramagedara et al., 2025	Quantitative descriptive (Cat. 4)	CT	CT	CT	N	N	CT	N	System-demonstration paper (SwimSight) evaluating a 1D+2D CNN ensemble on a pooled corpus of >2000 public freestyle/breaststroke videos with no comparison group (Cat. 4). No explicit research question is stated and the "injury-risk" labels are weak labels generated from the authors' own biomechanical threshold rules rather than observed injuries, so the data cannot address actual injury prediction (S1/S2 CT). Video sampling is not described against any target swimmer population (C1 CT) and the clips (no swimmer demographics, two strokes only) are not representative (C2 N); the outcome measurement is circular because the model is evaluated against the rule-derived labels it was trained to reproduce (C3 N). Nonresponse is not applicable/unclear for a video-corpus design (C4 CT), and although the subject-disjoint split and accuracy/recall/F1 are internally reasonable, they cannot answer the injury-prediction question given synthetic ground truth (C5 N).
Vriend et al., 2015	Quantitative descriptive (Cat. 4)	Y	Y	Y	N	Y	N	Y	Single-group RE-AIM process evaluation of the 'Versterk je Enkel' ankle-sprain prevention app using Google Analytics on ~25,781 users plus a voluntary in-app questionnaire (n=82); no comparison group (Cat. 4). Clear aim with data matched to reach/adoption/implementation (S1/S2 Y); analytics cover the whole user base so the sampling strategy is relevant (C1 Y), measures are appropriate (C3 Y), and descriptive statistics suit the aim (C5 Y). The authors themselves note questionnaire responders are self-selected and likely not representative (C2 N), and the very low questionnaire uptake relative to the user base makes nonresponse bias high (C4 N).
Williams et al., 2021	Quantitative non-randomized (Cat. 3)	Y	Y	Y	Y	Y	CT	Y	Single-arm repeated-measures study of 12 powerlifters (10 M / 2 F) testing whether smartphone HRV tracks an overload→taper training exposure and the reliability of morning vs pre-session HRV; no randomization or control group (Cat. 3). Clear aims, a validated ithlete HRV app, and a volume-load/sRPE-verified exposure delivered as intended (S1/S2/C2/C5 Y); participants meet defined powerlifting inclusion criteria (C1 Y) and the 3 excluded for protocol noncompliance are accounted for, leaving complete data on the analytic sample (C3 Y). The within-subject design controls stable confounders, but prior training/recovery status and non-training daily stressors are explicitly acknowledged as uncontrolled (C4 CT).
Young et al., 2023	Quantitative descriptive (Cat. 4)	Y	Y	Y	Y	Y	Y	Y	Single-group criterion-validation study of a BlazePose markerless smartphone pipeline against a 14-camera Vicon reference in 31 experienced runners (9,327 strides); no comparison group (Cat. 4). Clear validation aim with data able to address it (S1/S2 Y); the runner sample and side-view sampling are relevant and adequately representative of the healthy-runner target (C1/C2 Y), and measurements are appropriate, using ICC(2,1), Pearson, Bland–Altman and mean error against the gold standard (C3/C5 Y). No data dropout or loss occurred across participants, keeping nonresponse/attrition bias low (C4 Y).
Pérez-Castilla et al., 2021	Quantitative descriptive (Cat. 4)	Y	Y	Y	N	Y	Y	Y	Reliability and validity study of the iLOAD smartphone app for mean set velocity in the back squat and bench press (n = 20 males), repeated-measures against a linear velocity transducer (LVT) reference. Clear questions with adequate data (S1, S2); repeated-measures sampling across four loads is relevant to the reliability/validity aim (C1); the sample of physical-education students with limited resistance-training experience is acknowledged as not representative of the broader athletic population (C2); measurements are appropriate against a gold-standard LVT with complete data across sessions (C3, C4); comprehensive, appropriate reliability and validity statistics (CV, ES, correlations, Bland-Altman) support the aim (C5).
Sastre-Munar & Romero-Franco, 2024	Quantitative descriptive (Cat. 4)	Y	Y	Y	N	Y	CT	Y	Observational feasibility/descriptive study of the SaluTrack app collecting wellness, pain, urinary incontinence and catastrophizing data from 11 youth basketball players over 5 weeks. Clear aim with adequate data (S1, S2); sampling relevant to a feasibility test (C1) but a small single-center youth sample is not representative (C2); validated questionnaires (Hooper wellness, VAS, ICIQ-UI SF, PCS) are appropriate (C3); weekly completion was 90.9%, yet acknowledged recall bias and open-feedback response bias leave nonresponse uncertain (C4); descriptive mean ± SD analysis is appropriate to the feasibility aim (C5).
Suarez-Tijeras et al., 2023	Quantitative non-randomized (Cat. 3)	Y	Y	CT	Y	CT	N	Y	Observational cross-sectional correlational study (n = 11, from 15 recruited) relating resting HRV recorded with the HRV4Training app to Yo-Yo IR1 aerobic performance in youth futsal players. Clear questions and hypotheses with adequate data (S1, S2); small single-team convenience sample not representative of the broader population (C1); validated app (PPG) and validated Yo-Yo test are appropriate measures (C2); four of 15 excluded, including one dropped as an outlier to preserve normality, so attrition is described but the outlier removal is a concern (C3); simple Pearson correlations with no adjustment for confounders (C4); exposure (resting HRV) occurred as intended (C5).
Van Reijen et al., 2018	RCT (Cat. 2)	Y	Y	CT	Y	Y	N	Y	Randomized controlled trial (n = 220; "Strengthen your Ankle" App vs printed Booklet delivering an 8-week neuromuscular training program) with 12-month follow-up. Clear question with adequate data (S1, S2); randomization via a random number generator is stated but sequence generation and allocation concealment are not described (C1); baseline groups comparable (Table 2; C2); outcomes self-reported with monthly follow-up, intention-to-treat analysis and only one missing cost diary addressed (C3); self-reported outcomes with no assessor blinding (C4); adherence measured, with more than 70% highly compliant (C5).
Verlin et al., 2019	Quantitative	CT	CT	CT	N	CT	CT	N	Prototype/system-demonstration paper (PoloTrac iOS app) with anecdotal usability testing on five local water-polo community members and

Study (first author, year)	Design (MMAT category)	S1	S2	C1	C2	C3	C4	C5	Notes / justification
	descriptive (Cat. 4)								no stated research question or hypothesis (S1, S2); convenience sample of five, marginally relevant to a usability aim (C1) and not representative (C2); testing relies on informal observation and verbal comments rather than defined measurements (C3), with no nonresponse account (C4); no statistical analysis of the demonstration (C5).
Yamagiwa et al., 2014	Quantitative descriptive (Cat. 4)	CT	CT	CT	N	CT	CT	N	Prototype scoring-system paper (Ski Trainer, Android accelerometer app) evaluated anecdotally on a single middle-level skier before/after, with no stated research question (S1, S2); single-subject 'sample' is marginally relevant to a demonstration (C1) and not representative (C2); before/after accelerometer measurements are described but not validated as outcome measures (C3), with no nonresponse account (C4); no statistical analysis given the n = 1 descriptive design (C5).
Yamaguchi & Miura, 2016	Quantitative descriptive (Cat. 4)	CT	Y	CT	N	Y	CT	N	Validation/descriptive study of a smartphone image-processing app (iPhoneSG) estimating pitch velocity, with 10 measurements per frame rate compared to manual iMac measurement; the aim is implicit rather than a stated question (S1) though the data address measurement accuracy (S2); no human sampling frame or defined target population, so representativeness is not established and the app was often unable to detect the ball (C1, C2); comparison tables are complete (C3) but nonresponse is not characterized (C4); analysis reports only error averages with no formal validity/agreement statistics (C5).
Yang & Wang, 2026	RCT (Cat. 2)	Y	Y	CT	Y	CT	Y	CT	Framework/system paper for an AR mobile training assistant whose evaluation includes a genuine 6-week two-arm randomised controlled trial (§4.3): 30 badminton beginners randomised to AR-assisted feedback (EG, n=15) vs traditional video feedback (CG, n=15), with mixed-design ANOVA on skill outcomes (Cat. 2). Clear question with data able to address it (S1/S2 Y). Randomisation is stated but no sequence generation or allocation concealment is described (C1 CT); groups are equivalent at pre-test (C2 Y). Outcomes are reported across pre/mid/post for all 30 with no attrition flow or completeness account (C3 CT). The primary skill outcome is rated by a certified instructor blinded to grouping (C4 Y), though secondary NASA-TLX/SUS measures are self-report. Twelve standardised sessions are reported as delivered but no per-participant adherence/compliance data are given (C5 CT).
Mat Sanusi et al., 2021	Quantitative descriptive (Cat. 4)	Y	CT	N	N	CT	N	CT	Proof-of-concept classification of correct versus mimicked forehand strokes (LSTM on smartphone and Kinect data), accompanied by a single expert-coach interview; the primary design and aim are the quantitative classifier evaluation and the interview has no stated qualitative method or analysis, so Cat. 4 rather than mixed methods. Research questions are stated (S1) but the very small dataset (one participant, 510 strokes) barely allows RQ1 to be addressed and the authors note the smartphone result may be chance (S2). Single-participant convenience sample is a poor fit to the classification aim (C1) and unrepresentative (C2); multimodal sensing is well described but derives from one player with a pocket placement the authors flag as limiting (C3). No handling of small-sample or nonresponse bias and near-chance accuracy (C4); LSTM with 10-fold cross-validation is reasonable but its adequacy to the question is uncertain given data insufficiency (C5).
Romero-Franco et al., 2016	Quantitative descriptive (Cat. 4)	Y	Y	Y	N	Y	CT	Y	Concurrent validity and reliability of the MySprint iPhone app versus timing photocells and a radar gun in 12 highly trained male sprinters (72 sprints); single-group agreement study, so descriptive. Clear aims with adequate paired data (S1, S2); sampling of trained sprinters suits the validation aim (C1) but a small all-male elite sample is not representative of general users (C2). Simultaneous three-device capture with two independent observers makes measurements appropriate (C3); no dropout or nonresponse account (C4). Pearson r, ICC, SEE, Bland-Altman and CV are appropriate (C5).
Şentürk et al., 2025	Quantitative descriptive (Cat. 4)	Y	Y	Y	N	Y	CT	Y	Concurrent validity and intra-session reliability of the My Jump Lab app (automatic real-time detection) versus a dual force platform for countermovement jump height in 36 recreationally active men; single-group agreement study, so descriptive. Clear aims with adequate paired data (S1, S2); convenience sampling with explicit eligibility criteria fits the validation aim (C1) but an all-male recreational sample is not representative of elite athletes or women, as the authors note (C2). Simultaneous force-plate (1000 Hz) and 240-fps app capture over five repetitions is appropriate (C3); no dropout or nonresponse account (C4). ICC, Pearson r, mean difference, Bland-Altman, SEM, CV and SWC are appropriate (C5).
Soga et al., 2023	Quantitative descriptive (Cat. 4)	Y	Y	Y	N	Y	CT	CT	Validity and reliability of the Nordic Angle app versus 2D motion-analysis software for break-point angle during Nordic hamstring exercise in 7 healthy males (12 repetitions each); single-group agreement study, so descriptive. Clear aim with adequate data (S1, S2); an a priori sample-size calculation supports the sampling strategy for the validation aim (C1) but n = 7 forming two clusters is not representative and the authors warn validity may be overestimated (C2). The same iPhone video analyzed by both the app and Frame-DIAS with repeated within-day trials makes measurements appropriate (C3); all seven completed with no nonresponse issue reported, though flow detail is minimal (C4). Spearman correlation just missing significance (p = 0.052) plus Bland-Altman applied to non-normal data means the analysis is only partly adequate to the question (C5).
Spork et al., 2021	Quantitative descriptive (Cat. 4)	Y	Y	Y	Y	CT	N	Y	Intra- and inter-rater reliability of a smartphone hip-rotation range-of-motion test in 41 academy footballers (60 hips analyzed); single-group reliability study, so descriptive. Clear aim and hypothesis with adequate data (S1, S2); sampling deliberately mirrors real academy conditions and fits the aim (C1), and the age- and sport-specific sample reasonably represents the intended target population of academy players (C2). Two blinded, randomized testers with an a priori sample-size calculation make measurements appropriate (C3); substantial attrition (41 to 30 players, 11 dropouts for injury/illness/COVID) with no imputation raises nonresponse bias (C4). ICC(3,1), SEM, MDC and CV with normality and variance testing are appropriate (C5).
Tate & Milner, 2017	Quantitative	Y	Y	Y	N	Y	CT	Y	Single-group within-session pre/post evaluation of sound-intensity gait-retraining feedback in 14 healthy runners; no comparison group, so

Study (first author, year)	Design (MMAT category)	S1	S2	C1	C2	C3	C4	C5	Notes / justification
	descriptive (Cat. 4)								descriptive rather than an RCT or association study. Clear aim and hypothesis (S1, S2); a convenience student sample fits the proof-of-concept aim (C1) but is not representative of the broader or injured running population, as the authors acknowledge (C2). Force-plate measurement with five-trial averaging is appropriate (C3); all 14 recruited were analyzed but there is no attrition or consent-flow account (C4). Paired t-tests with an a priori power analysis are appropriate (C5).
Viyanon et al., 2016	Quantitative descriptive (Cat. 4)	CT	CT	N	N	CT	N	CT	Prototype system-demonstration of the SwingPong app classifying table-tennis strokes; training data from only four people (two national players and two trainers) and tested on nine users. No formally stated research question or hypothesis and testing is largely anecdotal (S1, S2); a tiny convenience training sample is poorly matched to a general-user aim (C1) and unrepresentative (C2). Wrist-mounted phone sensing with offline single-rater labeling is only loosely specified (C3); class imbalance and modest accuracies (77% forehand, 70% backhand) with no handling of misclassification or nonresponse (C4). J48 with cross-validation is described but its appropriateness to a stated question is unclear given the anecdotal framing (C5).
Wang et al., 2024	Quantitative descriptive (Cat. 4)	Y	Y	Y	N	Y	CT	Y	Single-group concurrent-validity and reliability study of the My Jump 2 app vs a Kistler force platform for vertical stiffness (Kvert) in n = 20 male college soccer/basketball players (240 drop jumps). Clear aim and matching data (S1, S2); convenience sample sized via an a priori power calculation and appropriate for a validation aim (C1) but drawn from two sports at one university, so not representative of the broader target population (C2); all 240 jumps completed with no attrition (C3); two evaluators plus device were raters with no assessor blinding described, though blinding is not clearly applicable to a concurrent instrument comparison (C4); statistics (ICC, CV, Pearson r, Bland-Altman, regression) appropriate to the question (C5).
Shang et al., 2025a	Quantitative descriptive (Cat. 4)	Y	Y	Y	N	CT	N	Y	Validation study of the Maturo smartphone app vs sport-science expert evaluation of maturation metrics in n = 103 youth athletes (9-17 yr; J Sport Health Sci). Clear aim/data (S1, S2); purposive/convenience sampling relevant to the validation aim (C1) but a single-region, mostly-male academy sample the authors flag as limiting generalizability (C2); an internal n discrepancy (76 M/27 F in the abstract vs 72/31 in Table 1) and no attrition account make outcome-data completeness unclear (C3); no blinding between Maturo and expert assessments and a stated fixed bias (Maturo underestimates biological age), giving meaningful measurement-bias risk (C4); analyses (ICC, Pearson, Bland-Altman, regression, kappa, TEM) appropriate (C5).
Shang et al., 2025b	Quantitative descriptive (Cat. 4)	Y	Y	Y	N	CT	N	Y	Validation study of the Maturo app vs expert evaluation in n = 41 youth basketball players (10-15 yr; E-balonmano). Clear aim/data (S1, S2); convenience sample fits the validation aim (C1) but is small, single-setting and sport-specific, so not representative (C2); no attrition account and a copy-pasted home-court-advantage passage in the introduction signal limited reporting rigor, so outcome-data completeness is unclear (C3); no blinding of Maturo vs expert and a stated systematic underestimation bias (C4); statistics (ICC, Pearson, Bland-Altman, kappa, regression, TEM) appropriate (C5). Appears to describe the same Maturo program and author group as Shang 2025a.
Spetz et al., 2025	Quantitative non-randomized (Cat. 3)	Y	Y	N	Y	N	CT	Y	One-year observational study of n = 20 elite endurance athletes examining associations between subjective Readiness Advisor app ratings and objective wearable/POMS counterparts (no intervention, no randomization). Clear questions with adequate data (S1, S2); a homogeneous national-team convenience sample is not representative of a broader target population (C1); measurements of both the exposure (subjective ratings) and outcomes (validated wearables, POMS) are appropriate (C2); substantial missing data documented but material (exercise data from 2 athletes and the first ~3.8 months for 6 athletes missing, CGM from 1, 221 training days excluded), so outcome data are incomplete (C3); confounders partly addressed via mixed models and partial regression but many external factors remain uncontrolled (C4); the exposure (daily monitoring) occurred as intended across the year (C5). Author/svexa conflict of interest noted.
Soares et al., 2023	Quantitative descriptive (Cat. 4)	Y	Y	Y	N	Y	CT	Y	Validity/reliability study of the My Jump 2 app vs a Chronojump contact platform for countermovement-jump height in n = 21 judokas (42 jumps). Clear aim and matching data (S1, S2); convenience sample with an a priori size estimate, appropriate to the validation aim (C1) but drawn from mixed competitive levels at one federation and not representative of the broader judo population (C2); all 42 trials analysed with no attrition (C3); two independent evaluators analysed the same videos with no blinding described, though not clearly applicable to an instrument comparison (C4); statistics (ICC, Pearson r, Bland-Altman, regression, t-tests) appropriate (C5).
Stojiljkovic et al., 2024	Quantitative descriptive (Cat. 4)	Y	Y	Y	N	Y	CT	Y	Validity/reliability study of the My Jump 2 app vs a Kistler Quattro force plate for drop-jump performance and interlimb asymmetry in n = 9 young female basketball players, with 1-week test-retest. Clear aim and matching data (S1, S2); very small convenience sample but backed by an a priori power analysis (7 required) and appropriate for the validation aim (C1); a single-team, single-sex, n = 9 sample is not representative of the broader population (C2); data complete with test-retest and inter/intra-rater analyses and no attrition (C3); multiple raters/devices used with no blinding described, though not clearly applicable to an instrument comparison (C4); statistics (ICC, Pearson r, Bland-Altman, MAPE, CV, paired t-tests) appropriate (C5). Authors note the small n limits statistical power for asymmetry p-values.

Table S4. Technical characteristics of the evaluated mHealth apps, by study.

Note: Studies are grouped by application category and ordered alphabetically within category. Intended operator (athlete self-operated, coach/practitioner-operated, or both) was coded from each study's reported app purpose, features, and testing protocol, following the coding scheme and category-level rationale described in Section 3.2; codes reflect who physically operates the app or device during data collection, not who the app is designed to benefit. Secondary function(s) (Measurement, Feedback, Intervention, and/or Decision-support; non-exclusive, one or more may apply) were coded from the same source text following the scheme described in Section 2.6; a cross-category summary is presented in Table S6 and discussed in Section 3.1. Remaining codes were derived from the app-characteristic data extracted from each full text (app type, primary purpose, key features, connected devices); app-store listings and vendor websites were not consulted. Instruments serving only as criterion standards (e.g. force platforms, timing gates, three-dimensional motion capture, dual-energy X-ray absorptiometry, linear transducers, electrocardiography) were not coded as app sensors, nor was hardware that only delivered output or performed computation. Attributes not stated in the source study were coded as not reported rather than inferred. Computational class: 1, manual digitization (user selects frames or landmarks; app performs arithmetic only); 2, deterministic or rule-based; 3, computer vision or pose estimation, method reported; 4, machine learning or pattern recognition, method reported; 5, automated processing, method not reported; 6, large language model or generative component. Abbreviations: Ext., external; HR, heart rate; ECG, electrocardiography; IMU, inertial measurement unit; LLM, large language model; NR, not reported; PPG, photoplethysmography; R-R, R-R interval; term., terminology.

Study	App	Intended operator	Application category	Secondary function(s)	Sensing modality	Sensor source	External hardware	Class	AI/ML term.	LLM	Processing	Coding note
Aranki 2017	RunningCoach	Athlete self-operated	Sports skill training	Decision-support; Feedback; Measurement	Accelerometer; GPS/GNSS; Camera; Ext. chest strap	Built-in + external	Heart rate chest strap	2	No	No	Hybrid	On-device cadence estimation; server dashboard
Aranki 2018	RunningCoach	Athlete self-operated	Sports skill training	Decision-support; Feedback; Measurement	Accelerometer; GPS/GNSS; Camera; Ext. chest strap	Built-in + external	Jarv Run Bluetooth HR chest strap	2	No	No	Hybrid	On-device cadence estimation; web dashboard
Bilal 2025	Not reported (unnamed React Native app; framework prototype)	Athlete self-operated	Sports skill training	Feedback	Ext. IMU	External only	ESP32 + MPU-6050 cue-mounted IMU	6	Yes	Yes	NR	GPT chatbot coaching layer over rule-based stroke scoring; only LLM in the review
Bilić 2023	SwingVision (SV) (v9.8.3)	Both	Sports skill training	Feedback; Measurement	Camera	Built-in only	None	5	Yes	No	NR	Vendor-labeled AI stroke/spin recognition; method proprietary
Boratto 2017	u4fit	Athlete self-operated	Sports skill training	Feedback; Measurement	GPS/GNSS; Ext. chest strap	Built-in + external	Optional external ECG / HR sensors	2	No	No	Server/cloud	Coach web dashboard; virtual trainer cues
Cabahug 2025	BESTGYM PoseApp	Athlete self-operated	Sports skill training	Measurement	Camera; LiDAR	Built-in only	None	3	Yes	No	On-device	ViTPose-h via CoreML named; on-device processing stated
Ha 2011	Not reported (self-developed iPhone Poomsae education app)	Athlete self-operated	Sports skill training	Feedback; Intervention; Measurement	None	None	None	2	No	No	NR	Instructional video delivery; view counter

Study	App	Intended operator	Application category	Secondary function(s)	Sensing modality	Sensor source	External hardware	Class	AI/ML term.	LLM	Processing	Coding note
Hadza 2026	MyJump2	Athlete self-operated	Sports skill training	Feedback; Measurement	Camera	Built-in only	None	1	No	No	NR	My Jump 2 repurposed for karate technique timing
Hsu 2025	ABCapp (AI-powered Badminton Coach app)	Athlete self-operated	Sports skill training	Feedback; Measurement	Camera	Built-in only	None	3	Yes	No	On-device	ST-GCN/MLP and MediaPipe named; TensorFlow Lite on-device processing stated
Janssen 2020	Inspirun (e-Coach)	Coach/practitioner-operated	Sports skill training	Feedback; Measurement	GPS/GNSS; Ext. chest strap	Built-in + external	Wahoo TICKR X HR chest strap	2	No	No	NR	Adaptive training schemes from HR, GPS and RPE
Ma 2025	Not reported (mainstream table tennis training app, unnamed; iterated prototype)	Athlete self-operated	Sports skill training	Feedback; Measurement	Camera	Built-in only	None	3	Yes	No	NR	Deep-learning skeletal tracking; adaptive feedback timing
Mat Sanusi 2021	Table Tennis Tutor (T3) — LHAPP4	Athlete self-operated	Sports skill training	Feedback	IMU	Built-in only	None	4	Yes	No	NR	LSTM neural network named; Kinect depth sensor is the comparator
Muslimin 2024	Not reported (Android-based digital volleyball skill test instrument)	Athlete self-operated	Sports skill training	Measurement	Ext. pressure/target	External only	Radar / vibrating target sensors (4) in test kit	2	No	No	NR	Sensor-instrumented serve/smash test kit
Oommen 2018	Wearable Electronic Swim Coach	Athlete self-operated	Sports skill training	Feedback; Measurement	Camera; Gyroscope	Built-in only	Bluetooth earphones (output only)	5	No	No	NR	Machine vision tracking and voice recognition; method not reported
Park 2017	Motion Log Skateboard	Athlete self-operated	Sports skill training	Measurement	Ext. pressure/target	External only	Skateboard deck 160-point pressure-sensor matrix via Bluetooth	2	No	No	NR	Foot-pressure heat map synced to video
Reynell 2012	Tilt 'n Roll++	Athlete self-operated	Sports skill training	Feedback; Measurement	Ext. IMU; Ext. pressure/target	External only	Sensor-fitted skateboard (accelerometer, gyroscope, force-sensing resistor) via Bluetooth	4	No	No	NR	Dynamic Time Warping trick detection named
Silva 2026	Not reported (unnamed Shotokan Karate posture app; Flutter/Dart, Google ML Kit)	Athlete self-operated	Sports skill training	Feedback	Camera	Built-in only	None	3	Yes	No	On-device	Google ML Kit / BlazePose (33 landmarks) named; rule-based stance classification; on-device stated
Steff 2026	Tapd: A	Athlete self-	Sports skill	Feedback;	Touchscreen	Built-	None	2	No	No	NR	Six commercial

Study	App	Intended operator	Application category	Secondary function(s)	Sensing modality	Sensor source	External hardware	Class	AI/ML term.	LLM	Processing	Coding note
	Coordination Game; Hand eye test; Tap Of War; Color Reaction; Sound Buttons/Instant Buttons; Lights: A Memory Game	operated	training	Measurement		in only						coordination/reaction games
Stöggl 2014	Not reported (custom Smartphone gear-classification application/algorithm)	Athlete self-operated	Sports skill training	Feedback; Measurement	Accelerometer	Built-in only	None	4	Yes	No	NR	Markov-chain / multivariate-Gaussian classifier named; 80 Hz chest-mounted
Tate 2017	SPLnFFT Noise Meter (v5.2)	Athlete self-operated	Sports skill training	Feedback; Measurement	Microphone	Built-in only	None	2	No	No	NR	Real-time decibel meter; generic noise-meter app
Umek 2014	Not reported (golf-swing trainer application; autonomous wearable personal training system)	Athlete self-operated	Sports skill training	Feedback; Measurement	IMU	Built-in only	wireless headphones (output only); laptop (compute only, developer version)	2	No	No	Hybrid	Head-mounted phone; laptop-side processing in the developer version
Viyanon 2016	SwingPong	Athlete self-operated	Sports skill training	Feedback; Measurement	IMU	Built-in only	None	5	No	No	NR	Stroke classification from built-in sensors; method not reported
Yamagiwa 2014	Ski Trainer	Athlete self-operated	Sports skill training	Feedback; Measurement	IMU	Built-in only	None	2	No	No	NR	200 Hz; deterministic tempo/symmetry/dynamicity scoring
Yang 2026	CLARIS-T (LitePoseNet-based prototype)	Athlete self-operated	Sports skill training	Feedback	Camera; IMU	Built-in only	None	3	Yes	No	NR	LitePoseNet named; AR overlay with adaptive visual feedback
Young 2023	Not reported (custom research app)	Athlete self-operated	Sports skill training	Measurement	Camera	Built-in only	Raspberry Pi 4 edge server (compute only)	3	Yes	No	Hybrid	BlazePose named; cloud/edge processing stated
Zosimadis 2023	Not reported (custom Android application)	Athlete self-operated	Sports skill training	Feedback; Measurement	Ext. IMU	External only	Two MetaMotion R BLE IMU sensors (wrist and foot)	5	No	No	Hybrid	On-device quality assessment plus cloud upload; method not reported
Balsalobre-Fernández 2017	Runmatic	Coach/practitioner-operated	Performance measurement	Measurement	Camera	Built-in only	None	1	No	No	NR	Manual frames; Morin spring-mass model applied deterministically
Balsalobre-Fernández 2018	PowerLift (v2.8)	Coach/practitioner-operated	Performance measurement	Measurement	Camera	Built-in only	None	1	No	No	NR	Manual frame selection; load-velocity 1RM estimation

Study	App	Intended operator	Application category	Secondary function(s)	Sensing modality	Sensor source	External hardware	Class	AI/ML term.	LLM	Processing	Coding note
Balsalobre-Fernández 2019	CODTimer	Coach/practitioner-operated	Performance measurement	Measurement	Camera	Built-in only	None	1	No	No	NR	240 fps frame-by-frame timing
Balsalobre-Fernández 2020	My Lift (v4.0)	Coach/practitioner-operated	Performance measurement	Measurement	Camera	Built-in only	None	5	No	No	NR	Automated computer-vision barbell tracking; method proprietary
Cetin 2021	My Lift (formerly Power Lift)	Coach/practitioner-operated	Performance measurement	Measurement	Camera	Built-in only	None	1	No	No	NR	Manual frame selection; load-velocity 1RM estimation
Chen 2021	COD timer	Coach/practitioner-operated	Performance measurement	Measurement	Camera	Built-in only	None	1	No	No	NR	240 fps manual frame selection
Chiu 2020	Not reported (self-developed Android app; unnamed)	Both	Performance measurement	Decision-support; Measurement	Accelerometer	Built-in only	None	2	No	No	On-device	On-device capture and storage; analysis offline in MATLAB, outside the app
Cirilo 2021	ABT (Assistant Boxer Training)	Both	Performance measurement	Decision-support; Feedback; Measurement	Accelerometer	Built-in only	None	2	No	No	Server/cloud	Punch detection from built-in accelerometer; BLE/cloud transmission to coach iPad
Marín 2026	OCTOcore (v1.8.4); MySprint used for sprint timing	Coach/practitioner-operated	Performance measurement	Feedback; Measurement	Accelerometer	Built-in only	None	2	No	No	NR	100 Hz built-in accelerometer; MySprint used separately for sprint timing
Mon-López 2019	TargetScan ISSF Pistol & Rifle	Coach/practitioner-operated	Performance measurement	Measurement	Camera	Built-in only	None	5	No	No	NR	Automatic target-sheet image-recognition scoring; method proprietary
Moreno-Azze 2025	Fitnessmeter (v3.3)	Coach/practitioner-operated	Performance measurement	Feedback; Measurement	Camera	Built-in only	None	5	No	No	NR	Real-time camera motion detection; method proprietary
Nie 2025	SoundTrack	Coach/practitioner-operated	Performance measurement	Feedback; Measurement	Microphone; IMU	Built-in only	None	5	Yes	No	On-device	On-device acoustic machine learning stated; architecture not reported
Oberhofer 2021	StrengthControl	Coach/practitioner-operated	Performance measurement	Feedback; Measurement	Ext. IMU	External only	Apple Watch Sport (accelerometer/IMU)	5	Yes	No	NR	Exercise recognition, rep counting, 1RM prediction; architecture not reported
Pérez-Castilla 2021	iLOAD	Coach/practitioner-operated	Performance measurement	Feedback; Measurement	None	None	None	2	No	No	NR	Manual inputs plus timer; automatic velocity calculation
Renner 2024	Qwik VBT; Metric VBT; MyLift (My	Coach/practitioner-operated	Performance measurement	Measurement	Camera	Built-in only	None	5	No	No	NR	Automated barbell tracking against weight-

Study	App	Intended operator	Application category	Secondary function(s)	Sensing modality	Sensor source	External hardware	Class	AI/ML term.	LLM	Processing	Coding note
	Jump Lab)											plate reference; method proprietary across three apps
Romanenko 2024	Reaction Go/No-go	Coach/practitioner-operated	Performance measurement	Measurement	Touchscreen	Built-in only	None	2	No	No	On-device	Go/No-Go task; on-device with JSON export
Romanenko 2025	Spatial Perception	Coach/practitioner-operated	Performance measurement	Measurement	Touchscreen	Built-in only	None	2	No	No	NR	2D/3D shape-comparison tasks; SceneKit rendering
Romero-Franco 2016	MySprint	Coach/practitioner-operated	Performance measurement	Measurement	Camera	Built-in only	None	1	No	No	NR	Frame-by-frame sprint split timing
Uysal 2023	CODTimer; Seconds Count; StopwatchCamera	Coach/practitioner-operated	Performance measurement	Measurement	Camera	Built-in only	None	1	No	No	NR	Manual frame-based timing across three apps (240 fps and 30 fps)
Yamaguchi 2016	iPhoneSG	Coach/practitioner-operated	Performance measurement	Measurement	Camera	Built-in only	None	3	No	No	On-device	OpenCV frame subtraction/binarization ball tracking named; on-device stated
Balsalobre-Fernández 2026	My Jump Lab	Coach/practitioner-operated	Vertical jump measurement	Measurement	Camera	Built-in only	None	1	No	No	NR	240 fps frame-by-frame take-off/landing selection
Bishop 2022	My Jump 2 (v5.0) and CODTimer (v2.0)	Coach/practitioner-operated	Vertical jump measurement	Measurement	Camera	Built-in only	None	1	No	No	NR	Manual frame selection; My Jump 2 + CODTimer
Çetin 2024	Jump Power	Coach/practitioner-operated	Vertical jump measurement	Measurement	Camera	Built-in only	None	5	No	No	NR	Face-detection-based measurement; method proprietary
Dias 2025	My Jump Lab; VertVision	Coach/practitioner-operated	Vertical jump measurement	Measurement	Camera	Built-in only	None	1	No	No	NR	My Jump Lab and VertVision, both frame-by-frame
Driller 2017	My Jump	Coach/practitioner-operated	Vertical jump measurement	Measurement	Camera	Built-in only	None	1	No	No	NR	240 Hz manual frame selection
Gallardo-Fuentes 2016	My Jump	Coach/practitioner-operated	Vertical jump measurement	Measurement	Camera	Built-in only	None	1	No	No	NR	240 fps manual frame selection
Lopez 2026	Not reported (unnamed prototype; dashboard screenshot shows "JumpTracker")	Coach/practitioner-operated	Vertical jump measurement	Feedback; Measurement	Microphone	Built-in only	None	5	Yes	No	NR	Adaptive switching between AI and classical algorithm; architecture not reported
Maia da Silva 2025	Jumpster	Coach/practitioner-operated	Vertical jump measurement	Measurement	Accelerometer	Built-in only	None	5	No	No	NR	Automatic jump height from built-in sensors; method not reported
Medeiros 2024	My Jump 2	Coach/practitione	Vertical jump	Measureme	Camera	Built-	None	1	No	No	NR	240 Hz user-selected

Study	App	Intended operator	Application category	Secondary function(s)	Sensing modality	Sensor source	External hardware	Class	AI/ML term.	LLM	Processing	Coding note
		r-operated	measurement	nt		in only						frames
Peng 2024	My Jump 2	Coach/practitioner-operated	Vertical jump measurement	Measurement	Camera	Built-in only	None	1	No	No	NR	240 Hz user-selected frames
Ríos-Gallardo 2025	My Jump 2	Coach/practitioner-operated	Vertical jump measurement	Measurement	Camera	Built-in only	None	1	No	No	NR	240 fps frame-by-frame; Elastic Index computation
Ríos-Gallardo 2025	My Jump Lab AI (v4.5.9)	Coach/practitioner-operated	Vertical jump measurement	Measurement	Camera	Built-in only	None	5	Yes	No	NR	Vendor-labeled AI bounding-box tracking at 30 fps; method proprietary
Soares 2023	My Jump 2	Coach/practitioner-operated	Vertical jump measurement	Measurement	Camera	Built-in only	None	1	No	No	NR	Slow-motion flight-time analysis
Stafylidis 2025	MyJump 2	Coach/practitioner-operated	Vertical jump measurement	Measurement	Camera	Built-in only	None	1	No	No	NR	240 fps slow-motion; CMJ/SJ/DJ modes
Stojiljković 2024	My Jump 2	Coach/practitioner-operated	Vertical jump measurement	Measurement	Camera	Built-in only	None	1	No	No	NR	120/240 fps slow-motion; contact and flight time
Sxentürk 2025	My Jump Lab	Coach/practitioner-operated	Vertical jump measurement	Feedback; Measurement	Camera	Built-in only	None	5	No	No	NR	Real-time automatic bounding-box detection; method proprietary
Tan 2024	My Jump 2 (My Jump Lab)	Coach/practitioner-operated	Vertical jump measurement	Measurement	Camera	Built-in only	None	5	Yes	No	NR	Vendor-labeled AI take-off/landing detection; method proprietary
Wang 2024	My Jump 2	Coach/practitioner-operated	Vertical jump measurement	Measurement	Camera	Built-in only	None	1	No	No	NR	240 Hz frame-by-frame
Alexandersen 2025	PM Reporter Pro (PMSys)	Athlete self-operated	Self-reported monitoring	Decision-support; Measurement	None	None	None	2	No	No	Server/cloud	Self-report only; online team/individual portal
Attigala 2019	Optio	Both	Self-reported monitoring	Decision-support; Intervention; Measurement	None	None	None	5	Yes	No	NR	Stress classifier and weight prediction; architecture not reported
Bertschy 2023	Titan Athlete (Integrated Bionics)	Athlete self-operated	Self-reported monitoring	Measurement	Ext. wearable	External only	Titan 1 vest-mounted GPS unit (10 Hz)	2	No	No	NR	Self-report app with wearable integration
Duignan 2019	Not reported (Mobile Athlete Self-Report Measure, M-ASRM; custom/commercial)	Athlete self-operated	Self-reported monitoring	Measurement	None	None	None	2	No	No	NR	Daily self-report only

Study	App	Intended operator	Application category	Secondary function(s)	Sensing modality	Sensor source	External hardware	Class	AI/ML term.	LLM	Processing	Coding note
Dupuit 2023	Not reported (custom online menstrual-status monitoring app)	Athlete self-operated	Self-reported monitoring	Measurement	None	None	None	2	No	No	Server/cloud	Custom online calendar-based menstrual monitoring application
Jalote Parmar 2024	Coral App	Athlete self-operated	Self-reported monitoring	Intervention; Measurement	None	None	None	2	No	No	Server/cloud	Athlete-coach data sharing; smartwatch integration planned but not implemented
Johansen 2020	PMSys (Reporter App)	Athlete self-operated	Self-reported monitoring	Decision-support; Measurement	None	None	None	5	Yes	No	Server/cloud	Team-level readiness prediction; cloud/AI backend stated; architecture not reported
Lebedev 2021	Not reported (prototype information system with mobile application)	Athlete self-operated	Self-reported monitoring	Measurement	Ext. wearable	External only	Blood pressure and HR devices; activity trackers	2	No	No	Server/cloud	Information system with integrated electronic health records
McGuigan 2023	TrainingPeaks (basic athlete account)	Athlete self-operated	Self-reported monitoring	Measurement	None	None	None	2	No	No	Server/cloud	Account-based platform; auto-upload disabled for the study
Menaspà 2018	Athlete Management System (SMARTABASE, Fusion Sport)	Athlete self-operated	Self-reported monitoring	Decision-support; Measurement	None	None	None	2	No	No	Server/cloud	Online s-RPE self-report; HR telemetry is the criterion, not an app component
Sastre-Munar 2024	SALUTRACK (SaluTrack)	Both	Self-reported monitoring	Measurement	None	None	None	2	No	No	Server/cloud	Periodic questionnaires; athlete-clinician messaging; dual user profiles
Spetz 2025	Readiness Advisor (RA) app	Both	Self-reported monitoring	Decision-support; Feedback; Measurement	Ext. wearable	External only	Garmin GPS watch/HR monitor; Oura ring	2	No	No	Server/cloud	Nine-item readiness score; coach dashboard
de Oliveira 2024	ITStriker	Athlete self-operated	Physiological measurement	Measurement	Ext. chest strap	External only	Polar H7 Bluetooth HR chest strap	2	No	No	NR	Beep-paced protocol; HRDP/threshold estimation
Flatt 2015	ithlete	Athlete self-operated	Physiological measurement	Measurement	Ext. chest strap	External only	ECG receiver (headphone slot) + Polar T-31 chest strap	2	No	No	NR	Ultrashort lnRMSSD acquisition
Flatt 2017	ithlete	Athlete self-operated	Physiological measurement	Measurement	Ext. optical PPG	External only	Pulse-wave finger sensor (headphone slot)	2	No	No	NR	HRV plus electronic wellness questionnaire

Study	App	Intended operator	Application category	Secondary function(s)	Sensing modality	Sensor source	External hardware	Class	AI/ML term.	LLM	Processing	Coding note
Flatt 2021	ithlete	Athlete self-operated	Physiological measurement	Measurement	Ext. optical PPG	External only	ithlete pulse-wave finger sensor (headphone slot)	2	No	No	NR	Catapult microsensor used separately, not an app component
Holmes 2020	ithlete; HRV4Training	Athlete self-operated	Physiological measurement	Measurement	Ext. optical PPG; Camera (PPG)	Built-in + external	ithlete pulse-wave finger sensor (hockey); none for HRV4Training (rowing)	2	No	No	Server/cloud	Web-based data storage stated
Iliadis 2021	Not reported (custom mHealth monitoring system; Vidavo SA)	Athlete self-operated	Physiological measurement	Feedback; Measurement	GPS/GNSS; Ext. wearable	Built-in + external	Zephyr BioHarness chest belt + OxyPro pulse oximeter	2	No	No	Server/cloud	GSM real-time transmission to monitoring centre
Javaloyes 2021	HRV4training	Athlete self-operated	Physiological measurement	Feedback; Measurement	Camera (PPG)	Built-in only	None	2	No	No	NR	Camera-flash PPG; optional Bluetooth chest strap not used
Johansson 2026	CameraHRV (v5.0.9)	Athlete self-operated	Physiological measurement	Measurement	Camera (PPG)	Built-in only	None	2	No	No	NR	Camera photoplethysmography; Polar H10 and ECG are comparators
Moya-Ramon 2022	Elite HRV; Welltory	Athlete self-operated	Physiological measurement	Measurement	Ext. chest strap; Camera (PPG)	Built-in + external	Polar H10 chest strap (Elite HRV)	2	No	No	NR	Two apps: chest-strap (Elite HRV) and camera PPG (Welltory)
Pereira 2020	HRV Expert (CardioMood)	Athlete self-operated	Physiological measurement	Measurement	Ext. chest strap	External only	Polar H10 chest strap (Bluetooth R-R recording)	2	No	No	NR	HRV index calculation from R-R intervals
Suarez-Tijeras 2023	HRV4Training	Athlete self-operated	Physiological measurement	Measurement	Camera (PPG)	Built-in only	None	2	No	No	NR	Camera-flash PPG
Williams 2021	ithlete	Athlete self-operated	Physiological measurement	Measurement	Ext. optical PPG	External only	ithlete pulse-wave finger sensor (headphone jack)	2	No	No	NR	HRV plus self-report
Babouras 2024	Custom app (Google MediaPipe / BlazePose; iOS ARKit version)	Coach/practitioner-operated	Musculoskeletal screening	Measurement	Camera; LiDAR	Built-in only	None	3	Yes	No	NR	MediaPipe / BlazePose named; Kalman filtering
Martínez-Miguel 2026	My Jump Lab (version 5.0)	Coach/practitioner-operated	Musculoskeletal screening	Measurement	Camera	Built-in only	None	1	No	No	NR	240 Hz manual digitization of anatomical landmarks; model-based torque estimation
Maxin 2024	PupilScreen (Apertur Inc.)	Coach/practitioner-operated	Musculoskeletal screening	Measurement	Camera	Built-in only	None	5	Yes	No	NR	Computer-vision pupil detection plus ML classification; architecture not reported; 3D-printed box attachment

Study	App	Intended operator	Application category	Secondary function(s)	Sensing modality	Sensor source	External hardware	Class	AI/ML term.	LLM	Processing	Coding note
												standardizes light/distance
Menezes-Reis 2024	PHAST (Physiotherapy Assessment Tool); Clinometer (v2.4)	Coach/practitioner-operated	Musculoskeletal screening	Feedback; Measurement	IMU	Built-in only	None	2	No	No	NR	PHAST rule-based Good/Moderate/Bad classification; Clinometer gravity-sensor goniometry
Soga 2023	Nordic Angle	Coach/practitioner-operated	Musculoskeletal screening	Feedback; Measurement	Camera	Built-in only	None	5	No	No	NR	Automatic break-point angle calculation; method not reported
Spork 2021	yROM	Coach/practitioner-operated	Musculoskeletal screening	Measurement	IMU	Built-in only	None	2	No	No	NR	Built-in inertial goniometry; phone strapped to lower leg
Van Reijen 2018	Strengthen your Ankle (Versterk je enkel)	Athlete self-operated	Musculoskeletal screening	Feedback; Intervention; Measurement	None	None	None	2	No	No	NR	Non-connected balance board supplied as training equipment, not an app component
Vriend 2015	Versterk je Enkel ('Strengthen your ankle')	Athlete self-operated	Musculoskeletal screening	Feedback; Intervention; Measurement	None	None	None	2	No	No	NR	8-week neuromuscular programme delivery
Wickramagedara 2025	SwimSight	Coach/practitioner-operated	Musculoskeletal screening	Intervention; Measurement	Camera	Built-in only	None	5	Yes	No	NR	Pose estimation, injury-risk prediction and clustering from uploaded video; architecture not reported
Zebis 2019	Based on 'Get Set—Train Smarter'	Coach/practitioner-operated	Musculoskeletal screening	Feedback; Intervention; Measurement	None	None	None	2	No	No	NR	Video-based exercise instruction only
Heilmann 2023	Fruit Ninja	Athlete self-operated	Psychological intervention	Intervention	Touchscreen	Built-in only	None	2	No	No	NR	Commercial game repurposed as cognitive training
Kuang 2025	WorryTree: Anxiety CBT Therapy	Athlete self-operated	Psychological intervention	Feedback; Intervention	None	None	None	2	No	No	NR	Algorithmic personalized feedback; no method reported
Mosley 2024	BreathPacer	Athlete self-operated	Psychological intervention	Intervention	None	None	None	2	No	No	NR	Visual breathing pacer; ECG used separately for screening
Ruud 2025	TalentCards (REBT-inspired micro-learning content)	Athlete self-operated	Psychological intervention	Intervention	None	None	None	2	No	No	Server/cloud	TalentCards micro-learning platform; app/email reminders

Study	App	Intended operator	Application category	Secondary function(s)	Sensing modality	Sensor source	External hardware	Clas s	AI/M L term.	LL M	Processing	Coding note
van Iperen 2022	REMBO (Running and Exercise Mental Break Optimization)	Athlete self-operated	Psychological intervention	Feedback; Intervention	None	None	None	2	No	No	NR	12-item load-capacity test; traffic-light feedback
AlKasasbeh 2025	MyFitnessPal	Athlete self-operated	Nutrition	Feedback; Intervention	None	None	None	2	No	No	NR	Food logging and education modules; manual entry
Kholboeva 2025	NutriFit-AI	Athlete self-operated	Nutrition	Feedback; Intervention	Camera	Built-in only	None	5	Yes	No	NR	Author-labeled AI meal planning and computer-vision food logging; no architecture reported
Simpson 2017	MealLogger (MealLogger-Photo Food Journal, v4.6)	Athlete self-operated	Nutrition	Feedback; Intervention	Camera	Built-in only	None	2	No	No	Server/clou d	Image-based food diary; feedback is human dietitian, not automated
Haynes 2025	MeThreeSixty (Size Stream)	Coach/practitione r-operated	Anthropometri c and maturation screening	Measureme nt	Camera	Built-in only	None	5	No	No	Server/clou d	Automated digital anthropometry from two 2D images; cloud processing stated
Shang 2025	Maturo	Coach/practitione r-operated	Anthropometri c and maturation screening	Measureme nt	Camera	Built-in only	None	5	Yes	No	NR	Vendor-labeled AI and computer vision from photos; method proprietary; developer-affiliated study
Shang 2025	Maturo	Coach/practitione r-operated	Anthropometri c and maturation screening	Measureme nt	Camera	Built-in only	None	5	Yes	No	NR	Vendor-labeled AI and computer vision from photos; method proprietary; developer-affiliated study
Tarnas 2023	SoniSailing	Both	Tactical and match analysis	Decision-support; Feedback; Measureme nt	GPS/GNSS	Built-in only	bone-conduction headphones (output only)	2	No	No	NR	1 Hz GNSS; deterministic no-go-zone geometry and sonification
Verlin 2019	PoloTrac	Coach/practitione r-operated	Tactical and match analysis	Decision-support; Feedback; Measureme nt	None	None	None	4	No	No	NR	Manual live event entry; logistic-regression shot-probability model named

Table S5. PRISMA extension for scoping reviews (PRISMA-ScR) checklist.

Checklist items follow Tricco et al. (2018), PRISMA Extension for Scoping Reviews (PRISMA-ScR): Checklist and Explanation, Annals of Internal Medicine, 169(7), 467–473, https://doi.org/10.7326/M18-0850. Status is shaded green (Reported), amber (Partially reported), or red (Not yet reported). Items 12 and 16 (critical appraisal of individual sources of evidence) are optional under PRISMA-ScR; this review performed critical appraisal using the Mixed Methods Appraisal Tool (Section 2.8).

No.	Section/Topic	Checklist Item	Location in Manuscript	Status
1	TITLE	Identify the report as a scoping review.	Title	Reported
2	ABSTRACT	Provide a structured summary including background, objectives, eligibility criteria, sources of evidence, charting methods, results, and conclusions.	Abstract	Reported
3	INTRODUCTION	Describe the rationale for the review in the context of what is already known; explain why the review questions/objectives lend themselves to a scoping review approach.	Section 1, paragraphs describing the limited scope of prior single-domain reviews	Reported
4	INTRODUCTION	Provide an explicit statement of the questions/objectives, with reference to key elements (population/participants, concept, context).	Section 1, five numbered aims, plus explicit population/concept/context statement added at the end of the aims paragraph	Reported
5	METHODS	Indicate whether a review protocol exists, state where it can be accessed, and provide registration information.	Section 2.1 (OSF registration, DOI provided)	Reported
6	METHODS	Specify characteristics of the sources of evidence used as eligibility criteria, and provide a rationale.	Section 2.3	Reported
7	METHODS	Describe all information sources in the search, including dates of coverage.	Section 2.2; Table S1	Reported
8	METHODS	Present the full electronic search strategy for at least one database, including all limits used.	Table S1 (full search strings for all five databases)	Reported
9	METHODS	State the process for selecting sources of evidence (screening and eligibility).	Section 2.4	Reported
10	METHODS	Describe the methods of charting data from included sources, and any processes for obtaining/confirming data from investigators.	Section 2.5	Reported
11	METHODS	List and define all variables for which data were sought.	Section 2.5 (six extraction domains)	Reported
12	METHODS	If done, provide a rationale for critical appraisal of included sources, and describe the methods used.	Section 2.8 (MMAT); scope and limitations of this appraisal for measurement-property studies now specified	Reported
13	METHODS	Describe the methods of handling and summarizing the data that were charted.	Section 2.8	Reported
14	RESULTS	Give numbers of sources screened, assessed for eligibility, and included, with reasons for exclusion, ideally with a flow diagram.	Section 3.1; Figure 1 (PRISMA flow diagram)	Reported
15	RESULTS	For each source of evidence, present characteristics for which data were charted, and provide citations.	Table 2; Table S2 (per-study characteristics, including age and sex)	Reported
16	RESULTS	If done, present data on critical appraisal of included sources of evidence.	Table S3 (S3a summary frequencies; S3b per-study ratings); Section 4.5	Reported
17	RESULTS	For each included source of evidence, present the relevant data charted that relate to the review questions/objectives.	Table S2 (per-study outcomes)	Reported
18	RESULTS	Summarize and/or present the charting results as they relate to the review questions and objectives.	Sections 3.2–3.13; Figures 2–6	Reported
19	DISCUSSION	Summarize the main results, link to the review questions/objectives, and consider relevance to key groups.	Section 4.1	Reported
20	DISCUSSION	Discuss the limitations of the scoping review process.	Section 4.10	Reported
21	DISCUSSION	Provide a general interpretation of the results with respect to the review questions/objectives, and consider implications for future research, practice, and/or policy.	Section 5	Reported
22	FUNDING	Describe sources of funding for the included sources of evidence and for the review itself; describe the role of the funders.	Back matter between Conclusion (Section 5) and References: Author Contributions, Funding, Institutional Review Board Statement, Informed Consent Statement, Data Availability Statement, and Conflicts of Interest	Reported

Table S6. Cross-category matrix of secondary app function.

Each of the 111 included studies was assigned exactly one primary application category (Table 1), but apps frequently serve more than one function. This table reports, for each primary category, the number of studies whose app also carries each of four non-exclusive secondary-function tags, coded from the same purpose/features text used for the primary classification (Section 2.6): Measurement/Assessment (quantifies a physical, physiological, or psychological variable), Feedback/Technique guidance (provides real-time or session-level feedback to improve technique or skill), Intervention/Behavior change (delivers a structured program intended to change an outcome), and Decision-support (aggregates data into a coach- or practitioner-facing dashboard or recommendation). Because tags are non-exclusive, row totals exceed the category n where studies carry more than one function. Full per-study tags are provided in Table S4.

Application category	n	Measurement	Feedback	Intervention	Decision-support	Studies with >1 function (%)
Sports skill training	26	22	22	1	2	18 (69%)
Performance measurement	20	20	6	0	2	7 (35%)
Vertical jump measurement	18	18	2	0	0	2 (11%)
Self-reported monitoring	12	12	1	2	5	6 (50%)
Physiological measurement	12	12	2	0	0	2 (17%)
Musculoskeletal screening	10	10	5	4	0	6 (60%)
Psychological intervention	5	0	2	5	0	2 (40%)
Nutrition	3	0	3	3	0	3 (100%)
Anthropometric / maturation screening	3	3	0	0	0	0 (0%)
Tactical / match analysis	2	2	2	0	2	2 (100%)
**Total**	**111**	**99**	**45**	**15**	**11**	**48 (43%)**
